# Transcriptomics Analysis of Porcine Caudal Dorsal Root Ganglia in Tail Amputated Pigs Shows Long-Term Effects on Many Pain-Associated Genes

**DOI:** 10.3389/fvets.2019.00314

**Published:** 2019-09-18

**Authors:** Dale A. Sandercock, Mark W. Barnett, Jennifer E. Coe, Alison C. Downing, Ajit J. Nirmal, Pierpaolo Di Giminiani, Sandra A. Edwards, Tom C. Freeman

**Affiliations:** ^1^Animal and Veterinary Science Research Group, Scotland's Rural College, Roslin Institute Building, Edinburgh, United Kingdom; ^2^The Roslin Institute and Royal (Dick) School of Veterinary Studies, University of Edinburgh, Edinburgh, United Kingdom; ^3^Edinburgh Genomics, The University of Edinburgh, Edinburgh, United Kingdom; ^4^School of Natural and Environmental Sciences, Newcastle University, Newcastle upon Tyne, United Kingdom

**Keywords:** pig, tail amputation, tail docking, inflammatory pain, neuropathic pain, gene expression, animal welfare

## Abstract

Tail amputation by tail docking or as an extreme consequence of tail biting in commercial pig production potentially has serious implications for animal welfare. Tail amputation causes peripheral nerve injury that might be associated with lasting chronic pain. The aim of this study was to investigate the short- and long-term effects of tail amputation in pigs on caudal DRG gene expression at different stages of development, particularly in relation to genes associated with nociception and pain. Microarrays were used to analyse whole DRG transcriptomes from tail amputated and sham-treated pigs 1, 8, and 16 weeks following tail treatment at either 3 or 63 days of age (8 pigs/treatment/age/time after treatment; *n* = 96). Tail amputation induced marked changes in gene expression (up and down) compared to sham-treated intact controls for all treatment ages and time points after tail treatment. Sustained changes in gene expression in tail amputated pigs were still evident 4 months after tail injury. Gene correlation network analysis revealed two co-expression clusters associated with amputation: Cluster A (759 down-regulated) and Cluster B (273 up-regulated) genes. Gene ontology (GO) enrichment analysis identified 124 genes in Cluster A and 61 genes in Cluster B associated with both “inflammatory pain” and “neuropathic pain.” In Cluster A, gene family members of ion channels e.g., voltage-gated potassium channels (VGPC) and receptors e.g., GABA receptors, were significantly down-regulated compared to shams, both of which are linked to increased peripheral nerve excitability after axotomy. Up-regulated gene families in Cluster B were linked to transcriptional regulation, inflammation, tissue remodeling, and regulatory neuropeptide activity. These findings, demonstrate that tail amputation causes sustained transcriptomic expression changes in caudal DRG cells involved in inflammatory and neuropathic pain pathways.

## Introduction

Tail docking, amputation of approximately half to two-thirds of the tail, is a common practice in commercial pig production in newly born piglets, as a preventative measure against the issue of tail biting. This is an undesirable production-related abnormal behavior performed by pigs reared under sub-optimal environmental conditions and is often associated with poor health status (1). However, even tail docking is not a total safeguard against the loss of a portion of the tail under conditions of severe tail biting, which means that pigs can experience traumatic tail “amputation” through a portion of the tail being bitten off later in life (2). Concerns exist about the effects of tail amputation injury with respect to the initiation of short (acute) and long-term (chronic) pain in the tail stump (3–8). Tail amputation injury typically involves the severing of the caudal nerves in the tail which lead to the formation of “traumatic” or “amputation” neuromas in the tail stump (9). Traumatic neuromas are defined as truncated, tangled non-neoplastic proliferations of epineurial, perineurial, and endoneurial connective tissue, Schwann cells and axons which are attempting to regenerate after nerve transection (10). In humans, the development of traumatic neuromas after amputation-induced nerve injury can be a significant cause of pain and is associated with the phenomenon of residual stump pain and phantom limb pain, which is classified as a neuropathic pain state (11, 12). Neuropathic pain is initiated or caused by primary lesions to the somatosensory system. This leads to structural and functional neuronal changes which result in spontaneously occurring pain and amplified responses to innocuous and noxious stimuli (13). Measurements on tail amputated pigs carried out as part of this study, demonstrated that tail amputation leads to traumatic neuroma development and proliferation, that was still on-going 4 months after tail amputation injury (4). This was associated with acute and long-term changes in tail stump sensitivity, resembling neuropathic pain (7).

Primary sensory neurons are responsible for the detection, transduction, and transmission of painful and non-painful stimuli. Their cell bodies (soma) reside in dorsal root ganglia (DRG), from where they mediate axonal conduction of action potential signals to the central nervous system via the dorsal spinal cord (14). Peripheral nerve injury lowers the activation threshold required to evoke action potential firing in primary sensory neurons and can also cause spontaneous or ectopic neuronal firing long after the removal of the stimulus (15–17). Such changes in somatosensory nerve function are linked to neuropathic pain and are commonly observed in traumatic neuromas (18).

In the pig tail, sensory neurons are located in four caudal nerves (two dorsal and two ventral, left and right) that innervate all the way to the distal tip of the tail (19). The cell bodies of the caudal nerve somatosensory neurons aggregate in four corresponding DRG's situated at the proximal confluence of the cauda equina located within the sacrum, some distance away from the region of tail amputation injury (ca. 7–9 caudal vertebrae). Tail amputation injury in an appendage with this level of sensory innervation is likely to cause acute and possible long-lasting tail stump pain, especially where traumatic neuroma development also present.

It has long been recognized that changes in pain sensitivity and the presence of on-going chronic pain associated with traumatic peripheral nerve injury are linked to marked changes in the expression of neuropeptides and their receptors in primary sensory neurons (20). A number of studies in rodents have been undertaken investigating the effects of peripheral nerve injury on changes in gene expression in DRG neurons (21–25). From these studies it is clear that the pathophysiology of neuropathic pain is complex and is linked to changes in neurotransmission coupled to changes in immune activation and neuroinflammation. It is currently not known if similar changes occur in caudal nerve DRG sensory neurons in response to tail amputation injury. Therefore, the aim of this study was to investigate the transcriptional responses of the pig peripheral nervous system to traumatic injury caused by tail amputation. In particular, data analysis was undertaken to specifically examine changes in DRG gene expression linked to inflammatory and neuropathic pain signaling pathways.

## Materials and Methods

### Animal and Housing

All animal procedures were carried out under license (PPL 70/7919) granted under the Animal (Scientific Procedures) Act and approved by the Animal Welfare Ethical Review Boards of Newcastle University and Scotland's Rural College. Ninety-six female pigs (Landrace/Large White x synthetic sire line) from a resident herd at Cockle Park Farm, Newcastle University were used in the study. The pigs were maintained under standard housing conditions with *ad libitum* access to feed and water from selection at the time of farrowing until 1 week prior to the start of any experimental treatment. Optimal farrowing and post-weaning pen temperatures were thermostatically maintained throughout the duration of the experimental period.

### Experimental Design (Animal)

In order to investigate the effects of tail amputation on gene expression, 96 pigs were studied within a balanced, factorial (2 × 2 × 3) study, incorporating two tail treatments: (1) tail amputation and (2) sham amputation (intact tail controls), two tail treatment ages (3 and 63 days), and three post-tail-treatment times (+1, +8, and +16 weeks). This experimental design provided 8 pigs/tail treatment/treatment age/time after treatment.

Tail amputation was conducted on two groups of pigs at two distinct ages: (1) 3 days post-natal, consistent with tail docking of piglets in commercial pig production, and (2) 63 days of age, concomitant with the age range associated with the onset of severe tail biting in weaner pigs (26). In the study, animals undergoing tail amputation at 63 days of age retained intact tails until the time of surgical amputation of the tail under general anesthesia. Animals identified with any tail biting or body injuries at any time prior to experimental tail amputation treatment at 3 or 63 days were excluded from the study (*n* = 1: bitten tail).

#### Tail Docking

Piglets were tail docked at 3 days of age in line with commercial practice using a gas-heated docking iron (East Riding Farm Services, Driffield, UK). Two-thirds of the tail was removed, based on the measurement of intact tail length. Sham tail amputated, control piglets were handled in the same way as the tail docked animals but left with an intact tail. All docked piglets were observed over the following weeks as part of routine husbandry procedures for signs of post-amputation tail infection. All amputated tail stumps appeared superficially to heal normally without complications.

#### Surgical Tail Amputation Procedures

In pigs undergoing tail amputation at 63 days of age, such tail removal had to be carried out under general anesthesia in accordance with UK and European animal welfare regulations on the protection of animal used for scientific purposes. Food was withheld for a minimum of 8 h before surgical tail amputation. On the day of surgery, pigs housed in one pen were collectively transferred to a purpose-built pig holding unit equipped with holding pens and a veterinary surgical theater. Prior to surgery, animals were individually weighed and sedated using an intramuscular combined injection of ketamine (5 mg/kg, Vetoquinol, Buckingham, UK), midazolam (0.5 mg/kg, Hameln, Gloucester, UK), and medetomidine (10 μg/kg, Vetoquinol, Buckingham, UK). Following injection, the animals were left undisturbed in an individual pen and transferred to the surgical theater once adequate sedation was achieved, i.e., loss of righting reflex. Each pig was placed in lateral recumbency on the operating table covered with a thermostatically controlled heat blanket and anesthesia was administered and maintained using isoflurane (3–4%) (Abbot Laboratories Ltd, UK) delivered in 100% oxygen. During anesthesia, blood O_2_ saturation and body temperature were continually monitored. The tail length was measured to determine the location of the site of amputation. The tail was thoroughly cleaned and swabbed with 7.5% antiseptic povidone-iodine solution (Ecolab, UK) and a tourniquet was placed at the base of the tail to reduce blood flow. Loss of tail reflex was verified before surgical amputation was performed using a Liston bone cutter (World Precision Instrument, UK). Pinpoint hemostasis was performed with a high temperature cautery pen (Bovie Medical Corporation, USA) and wound powder was applied to the amputated, distal tail stump. Once tail resection was completed the pigs received an intramuscular injection into the neck muscle of atipamezole (5 mg/kg, Vetoquinol, UK), meloxicam (0.2 mg/kg, Boehringer Ingelheim, UK), and Penicillin-Streptomycin (0.04 ml/kg, Norbrook, UK), and anesthesia was ceased. At the end of the procedure, each pig was covered with a thermal blanket and transferred to an individual recovery pen measuring 2 × 2 m containing deep clean straw as bedding material and a water drinker. For 2 days after tail surgery, pigs were housed individually in adjacent recovery pens that allowed visual, auditory, and olfactory contact but prevented physical interaction with other animals. The animals designated to the “intact” treatment were exposed to the same protocol, except for the amputation of a portion of the tail and the administration of post-surgery drugs. The health of the animals was continually monitored during recovery by a trained operator along with regular inspections by the site veterinarians.

#### Sedation and Humane Killing

Post-mortem tissue collections were carried out on pigs 1, 8, and 16 weeks after tail treatment. Pigs were weighed to determine the required doses for sedation and euthanasia and sedated in a treatment pen with Stresnil (Azaperone 2 mg/kg; Elanco Animal Health, UK) injected intramuscularly in the neck and monitored over a 10–15 min period until appropriately sedated (immobile, absence of reaction to noise and tactile stimulation). Once sedated, the ear vein was catheterized and the animal was humanely killed by injection of Euthatal (sodium pentobarbitone 150 mg/kg i.v.; Abbott Laboratories, Illinois, USA). Once respiratory arrest and loss of corneal reflex was confirmed, the pig was transferred a short distance (~5 m) to a post-mortem room. Animals were exsanguinated and the cadavers were plunged into a cold water bath for 5 min to arrest post-mortem metabolism.

### Experimental Procedures (Tissue)

#### Tissue Dissection

Immediately following exsanguination and cold water immersion, the pigs were rapidly dissected to collect the caudal spinal cord and associated DRG. Once obtained, the ensheathed spinal cord and DRG were transferred into a Petri dish containing ice-cold sterile RNase/DNase free phosphate buffered saline solution to chill and wash off any blood contamination. The spinal cord and DRG were further dissected under a stereo microscope to remove the meningeal membranes to obtain the four caudal DRG corresponding to the four caudal nerves and most distal part of the caudal spinal cord receiving inputs from the DRG. All dissection equipment was carefully washed with RNaseZap (Sigma-Aldrich, UK) before and after use. Immediately following final dissection all tissues were placed into sterile RNase/DNase-free 2 ml cryotubes and snap frozen in liquid nitrogen. All samples were stored at −80°C prior to RNA extraction.

#### Tissue RNA Extractions

DRG weights were determined prior to total RNA extraction and tissues (~30 mg) were pooled placed into 2 ml bead homogenization tubes (Lysing matrix D—MP Biomedicals, UK) with QIAzol reagent (1 ml). DRG tissues were homogenized using a FastPrep FP120 cell disrupter (Qbiogene Inc., France) for 3 ×40 s, replacing the tubes back on ice for ~1 min in between homogenization bouts to prevent frictional heat build-up and possible RNA degradation. The resulting homogenate was used to prepare total RNA using the RNeasy Minikit (Qiagen, UK) according to the manufacturer's instructions (Qiagen RNeasy Mini Kit handbook 6/2012). Preparation of RNA using this spin column purification method included a DNase digestion step to eliminate possible genomic DNA contamination. RNA concentration and purity were determined by optical density (A_260_/A_280_ and A_260_/A_230_ ratios) using a NanoDrop 1000 spectrophotometer (Thermo Scientific, USA). RNA integrity was further evaluated using the RNA Screen tape assay (Agilent 2200 TapeStation, Agilent technologies, Germany). An RNA integrity number (RIN) for each sample was computed from a generated electropherogram and software. All samples exhibited intact *28S* and *18S* rRNA subunits with RIN ≥ 7.

#### Microarray Hybridization and Labeling

RNA labeling and hybridization were carried out by Edinburgh Genomics, University of Edinburgh (https://genomics.ed.ac.uk/). Total RNA (500 ng) was labeled using the WT Expression Kit (P/N 4411974; Invitrogen, UK) to produce single stranded cDNA which was labeled using the Affymetrix Terminal Labeling Kit (P/N 900652; Affymetrix UK Ltd, UK). Approximately 3 μg of fragmented, biotin-labeled cDNA was hybridized to the Affymetrix Porcine Gene 1.1 ST 96-Array Plate^1^ (394,580 probes; 22 median probes/gene; 19,212, gene-level probe sets) in the GeneTitan Multi-channel (MC) instrument using the Affymetrix standard protocols.

#### Data QC and Normalization

The .CEL files were loaded into Affymetrix Expression Console Software for initial QC and to check for any outlier samples prior to and after log_2_ robust multiarray averaging (RMA) normalization of probe set intensity values.

### Statistical Analysis

For differential expression analysis, the .CEL files were loaded into Partek Genomics Suite (Partek Inc., USA) using the standard gene expression workflow. The attributes for the individual data files were identified and statistical comparisons of gene expression were examined using a three-way analysis of variance (ANOVA) incorporating tail treatment (sham vs. amputated), time after treatment (1, 8, and 16 weeks), treatment age (3 vs. 63 days). The results from the 3-way ANOVA were subsequently filtered to identify genes showing differential expression in the contrasts set up (tail amputated vs. sham, controls). Only those genes achieving a false discovery rate (FDR) cut-off of 0.05 were reported in the filtered gene lists. Contrast data are presented as fold-change in transcript expression. A 3-way ANOVA was also performed on probe-set signal intensity data from selected genes derived from gene list enrichment analysis on functional clustering of genes associated with inflammatory and neuropathic pain. Data were analyzed using SAS software version 9.4 for windows (SAS Institute Inc., USA).

### Network Co-expression Analysis

A Pearson correlation matrix was constructed using the network analysis program Graphia Professional (Kajeka Ltd, Edinburgh, UK), formally known as BioLayout *Express*^3D^ (27), by comparing the expression profile of each probe set with an average expression >20 across all samples, to all others. A network graph was constructed using a threshold value of *r* ≥ 0.75, where nodes represent transcripts and the edges correlations between transcripts above the threshold. The graph was then clustered into co-expression clusters using the Markov clustering algorithm (MCL) (28) using an inflation value of 2.2, which determines the granularity of clustering. The profile of gene clusters was inspected manually to find clusters whose expression was associated with the experiment's design and the biology of the caudal DRG.

### Functional Enrichment Analysis

The functional associations of gene clusters were examined through Gene Ontology (GO) enrichment analysis using ToppGene (29) (https://toppgene.cchmc.org/). GO terms or pathways with a *p* < 0.05 were considered to be significantly enriched.

## Results

### Direct Link to Deposited Data

All .CEL files and normalized expression data files can be accessed through the NCBI Gene Expression Omnibus (GEO) international public repository (https://www.ncbi.nlm.nih.gov/geo/GSE119519).

### Differential Gene Expression

Significant differential gene expression (up and down) was observed following tail amputation in both 3 and 63 day-old pigs compared to respective sham treatment pigs (RMA 3-way-ANOVA; *p* < 0.05; FDR = 0.05) in all of the time points after tail treatment (i.e., still evident 16 weeks) after tail amputation injury. Expression patterns and the total number of differentially expressed transcripts varied within and between treatment age and over time. Table 1 shows the number of differentially expressed caudal DRG genes (up- and down-regulated) in a comparison between tail amputated and sham treatment pigs (range 119–3,794 genes). The total number of differentially expressed genes varied between the two different tail treatment age groups and with time after tail treatment within treatment age groups. The number of up- and down-regulated genes differed within tail treatment age and with time after tail treatment. The greatest number of genes exhibiting differential expression (up and down) was observed 8 weeks after tail amputation in both tail treatment age groups. Higher overall numbers of differentially expressed DRG genes were observed in the 63 day tail-amputated pigs compared to the 3 day-old treated pigs. An Excel spreadsheet containing log_2_ fold change data and for all the differentially expressed genes with RMA 3-way ANOVA *p* values for main factor effects and 3-way interactions can be found in Supplementary Data File 1.

**Table 1:** Total number of significant (*P* < 0.05) differentially expressed genes (up and down regulated) in caudal DRG neurons using an RMA 3-way-ANOVA contrast on tail amputated vs. sham-treated pigs undergoing tail treatment at 3 (neonatal) and 63 (juvenile) days of age (1, 8, and 16 weeks after tail treatment) with false discovery rate (FDR) = 0.05 applied (8^*^ pigs/treatment/age/time).

**Tail amputated vs. sham treatment**				
**Treatment** ** age**	**Time after treatment**	**Differentially expressed genes** ** (FDR = 0.05)**	**Up** ** regulated**	**Down** ** regulated**
3 days	1 week	518	351	167
	8 weeks	2,268	788	1,480
	16 weeks	119	89	30
63 days	1 week	1,530	918	612
	8 weeks	3,794	1,937	1,857
	16 weeks	780	569	211

* *Except for 63 day sham-treated T+1 week where n = 7 due to the removal of a tail bitten pig*.

### Network Co-expression Analysis

A gene co-expression graph was generated where a Pearson correlation threshold (*r* ≥ 0.75) was used to define relationships between genes. The graph's topology was highly structured and comprised of 6,459 nodes (transcripts) and 232,125 edges (correlations between expression profiles above the threshold). Localized areas of high connectivity, representing groups of genes with similar expression profiles, were defined using the MCL graph clustering algorithm. Gene clusters represented the different patterns of expression found within the data due to the variability of treatment groupings, different ages of the animals and sampling. Histograms of mean expression level of all genes within the 16 largest clusters (C1–C16) containing >50 transcripts are shown in Supplementary Figure 1.

**Figure 1:**
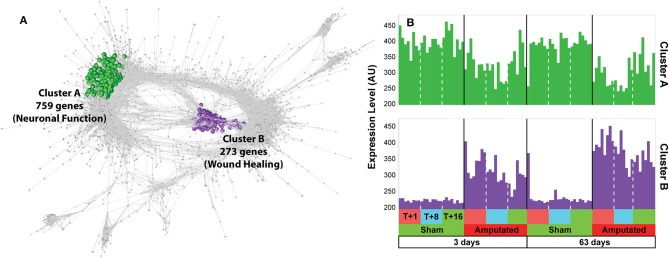
Network analysis of gene expression data. **(A)** Co-expression graph (*r* ≥ 0.75) with two clusters, A and B, highlighted. Cluster A (highlighted in green) contains 759 transcripts whose expression was down-regulated following tail docking and comprising many genes associated with neuronal function. Cluster B (highlighted in purple) contains 273 transcripts consistently upregulated following tail docking and comprising many genes associated with wound healing. **(B)** Histograms of mean expression profiles for cluster A (green) and cluster B (purple), each bar representing data from a single animal.

Following inspection of the co-expression network, only two clusters (A and B) we found to contain transcripts whose expression was consistently down or up regulated in response to treatment in both treatment age groups and across all three time points after tail amputation (Figure 1). Cluster A (1), green nodes, contains 759 transcripts whose expression was down-regulated following tail docking and comprises many genes associated with neurogenesis and neuronal function. Cluster B (2), purple nodes, contains 273 transcripts upregulated following tail docking and comprising many genes associated with response to wound healing. All of the genes identified within both clusters were consistently down- or up-regulated respectively, across all time points after tail amputation and in both tail treatment age groups. Log_2_ fold change gene expression values (tail amputation vs. sham controls) in both treatment age groups and at each time point after, and 3-way ANOVA *p* values for all Cluster A (highlighted green) and Cluster B (highlighted purple) can be found in Supplementary Data File 1.

### Gene Ontology (GO) and Pathway Enrichment Analysis

For each cluster the three most statistically enriched gene ontologies (Biological Process) are shown in Supplementary Figure 1, as analyzed using ToppGene. Of the 16 largest clusters, clusters 6 and 10 contained mainly unannotated genes for which no GO enrichment was obtained. This high level analysis suggests that other gene clusters in the data are associated with other variables, e.g., sampling of ependymal cells (cluster 3), oxidative phosphorylation (cluster 4), extracellular matrix (cluster 5), mitotic division, lower at later time points (cluster 9), interferon signaling (cluster 11), and macrophages (cluster 15).

**Figure 2:**
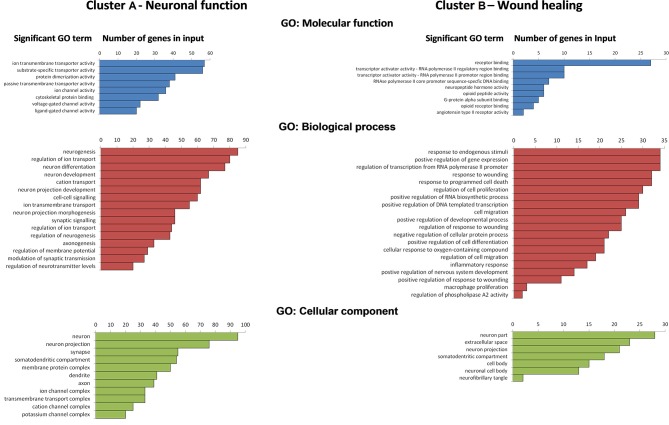
Ranks of significant gene ontology (GO) terms (*p* < 0.05 with Bonferroni correction) with number of genes in input within Clusters A and B for GO: molecular function, GO: biological process, and GO: cellular component derived from gene list enrichment analysis using ToppGene analysis suite.

Enrichment analysis of genes in Cluster A and Cluster B are shown for both clusters in Figure 2. The results for Cluster A show a significant enrichment of molecular function GO terms for primarily associated with *passive* and *voltage- gated ion transporter activity, ligand-gated ion transporter activity, substrate-specific transporter activity*, and *cytoskeletal protein binding*. Cluster A genes GO biological process terms included *neurogenesis, regulation of neurogenesis, neuron differentiation and development, axonogenesis, cell-to cell and synaptic signaling, regulation of membrane potential*, and *neurotransmitter levels* as well as *modulation of synaptic transmission*. Genes enriched in the GO cellular component analysis in Cluster A were associated specifically with *neuron* and *neuron components* (e.g., axons, dendrites, synapses) and *membrane protein transporter and receptor complexes*.

GO enrichment analysis for Cluster B genes showed significant enrichment of molecular function terms associated with *receptor binding, protein transcription activity and regulation, G-protein alpha subunit binding*, and *neuropeptide hormone activity*. Enriched functional gene groupings identified in Cluster B included those linked to GO biological processes such as *regulation of RNA biosynthesis, response to wounding*, and *regulation of response to wounding, inflammatory response, activation of the immune response* and *cell migration, cell differentiation, regulation of cell migration and differentiation*, and well as the *regulation of neuronal development and survival*. Genes revealed in the cellular component following GO enrichment analysis were associated primarily with *neuronal cells* and *neuron projections* and the *somatodendritic compartment*, as well as some *extracellular localization* in other cell types, e.g., macrophages.

### DRG Genes Linked to Inflammatory and Neuropathic Pain

As we have previously reported, tail amputation is associated with sustained, reduced nociceptive mechanical thresholds (7) and the development of traumatic neuromas (4) in the distal tail stump. Both of these phenomena can be linked to inflammatory or possible neuropathic pain. Therefore, we carried out a further level of analysis on the up and down-regulated genes identified in Clusters A and B. Gene lists for the GO terms “inflammatory pain” and “neuropathic pain” were generated from the GeneCards® integrated database of human genes (http://www.genecards.org/) for cross-reference with the porcine microarray gene chip library and network analysis cluster lists (see Supplementary Data Files 2, 3). GeneCards database generated an inflammatory pain list containing 2,630 genes and a neuropathic pain list consisting of 477 genes (correct to April 2018). All of the genes identified in the network clusters that were found in the inflammatory pain and neuropathic pain lists were verified by cross-reference with peer-reviewed published articles on peripheral nerve injury in the National Center for Biotechnology Information (NCBI) PubMed records (https://www.ncbi.nlm.nih.gov/pubmed/).

In Cluster A, a total of 124 pain-related genes were identified from the two pain genes lists. Within this Cluster, 81 genes were associated with inflammatory pain pathways and 16 genes exclusively linked to neuropathic pain pathways, with 27 of the expressed genes common to both pain states (Table 2). In cluster B, 61 genes were identified in total from both pain gene lists. Within this cluster, 48 genes were solely linked to inflammatory pain with 12 genes common to both pain pathways and only a single gene associated with neuropathic pain signaling. These genes were then selected for closer examination.

**Table 2:** Inflammatory pain and neuropathic pain genes^*^ expressed in network Cluster A (neuronal function) and Cluster B (wound healing).

	**Significant differentially expressed genes (*****p*** **< 0.05)**			
**Network cluster**	**Inflammatory pain**	**Neuropathic pain**	**Common to both lists**	**Total**
Cluster A (759 genes)	81	16	27	124
Cluster B (273 genes)	48	1	12	61

* *Lists for inflammatory pain (2,630) and neuropathic pain (477) genes were generated from GeneCards® Human gene database (correct to April 2018) and confirmed by cross-reference against citations on peripheral nerve injury in the National Center for Biotechnology Information (NCBI) PubMed biomedical literature database*.

#### Cluster A (Neuronal Function): Inflammatory and Neuropathic Pain Genes

Within network Cluster A, ~16% of the significant differentially expressed DRG genes changing in response to tail amputation were linked to the processes of pain signal detection, conduction, transmission, and modulation as determined by GO gene set enrichment analysis. GO term analysis identified several common functional families of genes involved pain signaling pathways these included: ion channels, transporters and receptors such as *voltage-gated potassium channel*s and *potassium ion leak channels, voltage-activated sodium channels, voltage-dependent calcium channels, cation transporters, solute carrier membrane transporters, gamma-aminobutyric acid (GABA) receptors, metabotropic glutamate receptors, cholinergic, adenosine, purinergic receptors*, and *opioid receptors*.

**Figure 3:**
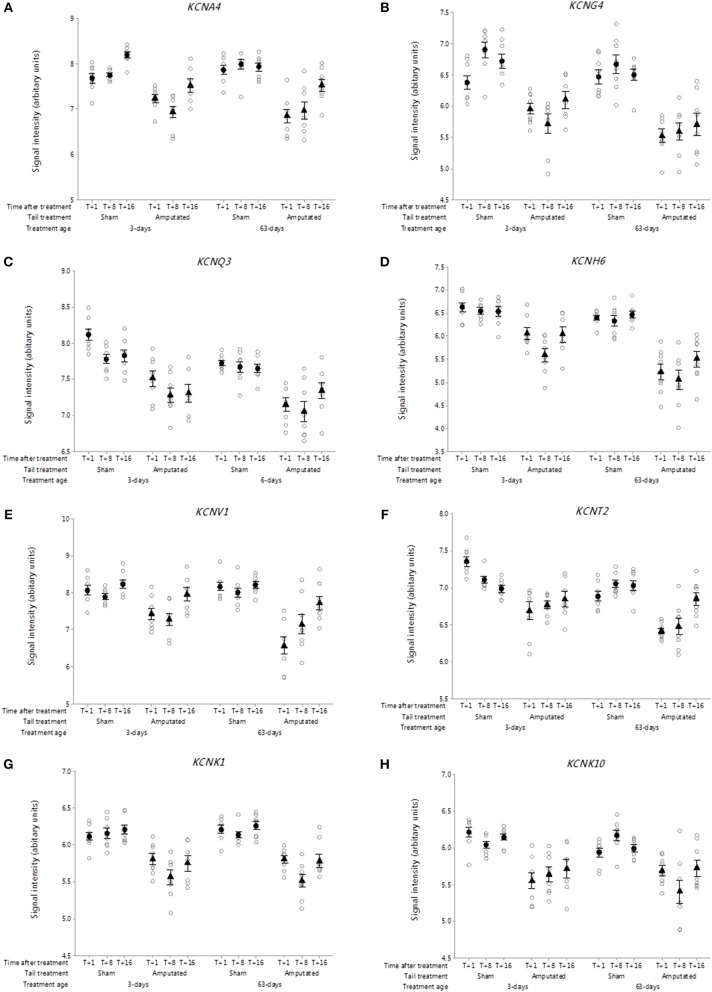
Probe-set signal intensities of selected significantly down-regulated DRG voltage-gated potassium channel genes after tail amputation in network Cluster A associated with inflammatory and neuropathic pain signaling. **(A)** *KCNA4*, Potassium voltage-gated channel subfamily A member 4; **(B)** *KCNG4*, Potassium voltage-gated channel modifier subfamily G Member 4; **(C)** *KCNQ3*, Potassium voltage-gated channel subfamily Q member 3; **(D)** *KCNH6*, Potassium voltage-gated channel subfamily H Member 6; **(E)** *KCNV1*, Potassium voltage-gated channel modifier subfamily V member 1; **(F)** *KCNT2*, Potassium sodium-activated channel subfamily T member 2; **(G)** *KCNK1*, Potassium two pore domain channel subfamily K member 1; **(H)** *KCNK10*, Potassium two pore domain channel subfamily K member 10. All signal intensity data values are shown by treatment group (open circles, *n* = 95) along with group mean ± SEM.

**Figure 4:**
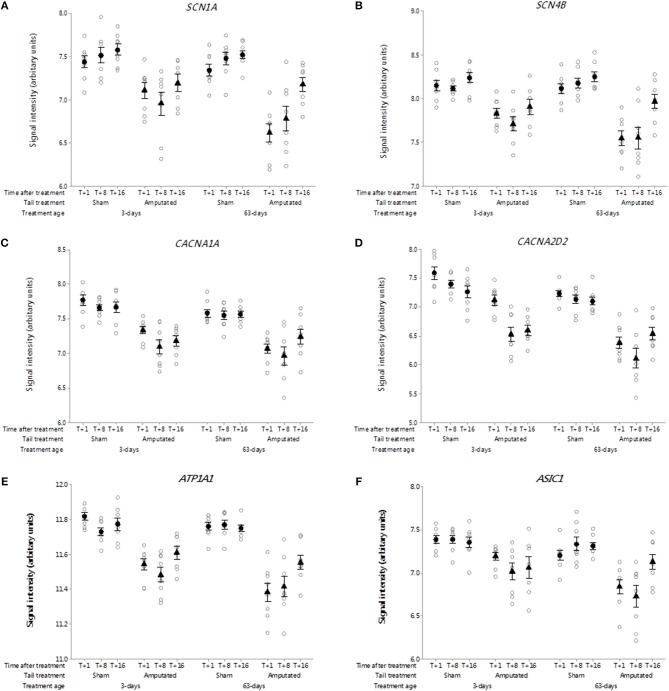
Probe-set signal intensities of selected significantly down-regulated DRG ion channel genes after tail amputation in network Cluster A associated with inflammatory and neuropathic pain signaling **(A)** *SCN1A*, Sodium voltage-gated channel alpha subunit 1; **(B)** *SCN4B*, Sodium voltage-gated channel beta subunit 4; **(C)** *CACNA1A*, Calcium voltage-gated channel subunit alpha 1A; **(D)** *CACNA2D2*, Calcium voltage-gated channel auxiliary subunit alpha 2 delta 2; **(E)** *ATP1A1*, ATPase Na^+^/K^+^ transporting subunit alpha 1; **(F)** *ASIC1*, Acid sensing (proton-gated) ion channel subunit 1. All signal intensity data values are shown by treatment group (open circles, *n* = 95) along with group means ± SEM.

**Figure 5:**
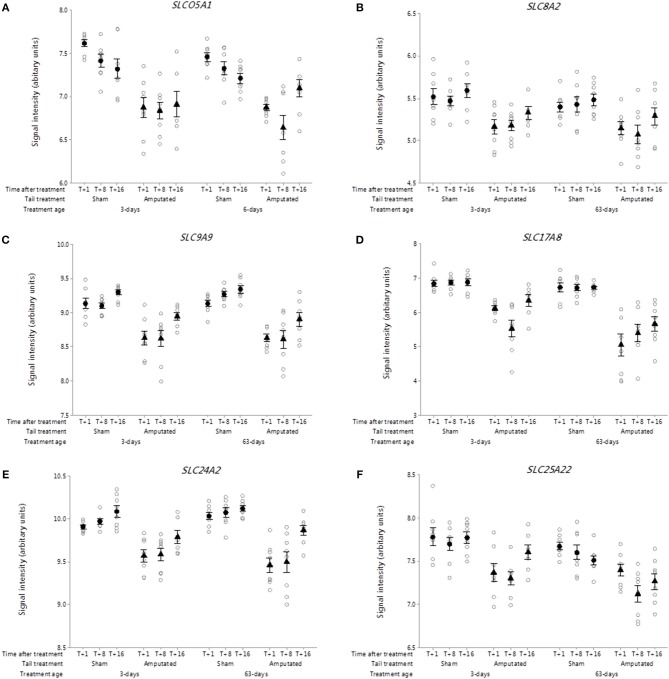
Probe-set signal intensities of selected solute carrier (SLC) sub-type DRG genes significantly down-regulated after tail amputation in network Cluster A associated with inflammatory and neuropathic pain signaling pathways. **(A)** *SLCO5A1*, Solute carrier organic anion transporter family 5 member A1; **(B)** *SLC8A2*, Solute carrier family 8 member A2 (sodium/calcium exchanger); **(C)** *SLC9A9*, Solute carrier family 9 Member A9 (sodium/proton exchanger); **(D)** *SLC17A8*, Solute carrier family 17 Member 8 (sodium-dependent inorganic phosphate co-transporter); **(E)** *SLC24A2*, Solute carrier family 24 Member A2 (sodium/potassium/calcium exchanger); **(F)** *SLC25A22*, Solute carrier family 25 Member 22 (mitochondrial carrier: glutamate). All signal intensity data values are shown by treatment group (open circles, *n* = 95) along with group means ± SEM.

**Figure 6:**
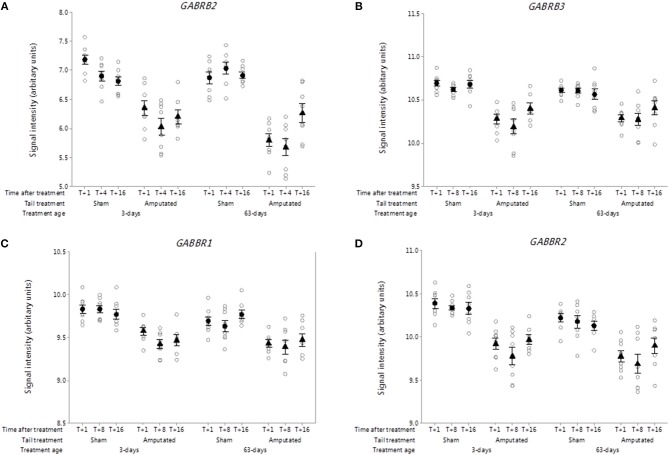
Probe-set signal intensities of significantly down-regulated DRG gamma-aminobutyric acid (GABA) receptor genes after tail amputation in network Cluster A associated with inflammatory and neuropathic pain signaling **(A)** *GABRB2*, Gamma-aminobutyric acid type A receptor beta 2 subunit; **(B)** *GABRB3*, Gamma-aminobutyric acid type A receptor beta 3 subunit; **(C)** *GABBR1*, Gamma-aminobutyric acid type B receptor subunit 1; **(D)** *GABBR2*, Gamma-aminobutyric acid type B receptor subunit 2. All signal intensity data values are shown by treatment group (open circles, *n* = 95) along with group means ± SEM.

**Figure 7:**
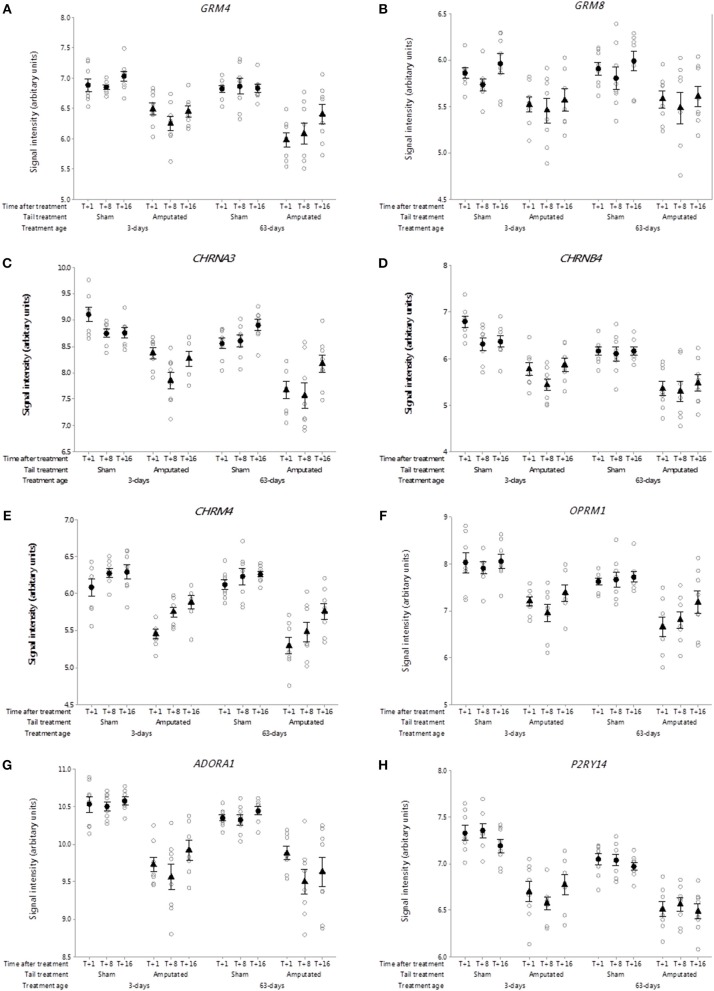
Probe-set signal intensities of selected significantly down-regulated DRG genes expressing G-protein-coupled neurotransmitter receptors after tail amputation in network cluster A associated with inflammatory and neuropathic pain signaling **(A)** *GRM4*, Glutamate metabotropic receptor 4; **(B)** *GRM8*, Glutamate metabotropic receptor 8; **(C)** *CHRNA3*, Cholinergic receptor nicotinic alpha 3 subunit; **(D)** *CHRNB4*, Cholinergic receptor nicotinic beta 4 subunit; **(E)** *CHRM4*, Cholinergic receptor muscarinic 4; **(F)** *OPRM1*, Glutamate metabotropic receptor 8; **(G)** *ADORA1*, Adenosine A_1_ receptor; **(H)** *P2RY14*, Purinergic receptor P2Y14. All signal intensity data values are shown by treatment group (open circles, *n* = 95) along with group means ± SEM.

DRG probe-set signal intensity plots for the genes illustrated in Table 3 from sham and tail amputated pigs shown over time and in both tail amputation age groups are presented in Figures 3–**7**. A three-way ANOVA table of all *p* values and F-ratios generated from a test of fixed effects with interactions for all of the highlighted Cluster A genes shown in Figures 3–**7** can be found in Supplementary Data 4.

**Table 3:** Functional groups, family member/subunit, and their associated significantly (3-way ANOVA; *p* < 0.05) down-regulated DRG pain genes^*^ after tail amputation in network Cluster A common to GeneCard inflammatory and neuropathic pain gene lists.

**Functional group**	**Member/subunit**	**Gene symbol**
Ion channel	Potassium (K^+^) channel subunit	*KCNA4, KCNG4, KCNQ3, KCNH6, KCNV1, KCNT2, KCNK1, KCNK10*
	Sodium (Na^+^) channel subunit	*SCN1A, SCN4B*
	Calcium (Ca^2+^) channel subunit	*CACNA1A, CACNA2D*
	Acid sensing proton (H^+^) channel	*ASIC*
	ATPase Na^+^/K^+^ subunit	*ATP1A1*
Solute transporter	Organic anion transporter family member	*SLCO5A1*
	Na^+^/Ca^2+^ exchanger family member	*SLC8A2*
	Na^+^/H^+^ exchanger family member	*SLC9A9*
Receptor	Gamma-aminobutyric acid (GABA) receptor subunit	*GABRB2, GABRB3, GABBR1, GABBR2*
	Metabotropic glutamate receptor	*GRM4, GRM8*
	Cholinergic receptor subunit	*CHRNA3, CHRNB4, CHRM4*
	Purinergic receptor	*P2RY14*
	Adenosine receptor	*ADORA1*
	Opioid receptor	*OPRM1*

* *Consistently down-regulated across all time points after tail amputation (T+1, T+8, and T+16 weeks) and common to both tail treatment age groups (3- and 63-days)*.

##### Voltage-gated potassium channels

The role of voltage-gated potassium channels (VGPC) in pain signaling is widely recognized (30). Forty mammalian VGPC genes have been discovered (31) of which 6 VGPC subfamily gene subunits have been linked to inflammatory and neuropathic pain. Twenty-six VGPC subunit genes were screened on the Affymetrix porcine gene array of which 8 subunit genes identified in Cluster A. Figures 3A–F show the probe-set intensity profiles of the 8 VGPC subunit genes identified in Cluster A, representing 6 channel sub-families (A-, G-, Q-, H-, V-, and T-type) exhibiting sustained reductions in transcript expression in response to tail amputation. Sustained and significant reductions in signal intensities were also observed in two K-type two-pore domain potassium leak channel genes (*KCNK1* and *KCNK10*) in response to tail amputation (Figures 3G,H). These leak channels contribute to passive transmembrane potassium transport and play a key role in regulation of neuronal excitability (32).

##### Voltage-gated sodium channels

Voltage-gated sodium-selective ion channels (VGSC) are present in the membrane of most excitable cells comprising a pore-forming α-subunit associated with either one or two β-subunits (33). The role of VGSC in neuropathic pain is still unclear as peripheral nerve injury down regulates most pain associated voltage channels in the injured DRG neurons (34). Genes coding for 9 α-subunits and 4 β-subunits have been identified in mammals. Probe-sets for 3 α-subunits and 2 β-subunits were present on the porcine microarray. Of these only *SCN1A* and *SCN4B* were identified in Cluster A (Figures 4A,B). Probe-set signal intensities for both channel subunit genes were significantly lower (*p* < 0.05) in response to tail treatment at both ages and over time compared to sham controls.

##### Voltage-gated calcium channels

Voltage-gated calcium channels (VGCC) are involved in the mediation of pain perception through the modulation of neurotransmitter release at presynaptic terminals of DRG sensory neurons (35). The channels are comprised of either 4 or 5 distinct subunits coded by multiple genes (36). To date 10 subunit genes have been identified in humans (37) of which probe-sets for 7 channel subunit genes were present on the pig array. Two subunit genes were present in network Cluster A of which *CACNA1A* and regulatory auxiliary subunit *CACNA2D2* are shown in Figures 4C,D. Probe-set signal intensities for both subunit genes were significantly lower (*p* < 0.05) in response to tail treatment at both ages, and over time compared to sham controls, consistent with other down-regulated genes in the Cluster A expression profile.

##### Other ion channels

ATPase Na^+^/K^+^ transporting subunit alpha 1 (*ATP1A1*). The protein encoded by this gene belongs to the family of P-type cation transport ATPases, and to the subfamily of Na^+^/K^+^ ATPases (38). Na^+^/K^+^ -ATPase is an integral membrane protein responsible for establishing and maintaining the electrochemical gradients of Na and K ions across the plasma membrane and have been implicated in mechanisms of neuropathic pain (23). Seven genes coding for 4 α- and 3 β-channel subunits have been characterized of which 6 subunits present were on the array. One subunit gene *ATP1A1* was present in network Cluster A (Figure 4E). Probe set signal intensities for this gene were markedly lower (*p* < 0.05) in response to tail treatment at both ages and over time compared to sham controls. Another down-regulated ion channel gene identified in Cluster A was acid sensing proton-gated ion channel subunit 1 (*ASIC1*). Acid-sensing ion channels are neuronal voltage-insensitive cation channels activated by extracellular protons (H^+^) and are involved in neurotransmission in inflammation and neuropathic pain conditions (39). Five subunit genes have been identified for this family of channels. *ASIC1* was the only one of the subunit genes found on the array and in common with all of the other ion channels co-expressed in Cluster A exhibited significantly lower (*p* < 0.05) microarray probe set signal intensities in response to tail treatment at both ages and over time compared to sham equivalents (Figure 4F).

##### Solute carrier membrane transporters

The solute carrier (SLC) group of membrane transport proteins include over 400 members organized into over 60 families (40). Recent research has highlighted solute carrier transporters as emerging therapeutic targets in inflammatory diseases such as inflammatory bowel disease (41). Over 170 SLC genes were on the porcine array of which 18 with sustained and significant reductions (*p* < 0.05) in gene probe set intensities were identified in Cluster A. Six of these genes (*SLCO5A1, SLC8A2, SLC9A9, SLC17A8, SLC24A2, SLC25A22*) are highlighted in Figures 5A–F, representing key SLC subfamily members associated with anionic, cationic, excitatory amino acid and mitochondrial ADP/ATP transport.

##### Gamma aminobutyric acid (GABA) receptors

The roles of the inhibitory neurotransmitter gamma amino butyric acid (GABA) and its receptors in nociceptive signal processing and pain are well-recognized (42). Nineteen ionotropic type A (GABA AR) and two metabotropic type B (GABA BR) receptor subunit genes have been identified of which 7 type A and both type B receptors probe sets were present on the microarray. Two ionotropic (*GABRB2, GABRB3*) and both metabotropic (*GABBR1, GABBR2*) receptor subunits were significantly down-regulated in Cluster A (Figures 6A–D). Probe-set intensity values for all of the DRG GABA-R subunit genes were significantly lower (*p* < 0.05) in response to tail treatment at both ages and over time compared to sham controls.

##### Metabotropic glutamate receptors

Glutamate is a major amino acid that functions as an excitatory neurotransmitter in pain pathways (43). Eight genes for metabotropic G-protein coupled glutamate receptors have been determined in mammals of which 4 had probe sets on the microarray. In Cluster A, the expression of two metabotropic glutamate receptor genes (*GRM4* and *GRM8*) was significantly down-regulated (*p* < 0.05) as a consequence of tail amputation (Figures 7A,B). The down-regulation in gene expression reflected by the sustained reduction in probe set intensity values was still evident ca. 4 months after tail treatment at 3 and 63 days of age in comparison to the shams.

##### Nicotinic and muscarinic acetylcholine receptors

Nicotinic acetylcholine receptors (nAChRs) are cholinergic receptors that form ligand-gated ion channels in pre- and postsynaptic neural membranes that are produced by 16 genes that code for 5 receptor subunits (α, β, δ, ε, γ) (44). In the present study probe sets for 10 genes were present on the array of which two neuronal specific genes (*CHRNA3* and *CHRNB4*) were observed in the Cluster A co-expression network. Probe-set intensity values for both nAChR subunit genes were significantly lower (*p* < 0.05) in response to tail amputation treatment at both ages and over time compared to sham controls (Figures 7C,D). In addition a single muscarinic acetylcholine receptor gene (*CHRM4*) was also found to be down regulated in the DRG Cluster A as a consequence of tail amputation (Figure 7E). Muscarinic acetylcholine receptors (mAChRs) are receptors that form G protein-receptor complexes in the cell membranes of neurons and possess a regulatory effect on dopaminergic neurotransmission (45). Five genes have been identified of which only probe-sets for *CHRM4* were present on the array.

##### Opioid receptors

It is well-recognized that exogenous and endogenous opioids and their opioid receptors play a major role in pain relief (46, 47). Three genes coding for 3 opioid receptors (mu, delta, kappa) have been identified in mammals (48). Probe sets for two of those genes *OPRM1* and *OPRK1* coding for opioid receptors mu and kappa, respectively, were present on the array. Of these only *OPRM1* was present in network Cluster A (Figure 7F). Probe-set intensity values for *OPRM1* were significantly and persistently lower (*p* < 0.05) in response to tail amputation at both ages and over time compared to sham controls.

##### Adenosine receptors

The antinociceptive actions of adenosine and it's receptors on pain modulation is widely recognized (49). Four genes coding for adenosine receptors have characterized in mammals. Probe sets for 3 of those genes were present on the gene array. Only one of the genes *ADORA1* occurred in Cluster 1 (Figure 7G) and in common with all of the other Cluster A DRG genes exhibited a significant reduction (*p* < 0.05) in probe set signal intensity as a consequence of tail amputation that was sustained up to 4 months in both treatment age groups.

##### P2Y purinergic receptors

Metabotropic P2Y receptors play a crucial role in facilitating pain transmission at peripheral and spinal sites (50). There are eight known genes coding for P2Y receptor. In the current study, there were probe sets for 6 of the P2Y receptor genes of which *P2YR14* was identified in network Cluster A (Figure 7H). DRG *P2YR14* gene signal intensities were significantly lower in the tail amputated animal compared to controls in both treatment age groups. Lowered signal intensities were still evident at 16 weeks after tail treatment (*p* < 0.05).

#### Cluster B (Wound Healing): Inflammatory and Neuropathic Pain Genes

Within network Cluster B the majority of significant, differentially expressed DRG genes changing in response to tail amputation treatment and linked to inflammatory and neuropathic pain pathways were common to several GO term functional gene families associated with *transcription factor activity, DNA binding transcription factor activity, regulation of DNA binding transcription factor activity, polymerase II transcription factor activity, sequence-specific DNA binding, transcription from RNA polymerase II promoter, protein homodimerization activity, immune response, immune cell proliferation and migration inflammatory response, regulation of the inflammatory response, wound healing, peptidase inhibitor activity, protein tyrosine kinase activity, activation of adenylate cyclase activity, receptor binding, signal transducer activity, neurotrophin signaling pathway, neuropeptide signaling pathway, neuronal survival, neuron development and migration, potassium channel activity*.

**Figure 8:**
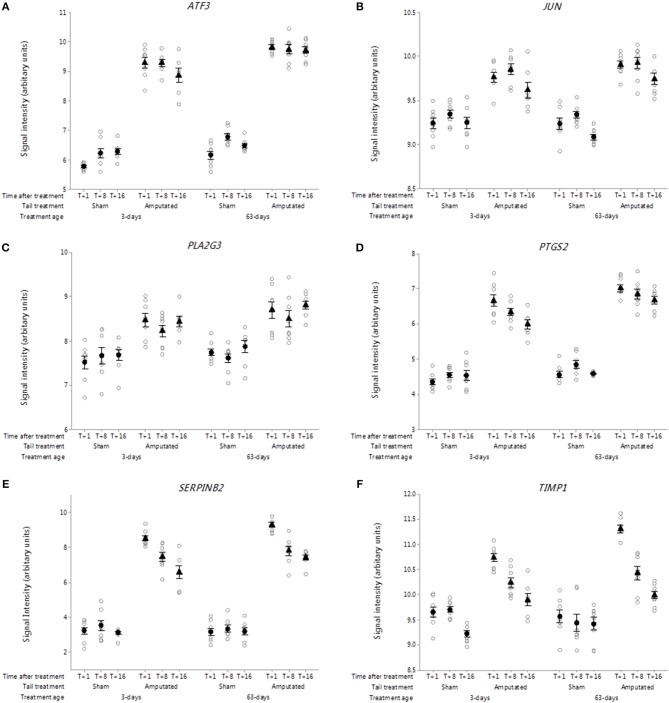
Probe-set signal intensities of DRG genes in network Cluster B involved in inflammatory and neuropathic pain pathways expressing significant up-regulated transcript activity (3-way ANOVA, *p* < 0.05) 1, 8, and 16 weeks after tail amputation at two ages (3 and 63 days). Genes shown are linked to regulation of gene expression, inflammation, proteinase inhibition, and neurotropic signaling. **(A)** *ATF3*, Activating transcription factor 3; **(B)** *JUN*, Jun proto-oncogene, AP-1 transcription factor subunit; **(C)** *PLA2G3*, Phospholipase A2 group III; **(D)** *PTGS2*, Prostaglandin-endoperoxide synthase 2; **(E)** *SERPINB2*, Serpin family B member 2; **(F)** *TIMP1*, TIMP metallopeptidase inhibitor 1. All signal intensity data values are shown by treatment group (open circles, *n* = 95) along with group means ± SEM.

**Figure 9:**
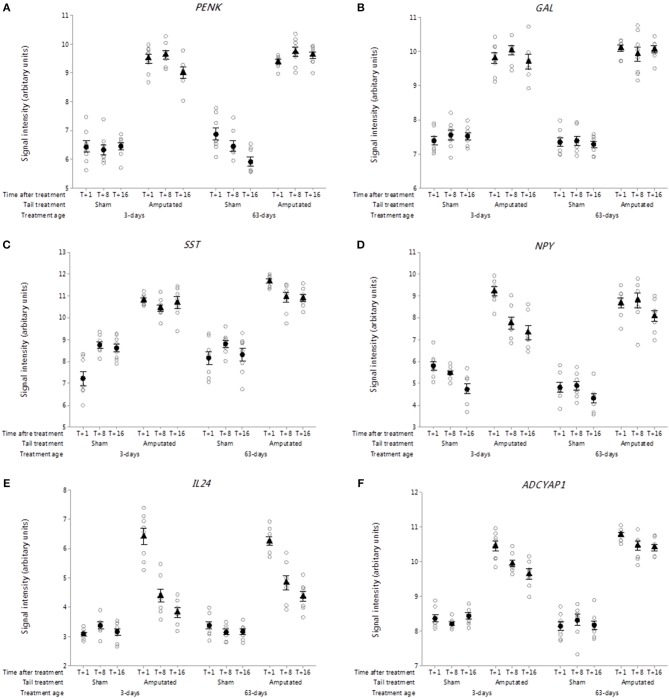
Probe-set signal intensities of selected up-regulated DRG genes in response to tail amputation in network Cluster B encoding for endogenous neuroactive peptides involved in the modulation of inflammatory and neuropathic pain signaling. **(A)** *PENK*, Proenkephalin; **(B)** *GAL*, Galanin and GMAP Prepropeptide; **(C)** *SST*: Somatostatin; **(D)** *NPY*, Neuropeptide Y; **(E)** *IL24*, Interleukin-24; **(F)** *ADCYAP1*, Adenylate cyclase activating peptide 1. All signal intensity data values are shown by treatment group (open circles, *n* = 95) along with group means ± SEM.

**Figure 10:**
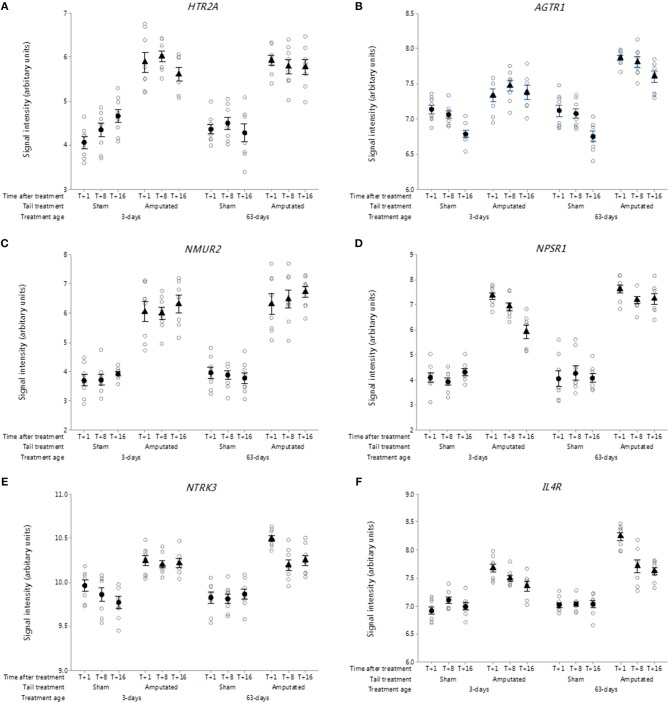
Probe-set signal intensities of selected significantly up-regulated DRG genes in network Cluster B encoding for G-protein coupled and interleukin receptors involved in the mediation of inflammatory and neuropathic pain. **(A)** *HTR2A*, 5-Hydroxytryptamine receptor 2A; **(B)** *AGTR1*, Angiotensin II receptor type 1; **(C)** *NMUR2*, Neuromedin U receptor 2; **(D)** *NPSR1*, Neuropeptide S receptor 1; **(E)** *NTRK3*, Receptor tyrosine kinase 3; **(F)** *IL4R*, Interleukin 4 receptor. All signal intensity data values are shown by treatment group (open circles, *n* = 95) along with group means ± SEM.

**Figure 11:**
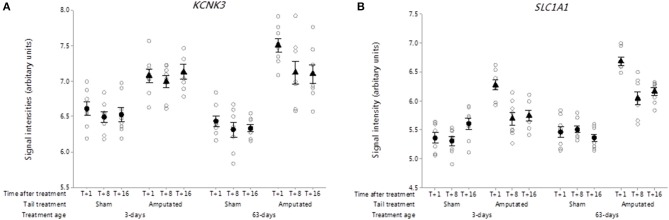
Probe-set signal intensities of significantly up-regulated DRG genes in network Cluster B in response to tail amputation involved in the mediation of inflammatory and neuropathic pain signaling. **(A)** *KCNK3*, Potassium two pore domain channel subfamily K member 3 and neuronal high affinity glutamate transporter; **(B)** *SLC1A1*, Solute carrier family 1 member 1. All signal intensity data values are shown by treatment group (open circles, *n* = 95) along with group means ± SEM.

Pig DRG pain genes identified in Cluster B that were consistently up-regulated at all three time points (1, 8, and 16 weeks) after tail amputation and common to both tail amputation groups (3-days and 63-days) are shown in Table 4. DRG probe-set signal intensity plots for the genes illustrated in Table 3 from sham and tail amputated pigs shown over time and in both tail amputation age groups are presented in Figures 8–11. A three-way ANOVA table of all *p* values and F-ratios generated from a test of fixed effects with interactions for all of the highlighted Cluster B genes shown in Figures 8–11 can be found in Supplementary Data 5.

**Table 4:** Functional groups, subgroups, and their associated significantly (3-way ANOVA; *p* < 0.05) up-regulated DRG pain genes^*^ after tail amputation in network Cluster B common to GeneCard inflammatory and neuropathic pain gene lists.

**Functional group**	**Member**	**Gene symbol**
Transcription factor	Activating transcription factor 3	*ATF3*
	Jun proto oncogene	*JUN*
Neuropeptide	Proenkephalin	*PENK*
	Somatostatin	*SST*
	Galanin/GMAP propeptide	*GAL*
	Neuropeptide Y	*NPY*
	Adenylate cyclase activating polypeptide	*ADCYAP1*
Inflammatory intermediate	Phospholipase A2 group III	*PLA2G3*
	Prostaglandin-endoperoxide synthase 2	*PTGS2*
	Interleukin 24	*IL24*
Receptor	5-Hydroxytryptamine (serotonin) receptor	*HTR2A*
	Angiotensin II receptor	*AGTR2*
	Neuromedin U receptor	*NMUR2*
	Neuropeptide S receptor	*NPSR*
	Neurotrophin receptor tyrosine kinase	*NTRK3*
	Interleukin 4 receptor	*IL4R*

* *Consistently up-regulated across all time points after tail amputation (T+1, T+8, and T+16 weeks) and common to both tail treatment age groups (3- and 63-days)*.

##### Transcription factors

DRG probe-set signal intensities of activating transcription factor 3 (*ATF3;* Figure 8A) and Jun proto-oncogene, AP-1 transcription factor subunit (*JUN;* Figure 8B) were significantly up regulated (*p* < 0.05) in Cluster B and remained increased up to 16 weeks after tail injury in both treatment age groups compared to sham-treated controls. Both transcription factors are associated with inflammatory and neuropathic pain conditions linked to nerve injury (51, 52).

##### Arachidonic acid metabolism intermediates

Arachidonic acid is a key inflammatory intermediate and its conversion into a wide range of clinically important bioactive compounds (eicosanoids) involved in the generation and maintenance of inflammatory pain is well-documented (53, 54). Within the DRG Cluster B network, signal intensities for the phospholipase A_2_ group III (*PLA2G3*) a gene that encodes for secreted phospholipase A_2_ enzyme which catalyses the hydrolysis of phospholipids to release of arachidonic acid and lysophospholipids was significantly higher (*p* < 0.05) in the tail amputated vs. sham treated controls in both treatment age groups (Figure 8C). Similarly, probe-set signal intensities for prostaglandin-endoperoxidase synthase 2 (*PTGS2*), were significantly higher (*p* < 0.001) in both tail amputated groups. Again, these increases were sustained up to 16 weeks after tail injury (Figure 8D). *PTGS2* codes for the enzyme prostaglandin-endoperoxidase synthase which is formally known as cyclooxygenase 2 (COX-2) a key tissue-injury inducible enzyme in prostanoid biosynthesis involved in inflammation and mitogenesis (55).

##### Peptidase inhibitors

Specific inhibitors of neuronal proteases known as serpins are postulated to play a key role in neuronal survival, synaptic plasticity and axonal development, regeneration, and the amelioration of neuropathic pain (56, 57). In this study within the DRG genes identified in network Cluster 6, signal intensities for the Serpin family B member 2 (*SERPINB2;* Figure 8E) were highly and significantly elevated in both tail treatment age groups compared to sham controls (*p* < 0.001). Signal intensities of tissue inhibitor of metalloproteinases 1 (*TIMP1*, Figure 8F) were also significantly increased in tail amputated vs. sham control DRGs in both tail treatment ages (*p* < 0.05). A significant time-related reduction in signal intensity expression at 16 weeks was observed in both tail amputation group compared to T+1 week (*p* < 0.05). Transcription of this gene is highly inducible in response to peripheral nerve injury (58) and it has been suggested that TIMP1 and other TIMP proteins may have therapeutic potential for the treatment of neuropathic pain (59).

##### Endogenous neuropeptides

Probe set intensities for genes of several key endogenous neuroactive peptide ligands involved in pain signal modulation in DRG neurons were significantly increased (*p* < 0.001) after tail amputation compared to sham controls in both tail treatment age groups (Figures 9A–D). These included genes coding for proenkephalin (*PENK*, Figure 9A), galanin and GMAP prepropeptide (*GAL*; Figure 9B), somatostatin (*SST*; Figure 9C) neuropeptide Y (*NPY*; Figure 9D), Interleukin-24 (*IL24*; Figure 9E), and adenylate cyclase activating peptide 1 (*ADCYAP1*; Figure 9F).

The *PENK* gene encodes for a preproprotein that is proteolytically processed to generate multiple neuropeptide products that include the opioids Met-enkephalin and Leu-enkephalin, which are stored in synaptic vesicles and released into the synapse where they bind to mu- and delta-opioid receptors to modulate the perception of pain (60). *PENK* mRNA and its post-translational products have also been detected in immune cells and been shown to play a key role in the modulation of pain signaling in the peripheral sensory nervous system (61).

The *GAL* gene is widely expressed in peripheral and central nervous system and codes for a precursor protein that is proteolytically processed to generate two mature peptides: galanin and galanin message-associated peptide (GMAP). Galanin has diverse physiological functions including nociception, learning and memory (62, 63). GMAP mirrors galanin expression being derived from the same precursor protein and but unlike galanin may have a limited role in modulating nociceptive signals after nerve injury (64).

*SST* gene codes for a preprotein that is subsequently cleaved to produce the cyclic neuropeptide hormone somatostatin (formerly known as somatotropin release inhibiting factor). It is an abundant neuropeptide and has a wide range of physiological effects on neurotransmission, secretion, and cell proliferation (65). Somatostatin has been shown to have anti-nociceptive and anti-inflammatory effects and markedly inhibits cross-excitation exerted by the release of excitatory neurotransmitters such as glutamate and substance P (66, 67).

Within the Cluster B pain genes there was also observed a sustained and significant increase in probe-set signal intensities for the neuropeptide Y gene (*NPY*) in both tail amputated groups (*p* < 0.05). Neuropeptide Y (NPY) the neuropeptide hormone expressed in peripheral DRG neurons can have both anti- and pro-nociceptive effects depending upon its end target receptor location (68). These authors also suggest that NPY and galanin may work together in partnership in the modulation of neuropathic pain caused by severe peripheral nerve injury (69).

The role of pro- and anti-inflammatory cytokines has long been recognized in inflammatory and neuropathic pain conditions (70, 71). In the present study highly significant initial increases (T+1 week) in DRG probe set intensities for the gene coding for interleukin-24 (*IL24*) were observed (Figure 9E) in the two tail-amputated groups compared to their respective sham-handled controls (*p* < 0.05). This initial increase appeared to attenuate over time but still remained significantly elevated compared to controls at 8 and 16 weeks following tail resection injury. Interleukin-24 (IL-24) is a member of the IL-10 family of cytokines and is primarily produced in peripheral blood mononuclear cells but also found in keratinocytes, melanocytes, fibroblasts and other tissues. It plays a key role in inflammatory and antibacterial immune responses, tissue remodeling, wound healing, anti-tumor effects (72).

Adenylate cyclase activating peptide 1 *(ADCYAP1)* encodes the pro-protein pituitary adenylate cyclase-activating polypeptide (PACAP) a multifunctional neuropeptide belonging to the glucagon-secretin-vasoactive intestinal peptide (VIP) family (73). PACAP stimulates adenylate cyclase and increases cyclic adenosine monophosphate (cAMP) levels, resulting in the transcriptional activation of target genes. The products of this gene are recognized as key mediators of neuroendocrine stress responses (74). DRG probe set intensities for the *ADCYAP1* gene were significantly elevated (*p* < 0.05) and remained elevated up to 4 months after tail amputation treatment in both tail treatment age groups compared to sham handled pigs (Figure 9F). Axotomy induced increases in *ADCYAP1* gene expression have been previously reported facial motor nucleus neurons (75) and it has been suggested that the encoded PACAP protein may play a physiological role as a neurotransmitter or neuromodulator in sensory C-fiber pain signaling (76).

##### 5-hydroxytryptamine (serotonin) receptors

After nerve injury, the serotonin content of the lesioned nerve increases, concomitant with this is an increase in several neuronal 5-HT receptors (77). Thirteen 5-HT receptor sub-type genes have been identified of which 6 were present on the Affymetrix array. In this study, there were significant increases (*p* < 0.05) in DRG gene probe set intensities for 5-HT receptor 2A (*HTR2A*) in the tail amputated groups compared with shams in both tail treatment ages Figure 10A). Increased 5-HT2A receptor expression in DRG neurons has been linked to the potentiation of inflammatory pain signaling in the peripheral nervous system and may also be involved in the development of neuropathic pain (78). 5-HT acting in combination with other mediators can ectopically excite and sensitize afferent nerve fibers contributing to peripheral sensitization and hyperalgesia in inflammation and nerve injury (77).

##### Angiotensin II receptors

Angiotensin receptors are a class of G protein-coupled receptors with angiotensin II as their primary ligand. They are responsible for the signal transduction of the vasoconstriction stimulus of the main effector hormone, angiotensin II. There are two genes contained within this family angiotensin I receptor type I (*AGTR1*) and II (*AGTR2*). Probe sets for both genes were present on the microarray but only *AGTR1* was present in the network Cluster B pain genes list (Figure 10B). Signal intensities for *AGTR1* were significantly higher over the duration of the study in the two tail amputated groups at 3 and 63 days compared to sham controls (*p* < 0.05). *AGTR1* mRNA has previously been shown to be markedly elevated in response to peripheral nerve injury by crushing or axotomy (79) and its coded receptor, angiotensin II type 1 receptor (AT_1_) is believed to be involved in Schwann cell-mediated myelination and in neuroregenerative response in DRG neurons.

##### Neuromedin U receptors

The neuromedin U receptors are two G-protein coupled receptors which bind the neuropeptide hormone neuromedin U (80). There are two subtypes of the neuromedin U receptor, each encoded by a separate gene (*NMUR1, NMUR2*). Only *NMUR2* was present on the array chip and it was also subsequently identified in the Cluster B correlation network. *NMUR2* exhibited large and sustained increases in gene probe set signal intensities in the tail amputated groups (*p* < 0.05) compared with sham controls in the two tail treatment ages (Figure 10C). Neuromedin U (NMU) has been reported to be involved in the modulation of spinal nociceptive reflexes through its interaction with the NMU type 2 receptor in spinal dorsal horn neurons (81).

##### Neuropeptide S receptor

Neuropeptide S receptor (NPSR) belongs to the G-protein coupled receptors (GPCR) family and it binds neuropeptide S (NPS) which has several biological actions depending upon the cell type and location (82). In the central nervous system, the NPS-NPSR system promotes anxiolytic-like effects (83), whereas in spinal nervous systems and peripheral tissues it appears to be involved in the modulation of chemotaxis and inflammation (84, 85) and nociception (86). DRG neuropeptide S receptor 1 (*NPSR1*) gene probe-set signal intensities were significantly increased (*p* < 0.05) and remained increased up to 16 weeks after tail amputation in the two different tail treatment age groups compared to sham-handled controls, although signal intensities for *NPSR1* appeared to reduce over time in the neonatal tail amputated piglets compared to the juvenile tail amputated pigs (Figure 10D).

##### Neurotrophin receptors

Receptor tyrosine kinases (RTKs) represent a key family of cell-surface receptors, which transduce signals to neuropeptides, hormones, cytokines and growth factors, which in neurons are involved in numerous cellular processes including neuronal differentiation and proliferation, survival and metabolism, axonal guidance, and cell cycle control (87). Neurotrophic receptor tyrosine kinase 3 (*NTRK3;* Figure 10E) belongs to the neurotrophin subfamily of neurotrophic receptors and exhibited a significant increase in probe-set DRG signal intensity expression in tail amputated pigs compared with sham-handled controls which was sustained up to 16 weeks after tail treatment (*p* < 0.05). Neurotrophins are potent regulators of the survival of different neuronal populations in the peripheral and central nervous systems. Upon binding of its ligand neurotrophin-3 (NTF3), NTRK3 receptors auto-phosphorylate and activate different signaling pathways, including the phosphatidylinositol 3-kinase/AKT and the MAPK pathways that control cell survival and differentiation. NTF3 its cognate NTRK3 receptor have been shown to have antagonistic (anti-nociceptive) effects to NGF in pain processing in intact neurons (88, 89) and NTF3 can reduce an over expression of VGSCs Nav1.8 and Nav1.9 in the DRG neurons of neuropathic rats (90) and play a role in the modulation of neuropathic pain (91).

##### Interleukin receptors

Also identified within the Cluster B network of inflammatory and neuropathic pain genes was the gene coding for the alpha chain of the interleukin-4 receptor (*IL4R*: Figure 10F). DRG probe set signal intensities for *IL4R* were significantly elevated (*p* < 0.05) in response to tail amputation in both tail treatment age groups compared to sham amputation control animals. The *IL4R* gene codes for interleukin-4 (IL-4) alpha subunit and its is primarily expressed by T cells, natural killer T cells, mast cells and eosinophils, and initiates signal transduction through one of two different (type I and II) receptor complexes type 1 receptor is expressed on hematopoietic cells whereas type II receptors are expressed on non-hematopoietic cells (92). The type I receptor consists of the IL-4 R alpha and common gamma-chain/IL-2 R gamma subunits and is specific for IL-4, whereas the type II receptor consists of the IL-4 R alpha and IL-13 R alpha 1 subunits and can be activated by either IL-4 or IL-13. IL4 is an anti-inflammatory cytokine that exerts through its actions on IL4 receptor complexes that module macrophage activity by the suppression of pro-inflammatory cytokines (93, 94) and has been shown to ameliorate neuropathic pain at the spinal level (95).

##### Potassium leak channel (K2P)

In contrast to the 9 down-regulated potassium channels observed in Cluster A, a single K-type two-pore (K2P) domain potassium leak channel gene *KCNK3* was distinguished in Cluster B in response to tail amputation (Figure 11A). *KCNK3* codes for pH-dependent, voltage-insensitive, background potassium channel rectifier protein which acts as an outward rectifier when external potassium concentration is low and as an inward current rectifier when the extracellular potassium is high (96). It is well-established that K2P channels are regulators of the excitability of sensory neurons and therefore pain signaling (97) and regarded as potential therapeutic targets for the treatment if pain (32). In this study DRG probe-set signal intensities for *KCNK3* transcripts were significantly increased (*p* < 0.05) in the tail amputated pigs compared to the sham-handled controls in both tail treatment age cohorts and was maintained up to 16 weeks after tail amputation injury.

##### Excitatory amino acid transporter

Solute carrier family 1 member 1 *(SLC1A1)* gene codes for a high-affinity glutamate transporter, excitatory amino acid transporter 1 (EAAC1), that play an essential role in transporting glutamate across neural membranes (98). These transporters are crucial in terminating the postsynaptic action of the neurotransmitter glutamate, and in maintaining extracellular glutamate concentrations below neurotoxic levels. In the present study, DRG probe-set signal intensities for *SLC1A1* transcripts were significantly higher in both tail amputated treatment groups (*p* < 0.05) compared to their respective sham-handled controls (Figure 11B). Maximal increases in gene probe-set intensities in both tail treatment groups were observed 1 week after tail treatment and appeared to attenuate thereafter but still remain higher than controls. Studies on peripheral nerve injury in rats indicate that glutamate transporters such as EAAC1 may play a critical role in both the induction and maintenance of neuropathic pain induced by peripheral nerve injury (99).

## Discussion

Allied to the current study we have previously demonstrated that tail amputation in pigs is associated with significant caudal nerve injury (complete nerve transection) and subsequent development of traumatic neuromas in the caudal DRG neurons in the residual tail stump (4). In addition, this is associated with long-term hyperalgesia (lowered response thresholds to noxious mechanical stimulation) in the tail stump (7). The results from this transcriptomics study clearly show that tail amputation causes marked and sustained changes in the expression of ~3,000 genes. Characterizing which of these DRG genes are changing and how they alter over time after tail amputation is key to determining the likely long-term implications of these transcriptional changes for residual stump pain.

With the quantification of many thousands of genes on the Affymetrix porcine microarray (19,212 gene-level probe sets), it is important to define the optimal criteria for minimizing the false positive error rate (100). In the current study, a FDR of 0.05 was applied to reduce the risk of generating potential false positives to 5%. This cut off did not significantly impact on the loss of differentially expressed genes (sensitivity) and the sample size per group (*n* = 8) was appropriate for statistical comparison. Genes for tail amputated pigs exhibiting significant differences (*p* ≤ 0.05) compared to their respective treatment age and time-matched sham controls (up and down regulated) were subsequently investigated in the study. While, it is recognized that the quantification of gene expression in this study is limited to only those available on the Affymetrix chip, a large proportion of the genes involved in inflammatory and neuropathic pain signaling were present on the microarray and provide an acceptable level of screening of pain-associated genes.

To the best of our knowledge, there have been no other transcriptomics studies performed on porcine DRG neurons, despite the emergence of the pig as a model species for preclinical translational pain studies (101–104). Historically, pain-related transcriptomics studies on DRG neurons have been exclusively performed in rodents undergoing sciatic nerve crushing or transection (axotomy) with gene expression profiling conducted over a relatively short time (≤10 days) after nerve injury (21–24, 105). Comparisons of the gene lists generated in this study and those reported by others is not straightforward, not least due to the obvious species, neuroanatomical, and treatment differences but also by the application of different criteria used to identify the genes, i.e., different technology platforms. However, it is encouraging to observe that a large number of genes within several gene families showed a consistency in their response to peripheral nerve injury in this and other studies (21).

While, this study has shown changes in a number of genes implicated in inflammatory and neuropathic pain pathways, it must be recognized that peripheral nerve damage by axotomy does not normally lead to neuropathic pain in very young animals (106). Experimental spared nerve injury (SNI) which causes marked mechanical allodynia and hyperalgesia which are both key features of neuropathic pain in adult rodents, does not typically produce equivalent responses when performed in neonatal rat pups (107–109). However, if SNI is performed post-natal day 33 or later does it produces comparable neuropathic pain symptoms to those observed in older animals. Longitudinal studies have however demonstrated that animals that experience nerve injury early in life eventually develop pain hypersensitivity later in life (110). These finding suggest that neuropathic pain is not so much absent but suppressed during early development. These experimental findings are also consistent with clinical experience where neuropathic pain following nerve injury is rare, although some evidence of neuropathic pain in infants has been reported (111–114). The reason for these developmental differences in the manifestation of neuropathic pain is not fully understood. It has been suggested by Mckelvey et al. (115) that the long-term painful consequences of peripheral nerve injury in neonate animals/infants may go undetected due to anti-inflammatory neuroimmune suppression of dorsal horn neuronal excitation during the process of spinal somatosensory reorganization after nerve damage. It is postulated that as an affected animal ages, and it's CNS matures, it's neuroimmune system shifts in a more pro-inflammatory direction that unmasks latent pain responses to the nerve injury sustained early in life and that this can emerge as clinically unexplained or functional pain later in life (115). If the same processes occur in neonatal tail docked piglets it may have important long-term implications for pig health and welfare. If neuropathic pain is unmasked in tail docked pigs in later life in commercial production situations its effects on pig behavior may go largely unobserved and unreported, as focus on pain-related behavior in piglets is exclusively carried out at the time docking and a few days afterwards (1). In addition, if tail amputated piglets eventually develop pain hypersensitivity in the distal stump later in life the consequences of tail biting on pain severity may be exacerbated in older tail docked animals.

Tail amputation causes tissue trauma with associated peripheral nerve injury that causes profound cellular stress by depriving neurons of contact with their normal target derived tissues which provide neurotrophic support (13). Both of these situations trigger a variety of responses to primarily ensure neuronal survival. In the current study, we found changes after tail amputation in the expression of several DRG genes associated with a neuronal survival promoting role; these include arginase 1 (*ARG1*), neuritin 1 (*NRN1*), neuronal cell adhesion molecule (*NRCAM*), netrin G2 (*NTNG2*), growth differentiation factor 3 (*GDF3*), somatostatin (*SST*), neuropeptide Y (*NPY*) (116). Following tail amputation, transcription factors *ATF3* and *JUN* were significantly up-regulated in caudal DRG neurons. This finding is consistent with previous reports in rat DRG neurons after sciatic nerve transection (13, 117). It had been suggested that these transcription factors (along with *SMAD1* and *STAT4*) function as neuronal regeneration enhancing factors (117). Probe-sets for *SMAD1* and *STAT4* were present on the porcine array, *SMAD1* exhibited significantly increased expression in response to tail amputation, but this was not seen for *STAT4* (neither data shown). Microarray analysis of lumbar DRG neurons after spinal nerve ligation, another commonly used neuropathic pain model, also reported a significant increase in *ATF3* transcripts (22). It has been suggested that transcription factors such as *ATF3, JUN* and *SOX11* may be important in the regulation changes in ion channel function and their accessory subunits after peripheral nerve injury (118).

In the present study, we saw a marked effect of tail amputation on DRG neuronal transcript expression of multiple types of active and passive ion channels and transporters, in particular voltage-dependent and two-pore domain leak potassium (K^+^) channels. This observation has been widely reported in many experimental pain studies in rodents investigating the effect of peripheral nerve injury on the cellular and molecular mechanisms of neuropathic pain (119–121). In DRG neurons, the membrane potential is largely determined by potassium channels and it has been shown that a reduction in, or loss of, function of K^+^ channels can account for changes in neuronal excitability associated with neuropathic pain states (122). Ectopic impulse generation is a key feature of neuropathic pain and is common after peripheral nerve damage. This spontaneous activity can be generated at the site of nerve injury within developing traumatic neuromas, the cell bodies (soma) of injured DRG neurons, as well is in the intact neighboring sensory neurons (123, 124). Significant reductions in DRG neuronal expression of genes coding for multiple voltage-gated K^+^ channel subunits were observed in response to tail amputation in the microarray study (i.e., KCNA4, KCNG4, KCNQ3, KCNH6, KCNV1, KCNT2). The observation of a down-regulation of these voltage-gated channel sub-unit transcripts is consistent with a large number of reports in previous neuropathic pain studies in rodents (22, 23, 119, 120, 125–142). In addition, DRG neuronal transcripts for two K^+^ two-domain pore (K_2_P) leak channels (KCNK1 and KCNK10) were also shown to be consistently down regulated in response to peripheral nerve resection after tail amputation. The importance of these type of K^+^ channels in mediating neuronal excitability and neuropathic pain is widely recognized (32, 143–145).

It has long been recognized that peripheral nerve injury can bring about rapid and sustained changes in glutamate receptor expression at various levels in sensory pathways (146–150). In the current study, two genes *GRM4* and *GRM8* which encode for metabotropic glutamate group III receptors mGluR4 and mGluR8, respectively, were persistently down-regulated in DRG neurons after tail amputation compared to shams. This observation is consistent with reports of down-regulation in mGluR expression in lumbar DRG neurons in rat peripheral axotomy model of neuropathic pain (23).

Peripheral nerve injury-induced loss of inhibition in the spinal cord has been suggested as a mechanism that contributes to the development of neuropathic pain. Inhibitory GABAergic dorsal horn neurons synapse with the central terminals of primary sensory neurons to pre-synaptically modulate primary sensory afferent input to the CNS. The removal of GABAergic control can lead to increase in heightened pain sensitivity (e.g., an exaggerated response to painful stimuli and/or response to innocuous tactile or thermal stimuli) due to disinhibition (151), and this has been demonstrated experimentally in a number of rodent models of neuropathic pain following peripheral nerve injury by complete nerve transection, chronic constriction and part nerve ligation (142–159). In the current microarray study, tail amputation produced a marked and sustained reduction in the transcript expression of two ionotropic (*GABRB2, GABRB3*) and two metabotropic (*GABBR1, GABBR2*) GABA receptor subunits. It is clear in the present study that tail amputation appears to not only produce changes in gene expression that can directly affect neuronal excitability, but also affect spinal inhibitory pathways designed to diminish peripheral sensitivity and chronic pain.

Peripheral nerve injury involving the transection of DRG neurons typically results in an increase in the up-regulation of gene and protein expression of several endogenous pain modulating neuropeptides including proenkephalin, galanin, somatostatin, and neuropeptide Y (160–162). Probe-set intensities for the genes that encode for these neuropeptides (*PENK, GAL, SST*, and *NPY*, respectively) were all consistently increased over a sustained period of time (ca. 4 months) in porcine caudal DRG neurons in response to tail amputation.

Interestingly, although proenkephalin neuropeptide transcripts were increased in response to peripheral nerve injury following tail amputation, *OPRM1* gene expression which encodes for the mu opioid receptor (163) was down-regulated. In the current study, tail amputation produced a sustained reduction in the *OPRM1* transcript expression. This observation is consistent with number of previous studies on rodents using a peripheral nerve transection model of neuropathic pain which reported down-regulation in receptor mRNA and protein expression (164) and increased tactile allodynia and mechanical hyperalgesia in *OPRM1* down-regulated animals compared to controls in DRG and spinal cord neurons (60, 165–167). These previous studies have all demonstrated that significant nerve injury caused by nerve transection can lead to sustained effects on the endogenous opioid system which negate anti-nociceptive/analgesic effects and promote mechanical allodynia and hyperalgesia. We have also previously demonstrated, as part of the same study reported herein, that tail amputation in neonatal and juvenile pigs produces a long-term mechanical hyperalgesia (7).

A limitation of this study is that only mRNA levels were assessed and, although transcriptional changes involving up- and down regulation in gene expression typically (but not always) result in concomitant increases or reductions in the encoded protein, the magnitude of the effect can vary considerably (168). Affymetrix microarrays, as used here, are on the whole very specific and sensitive platforms and have been shown to compare very favorably to RNA-seq. However, it should be recognized that in the absence of RT-qPCR validation it is not possible to validate the true treatment effect size (i.e., true magnitude of the gene response) and in the absence of cellular analyses know whether the observed changes represent an increase in transcription of the gene or an increase in the number of cell which express it.

The data presented in this study describes the global response to tail injury in DRG neurons rather than focusing upon a specific gene. Future work on this data set will examine selected genes in isolated DRG neuron *in vitro* studies in which gene expression and its encoded protein expression will be quantified. It should also be recognized that, in the absence of appropriate pharmacological or gene knockout studies, it is not possible to confirm any causal effect on post-amputation pain in any of the genes identified in this microarray study, even though sustained mechanical hyperalgesia in the tail stump was observed as part of the same study (7). It should also be recognized that the analysis approaches reported in this manuscript lacks mechanistic validity. By presenting those differentially expressed genes that are congruent over time and common to both treatment age groups, it is not possible to report on any temporal differences in gene expression within or between treatment age groups. An analysis of the differences in the differentially expressed genes between the treatment age groups and across time will be conducted and reported in a future, allied manuscript.

In addition, it is not possible to discriminate in which DRG cell populations the effect of the peripheral nerve injury is taking place (e.g., neurons, satellite glial cells, immune cells), although GO enrichment analysis for cellular component indicated that the majority of the gene expression was neuron-derived from features such as neuron cell body, neuron projections (neurites), axons, dendrites, and ion channel complexes and synapses. It is also acknowledged that it was not possible to differentiate differences between responses in injured and non-injured caudal neurons. It is likely that some non-injured neurons close to the site of injury and repair have altered gene expression as a consequence of their proximity to the inflammatory milieu; however for the purpose of investigating the net effects of tail amputation on potential long-term pain this does not present a problem. Similarly, it is also not possible to discern the effect of peripheral nerve injury on different DRG somatosensory neuron subpopulations (e.g., nociceptive and proprioceptive afferents), although we have previously reported preliminary data on pig tail caudal nerve composition and shown that the caudal nerve trunks contain a high proportion of nociceptive C and A-fibers whose function was characterized *in vitro* by axonal excitability studies (169).

## Conclusions

Tail amputation in neonatal and juvenile pigs causes significant changes in DRG gene expression (both up and down) when compared with sham-treated intact controls and these changes are sustained in both age groups up to 16 weeks after tail amputation injury. Network co-expression analysis revealed two highly correlated, interrelated DRG gene expression profiles in response to tail amputation associated with neurogenesis/neuronal function (Cluster A) and wound healing (Cluster B). Gene set enrichment analysis based on the GO terms of inflammatory and neuropathic pain identified a high proportion of genes involved in inflammatory and neuropathic pain signaling pathways in both expression clusters. Key functional families of ion channels and receptors were significantly down-regulated in Cluster A, in particular voltage-gated potassium channels and GABA receptors which are linked to increased neuronal excitability. Up-regulated functional gene families in Cluster B were mostly linked to inflammation, macrophage activity, neurohormone, and opioid peptide activity. Tail amputation causes acute and sustained changes in caudal DRG cells involving key mediators of inflammatory and neuropathic pain which have potential implications for the experience of persistent tail stump pain.

## Ethics Statement

This study was carried out in accordance with the recommendations of the NC3Rs ARRIVE guidelines on reporting research using animals. All animal procedures were carried out under license (PPL 70/7919) granted under the Animal (Scientific Procedures) Act and approved by the Animal Welfare Ethical Review Boards of Newcastle and Scotland's Rural College.

## Author Contributions

DS, SE, and PD designed the experiment. DS and PD performed the experiment. JC extracted tissue RNA for microarray analysis which was carried out by AD at Edinburgh Genomics. DS, AD, and PD analyzed the .CEL file and normalized data. DS, MB, AN, and TF conducted the gene network analysis and GO functional enrichment analysis. DS wrote the manuscript. All authors contributed to the editing of the manuscript and reviewed the final manuscript.

### Conflict of Interest Statement

The authors declare that the research was conducted in the absence of any commercial or financial relationships that could be construed as a potential conflict of interest.

## Footnotes

^1^*Genome build Sscrofa9 (susScr2)*.

## Supplementary Material

Supplementary Figure 1 **(Top)** Gene correlation network of DRG transcriptomics data. Co-expression graph (*r* ≥ 0.75) with 16 largest clusters containing >50 transcripts highlighted (C1–C16). **(Below)** Histograms of mean expression level of all genes in cluster plotted for each sample. Functional enrichment of gene lists for each cluster was analyzed using ToppFun (https://toppgene.cchmc.org/). For each cluster the three most statistically significant gene ontologies (Biological Process) are shown. The first two clusters are those described in detail in the paper; Cluster 1 (A), green nodes, contains 759 transcripts whose expression was down-regulated following tail docking and comprises many genes associated with neurogenesis. Cluster 2 (B), purple nodes, contains 273 transcripts upregulated following tail docking and comprising many genes associated with response to wound healing. The functional enrichment where available is provided for other clusters. The Clusters 6 and 10 contained mainly unannotated genes for which no GO enrichment were obtained.

Supplementary Data File 1 Log_2_ fold change gene expression values (tail amputation vs. sham controls) in both treatment age groups and at each time point after, and 3-way ANOVA *p* values for all differentially expressed DRG transcripts identified in Clusters A and B and associated with inflammatory and neuropathic pain.

Supplementary Data File 2 GeneCards inflammatory pain genes list.

Supplementary Data File 3 GeneCards neuropathic pain genes list.

Supplementary Data File 4 Cluster A pain genes_3-way ANOVA table.

Supplementary Data File 5 Cluster B pain genes_3-way ANOVA table.
