# Supplementary material for: Transcriptomics Analysis of Porcine Caudal Dorsal Root Ganglia in Tail Amputated Pigs Shows Long-Term Effects on Many Pain-Associated Genes
Source: Front Vet Sci. 2019 Sep 18;6:314. doi: 10.3389/fvets.2019.00314 (PMC6760028; doi:10.3389/fvets.2019.00314)
Supplement: Supplementary Figure 1 — (Top) Gene correlation network of DRG transcriptomics data. Co-expression graph (r ≥ 0.75) with 16 largest clusters containing >50 transcripts highlighted (C1–C16). (Below) Histograms of mean expression level of all genes in cluster plotted for each sample. Functional enrichment of gene lists for each cluster was analyzed using ToppFun (https://toppgene.cchmc.org/). For each cluster the three most statistically significant gene ontologies (Biological Process) are shown. The first two clusters are those described in detail in the paper; Cluster 1 (A), green nodes, contains 759 transcripts whose expression was down-regulated following tail docking and comprises many genes associated with neurogenesis. Cluster 2 (B), purple nodes, contains 273 transcripts upregulated following tail docking and comprising many genes associated with response to wound healing. The functional enrichment where available is provided for other clusters. The Clusters 6 and 10 contained mainly unannotated genes for which no GO enrichment were obtained. [file Data_Sheet_1.PDF]

Supplementary Figure 1

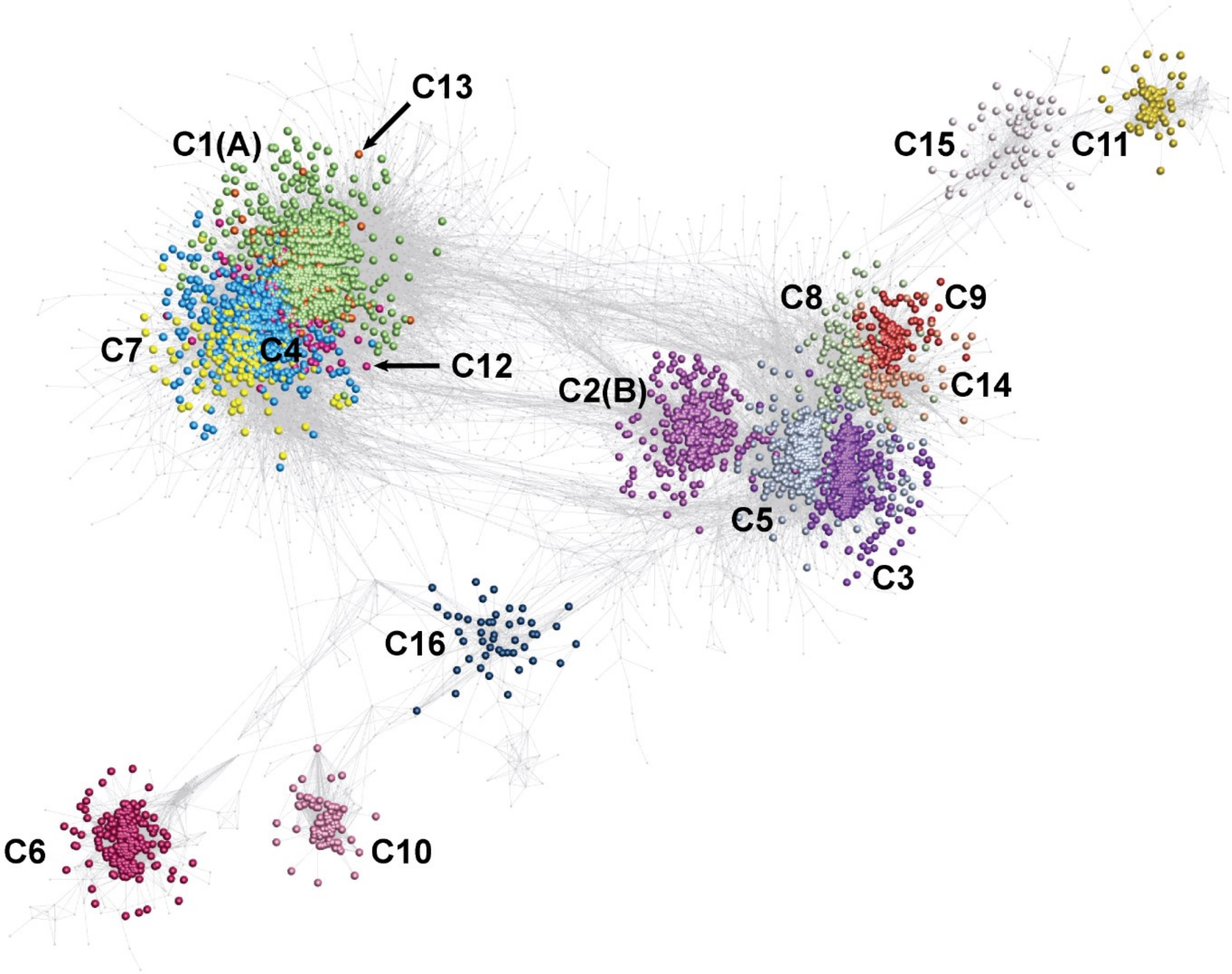

For gene cluster expression profiles see below

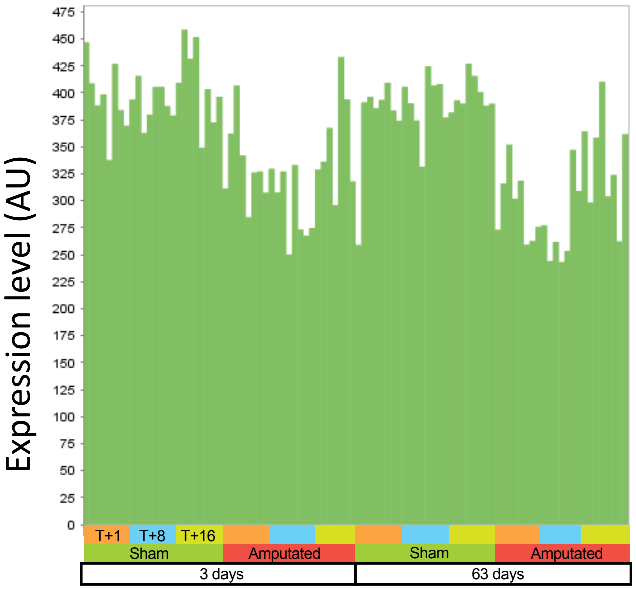

Cluster 1 (A)
Genes down regulated by treatment
759 genes

Biological Process	pValue
Generation of neurons	1.708E-17
Neurogenesis	1.999E-17
Neuron projection development	4.309E-17

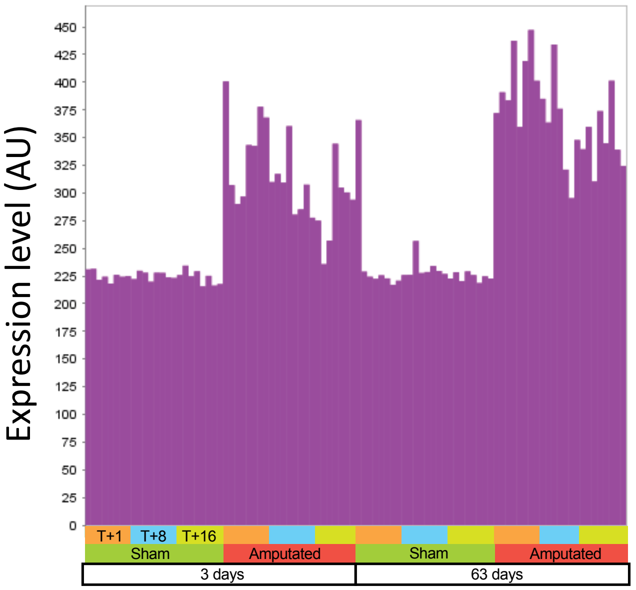

Cluster 2 (B)
Genes up regulated by treatment
273 genes

Biological Process	pValue
Positive regulation of response to wounding	2.973-7
Response to endogenous stimulus	5.224E-7
Response to wounding	5.660E-7

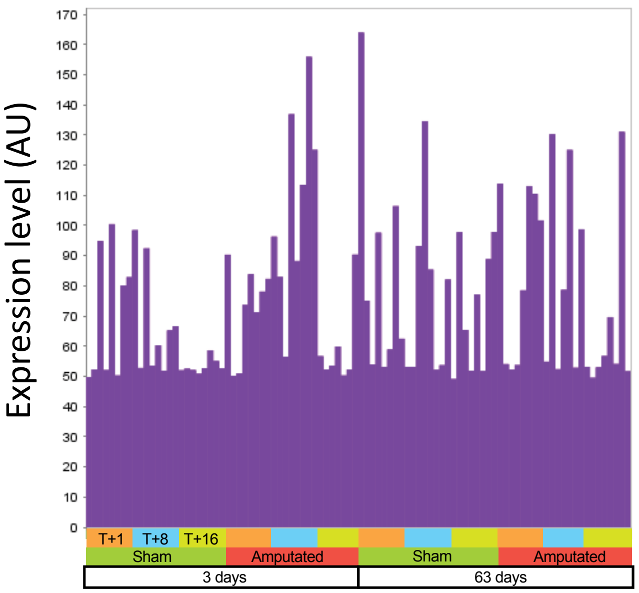

Cluster 3
Cilia Organization (Ependymal cells)
531 genes

Cellular Component	pValue
Cilium	8.221E-37
Ciliary part	5.44E-23
Axoneme	5.635E-16

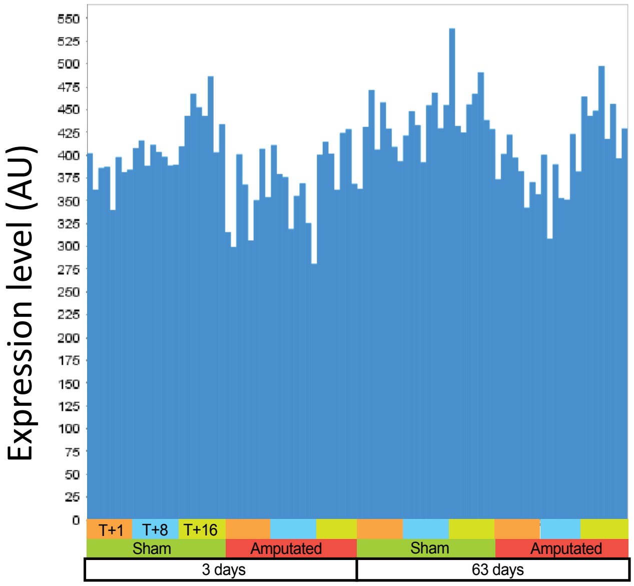

Cluster 4
Oxidative-reductive process (Mitochondria)
416 genes

Cellular Component	pValue
Mitochondrion	7.816E-9
Mitochondrial envelope	1.384E-7
Mitochondrial membrane	1.511E-7

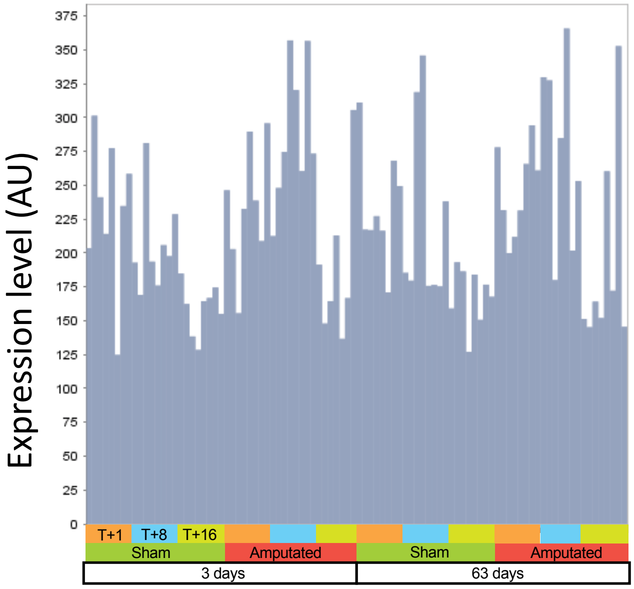

Cluster 5
Extracellular matrix
334 genes

Cellular Component	pValue
Extracellular matrix	1.880E-16
Extracellular space	6.756E-15
Intrinsic component of plasma membrane	3.854E-6

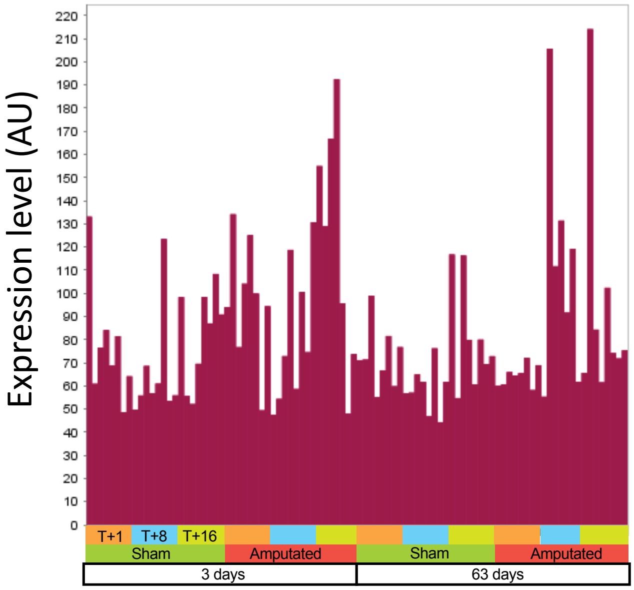

Cluster 6
Olfactory receptors
295 genes

Cellular Component	pValue
No result	N/A
No result	N/A
No result	N/A

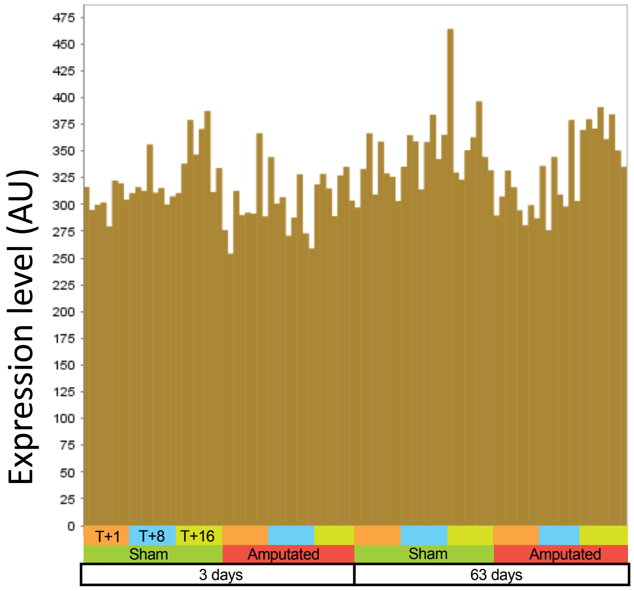

Cluster 7
Cellular Protein Localization
150 genes

Biological Process	pValue
Cellular Protein Localization	1.977E-6
Protein transport	2.016E-6
Cellular macromolecule localization	2.183E-6

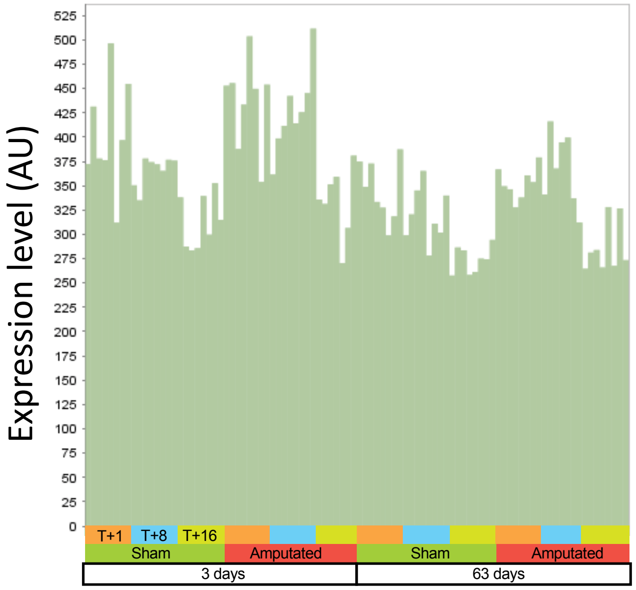

Cluster 8
Extracellular matrix organization
119 genes

Cellular Component	pValue
Extracellular matrix	2.761E-15
Endoplasmic reticulum lumen	4.492E-13
Collagen trimer	4.886E-9

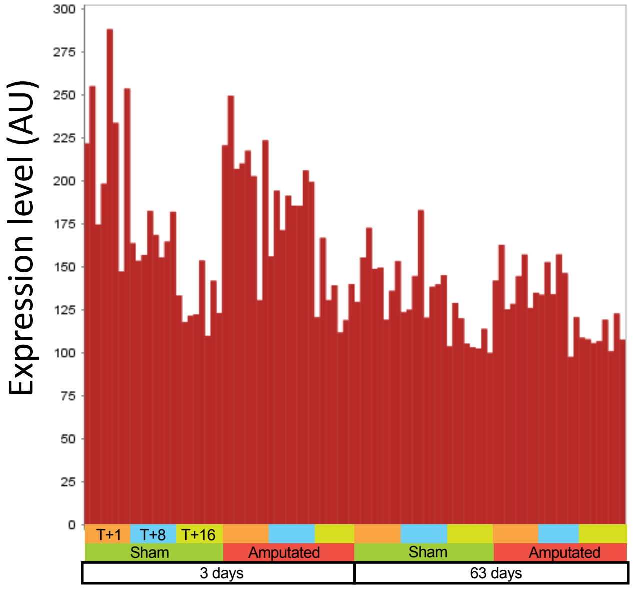

Cluster 9
Cell Cycle
109 genes

Biological Process	pValue
Mitotic cell cycle process	9.956E-19
Cell division	2.276E-18
Mitotic Cell Cycle	5.504E-18

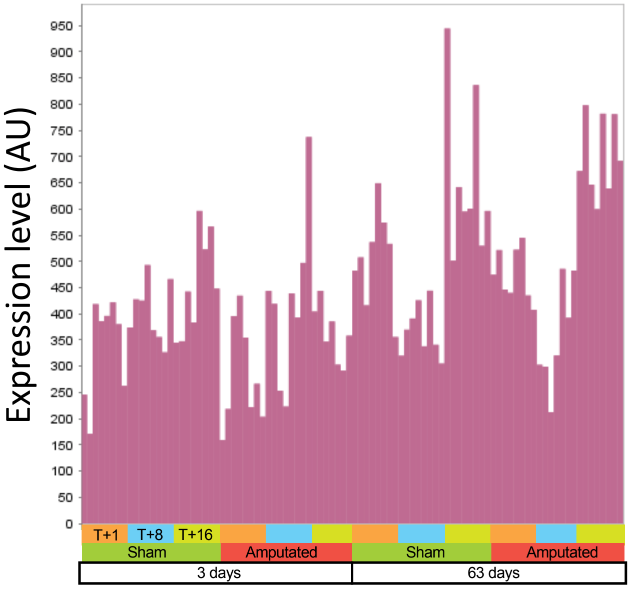

Cluster 10
Unknown
97 genes

Biological Process	pValue
No result	N/A
No result	N/A
No result	N/A

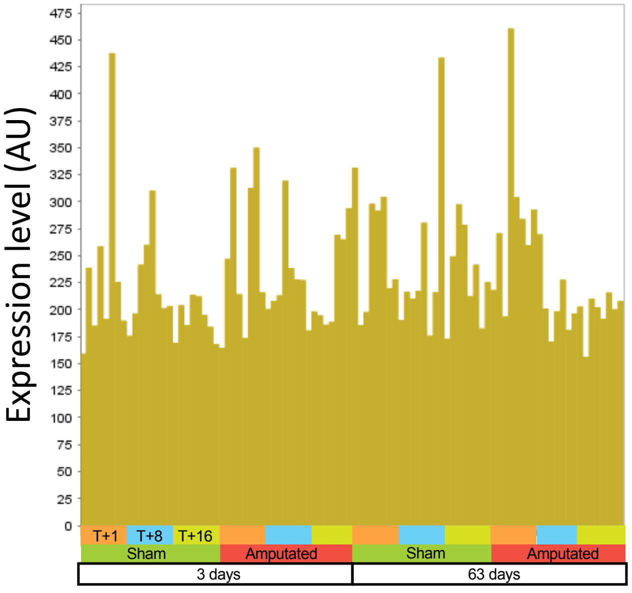

Cluster 11
Type I Interferon signalling
64 genes

Biological Process	pValue
Type I interferon signalling pathway	4.655E-24
Response to virus	1.195E-17
Innate immune response	1.229E-17

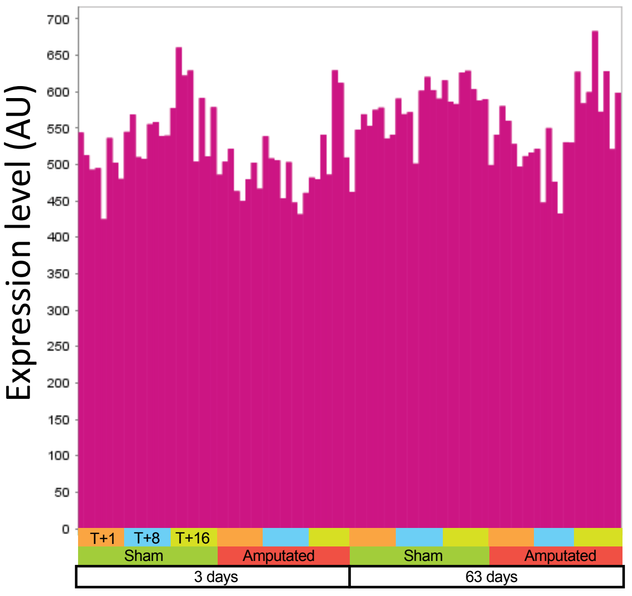

Cluster 12
Vesicle uncoating
58 genes

Biological Process	pValue
Vesicle uncoating	3.692E-2
No result	N/A
No result	N/A

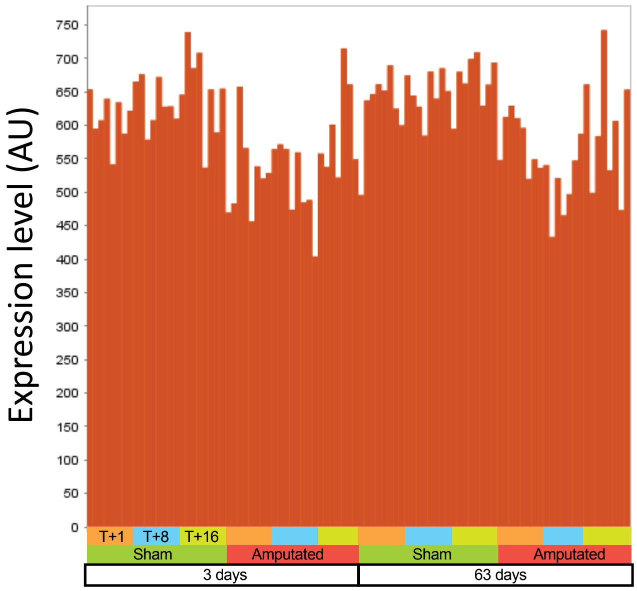

Cluster 13
Mitochondrial proton transporting ATP synthase complex
57 genes

Cellular component	pValue
Proton-transport ATP synthase complex, catalytic core F(1)	3.578E-5
Mitochondrial nucleoid	5.102E-5
Proton-transporting two-sector ATPase complex	5.801E-5

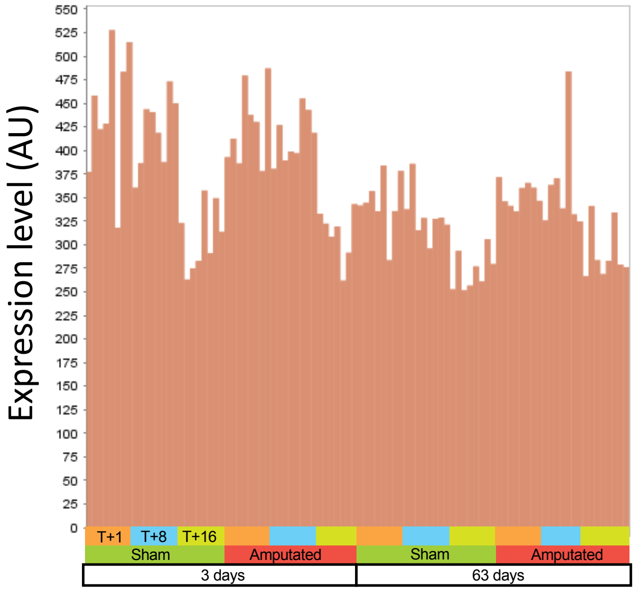

Cluster 14
Down regulated in development
55 genes

Biological Process	pValue
Sphingolipid biosynthetic process	1.654E-5
Membrane lipid biosynthetic Process	6.163E-5
Sphingolipid metabolic Process	1.131E-4

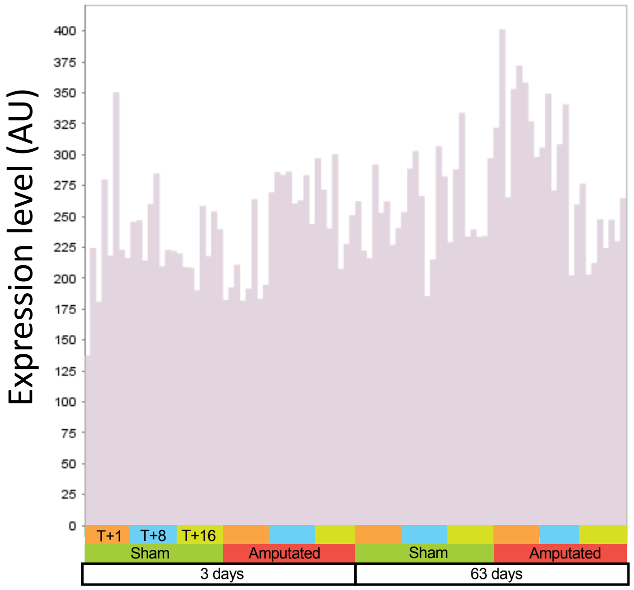

Cluster 15
Macrophages
55 genes

Biological Process	pValue
Cell activation	4.472E-9
Myeloid leukocyte activation	5.932E-9
Immune response	6.173E-8

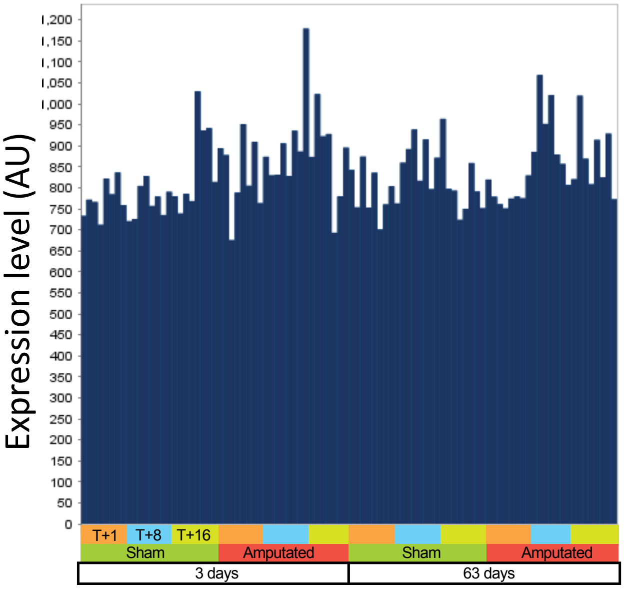

Cluster 16
Protein Translation
46 genes

Biological Process	pValue
SRP-dependent cotranslational protin targeting to membrane	2.362E-25
Cotranslational protein targeting to membrane	5.888E-25
Protein targeting to ER	6.672E-25
