# Supplementary material for: Transcriptomics Analysis of Porcine Caudal Dorsal Root Ganglia in Tail Amputated Pigs Shows Long-Term Effects on Many Pain-Associated Genes
Source: Front Vet Sci. 2019 Sep 18;6:314. doi: 10.3389/fvets.2019.00314 (PMC6760028; doi:10.3389/fvets.2019.00314)
Supplement: Supplementary Data File 2 — GeneCards inflammatory pain genes list. [file Data_Sheet_2.PDF]

Porcine DRG pain gene analysis
Supplementary Data File 2
GeneCards inflammatory pain genes list

Gene Symbol	Description	Gifts	Relevance score
A2M	Alpha-2-Macroglobulin	64	8.15
A4GALT	Alpha 1,4-Galactosyltransferase (P Blood Group)	60	6.76
AARS	Alanyl-TRNA Synthetase	62	12.03
ABCA1	ATP Binding Cassette Subfamily A Member 1	69	5.79
ABCA12	ATP Binding Cassette Subfamily A Member 12	59	4.08
ABCB1	ATP Binding Cassette Subfamily B Member 1	74	31.01
ABCB11	ATP Binding Cassette Subfamily B Member 11	65	2.67
ABCB4	ATP Binding Cassette Subfamily B Member 4	63	8.95
ABCB7	ATP Binding Cassette Subfamily B Member 7	59	0.93
ABCB8	ATP Binding Cassette Subfamily B Member 8	60	0.73
ABCC1	ATP Binding Cassette Subfamily C Member 1	69	3.12
ABCC2	ATP Binding Cassette Subfamily C Member 2	70	4.19
ABCC3	ATP Binding Cassette Subfamily C Member 3	66	2.01
ABCC4	ATP Binding Cassette Subfamily C Member 4	67	1.51
ABCC6	ATP Binding Cassette Subfamily C Member 6	62	4.1
ABCC8	ATP Binding Cassette Subfamily C Member 8	66	1.28
ABCD1	ATP Binding Cassette Subfamily D Member 1	64	0.95
ABCG1	ATP Binding Cassette Subfamily G Member 1	61	1.05
ABCG2	ATP Binding Cassette Subfamily G Member 2 (Junior Blood Group)	70	5.03
ABHD12	Abhydrolase Domain Containing 12	55	4.37
ABL1	ABL Proto-Oncogene 1, Non-Receptor Tyrosine Kinase	74	2.67
ACADM	Acyl-CoA Dehydrogenase, C-4 To C-12 Straight Chain	62	3.78
ACAN	Aggrecan	63	14.99
ACE	Angiotensin I Converting Enzyme	70	23.5
ACE2	Angiotensin I Converting Enzyme 2	69	1.22
ACKR1	Atypical Chemokine Receptor 1 (Duffy Blood Group)	50	4.67
ACO1	Aconitase 1	64	2.32
ACP1	Acid Phosphatase 1, Soluble	63	2.15
ACP5	Acid Phosphatase 5, Tartrate Resistant	66	11.02
ACPP	Acid Phosphatase, Prostate	66	2.91
ACR	Acrosin	53	1.01
ACSL4	Acyl-CoA Synthetase Long-Chain Family Member 4	58	1.01
ACSS2	Acyl-CoA Synthetase Short-Chain Family Member 2	60	0.57
ACT	Actin-Like Protein (ACT) Gene	15	1.88
ACTA1	Actin, Alpha 1, Skeletal Muscle	65	4.11
ACTA2	Actin, Alpha 2, Smooth Muscle, Aorta	67	9.03
ACTC1	Actin, Alpha, Cardiac Muscle 1	60	16.65
ACTG2	Actin, Gamma 2, Smooth Muscle, Enteric	65	3.05
ACTR2	ARP2 Actin Related Protein 2 Homolog	62	2.05
ACTR3	ARP3 Actin Related Protein 3 Homolog	60	2.26
ACVR1	Activin A Receptor Type 1	72	0.93
ACVRL1	Activin A Receptor Like Type 1	70	15.74
ADA	Adenosine Deaminase	71	12.5
ADA2	Adenosine Deaminase 2	43	18.25
ADAM10	ADAM Metallopeptidase Domain 10	73	3.09
ADAM12	ADAM Metallopeptidase Domain 12	66	1
ADAM17	ADAM Metallopeptidase Domain 17	71	21.1
ADAM19	ADAM Metallopeptidase Domain 19	60	1.46
ADAM33	ADAM Metallopeptidase Domain 33	56	2.9
ADAM9	ADAM Metallopeptidase Domain 9	68	0.92
ADAMTS1	ADAM Metallopeptidase With Thrombospondin Type 1 Motif 1	62	5.14
ADAMTS13	ADAM Metallopeptidase With Thrombospondin Type 1 Motif 13	62	9.26
ADAMTS4	ADAM Metallopeptidase With Thrombospondin Type 1 Motif 4	62	5.43
ADAMTS5	ADAM Metallopeptidase With Thrombospondin Type 1 Motif 5	62	3.34
ADAMTSL1	ADAMTS Like 1	55	2.05
ADAR	Adenosine Deaminase, RNA Specific	61	5.18
ADCY1	Adenylate Cyclase 1	66	2.4
ADCY10	Adenylate Cyclase 10, Soluble	60	2.92

ADCY2	Adenylate Cyclase 2	62	2.12
ADCY3	Adenylate Cyclase 3	62	2.32
ADCY4	Adenylate Cyclase 4	58	2.12
ADCY5	Adenylate Cyclase 5	68	2.38
ADCY6	Adenylate Cyclase 6	63	2.12
ADCY7	Adenylate Cyclase 7	63	2.32
ADCY8	Adenylate Cyclase 8	63	2.12
ADCY9	Adenylate Cyclase 9	62	2.49
ADCYAP1	Adenylate Cyclase Activating Polypeptide 1	58	3.35
ADGRE2	Adhesion G Protein-Coupled Receptor E2	50	1.77
ADH1B	Alcohol Dehydrogenase 1B (Class I), Beta Polypeptide	61	1.25
ADH1C	Alcohol Dehydrogenase 1C (Class I), Gamma Polypeptide	58	2.17
ADIPOQ	Adiponectin, C1Q And Collagen Domain Containing	67	6.37
ADIPOR1	Adiponectin Receptor 1	66	0.66
ADIPOR2	Adiponectin Receptor 2	61	0.72
ADK	Adenosine Kinase	71	2.83
ADM	Adrenomedullin	61	8.95
ADORA1	Adenosine A1 Receptor	68	2.96
ADORA2A	Adenosine A2a Receptor	70	5.76
ADPRH	ADP-Ribosylarginine Hydrolase	53	0.79
ADRA2B	Adrenoceptor Alpha 2B	64	1.86
ADRB1	Adrenoceptor Beta 1	70	3.43
ADRB2	Adrenoceptor Beta 2	73	19.01
ADRB3	Adrenoceptor Beta 3	67	0.73
ADSL	Adenylosuccinate Lyase	63	3.4
AFF4	AF4/FMR2 Family Member 4	57	1.47
AFP	Alpha Fetoprotein	62	9.42
AGER	Advanced Glycosylation End-Product Specific Receptor	63	4.95
AGGF1	Angiogenic Factor With G-Patch And FHA Domains 1	56	1.25
AGPAT2	1-Acylglycerol-3-Phosphate O-Acyltransferase 2	62	3.87
AGR2	Anterior Gradient 2, Protein Disulphide Isomerase Family Member	59	1.1
AGT	Angiotensinogen	70	9.88
AGTR1	Angiotensin II Receptor Type 1	71	12.63
AGTR2	Angiotensin II Receptor Type 2	65	1.22
AGXT	Alanine-Glyoxylate Aminotransferase	63	3.03
AHCY	Adenosylhomocysteinase	70	1.46
AHR	Aryl Hydrocarbon Receptor	67	4.12
AHSA1	Activator Of HSP90 ATPase Activity 1	56	2.05
AHSG	Alpha 2-HS Glycoprotein	60	1.9
AHSP	Alpha Hemoglobin Stabilizing Protein	48	1.42
AICDA	Activation Induced Cytidine Deaminase	64	1.2
AIF1	Allograft Inflammatory Factor 1	57	12.8
AIFM1	Apoptosis Inducing Factor Mitochondria Associated 1	70	4.76
AIM2	Absent In Melanoma 2	60	1.31
AIMP1	Aminoacyl TRNA Synthetase Complex Interacting Multifunctional Protein 1	58	2.47
AIP	Aryl Hydrocarbon Receptor Interacting Protein	61	11.87
AIRE	Autoimmune Regulator	59	8.55
AK2	Adenylate Kinase 2	66	1.83
AKAP12	A-Kinase Anchoring Protein 12	58	1.12
AKR1B1	Aldo-Keto Reductase Family 1 Member B	67	4.73
AKR1B10	Aldo-Keto Reductase Family 1 Member B10	63	0.73
AKR1C1	Aldo-Keto Reductase Family 1 Member C1	61	1.35
AKR1C2	Aldo-Keto Reductase Family 1 Member C2	62	0.88
AKR1C3	Aldo-Keto Reductase Family 1 Member C3	66	2.02
AKT1	AKT Serine/Threonine Kinase 1	79	17.88
AKT2	AKT Serine/Threonine Kinase 2	75	1.74
AKTIP	AKT Interacting Protein	55	3.51
ALB	Albumin	70	21.74
ALDH1A1	Aldehyde Dehydrogenase 1 Family Member A1	67	0.85

ALDH2	Aldehyde Dehydrogenase 2 Family (Mitochondrial)	69	4.03
ALDH3A2	Aldehyde Dehydrogenase 3 Family Member A2	63	8.72
ALDH7A1	Aldehyde Dehydrogenase 7 Family Member A1	66	3.28
ALK	ALK Receptor Tyrosine Kinase	74	17.01
ALMS1	ALMS1, Centrosome And Basal Body Associated Protein	55	3.26
ALOX12	Arachidonate 12-Lipoxygenase, 12S Type	61	7.14
ALOX12B	Arachidonate 12-Lipoxygenase, 12R Type	61	3.77
ALOX15B	Arachidonate 15-Lipoxygenase, Type B	60	1.16
ALOX5	Arachidonate 5-Lipoxygenase	64	16.24
ALOX5AP	Arachidonate 5-Lipoxygenase Activating Protein	62	9.93
ALOXE3	Arachidonate Lipoxygenase 3	58	3.34
ALPL	Alkaline Phosphatase, Liver/Bone/Kidney	72	10.08
ALPP	Alkaline Phosphatase, Placental	66	6.74
AMACR	Alpha-Methylacyl-CoA Racemase	64	4.38
AMD1	Adenosylmethionine Decarboxylase 1	62	3.67
AMH	Anti-Mullerian Hormone	60	3.13
AMPD1	Adenosine Monophosphate Deaminase 1	62	8.41
ANG	Angiogenin	63	2.84
ANGPT1	Angiopoietin 1	67	5.64
ANGPT2	Angiopoietin 2	66	5.76
ANKH	ANKH Inorganic Pyrophosphate Transport Regulator	54	12.97
ANKRD11	Ankyrin Repeat Domain 11	52	1.93
ANKRD17	Ankyrin Repeat Domain 17	51	0.73
ANKRD20A1	Ankyrin Repeat Domain 20 Family Member A1	38	3.93
ANKRD55	Ankyrin Repeat Domain 55	47	5.18
ANO5	Anoctamin 5	54	4.55
ANPEP	Alanyl Aminopeptidase, Membrane	70	3.15
ANXA1	Annexin A1	70	8.28
ANXA2	Annexin A2	66	1.26
ANXA3	Annexin A3	59	0.97
ANXA5	Annexin A5	67	5.73
AOC1	Amine Oxidase, Copper Containing 1	55	3.91
AOC3	Amine Oxidase, Copper Containing 3	63	1.77
AOX1	Aldehyde Oxidase 1	64	1.01
AP1S3	Adaptor Related Protein Complex 1 Sigma 3 Subunit	54	6.02
AP2S1	Adaptor Related Protein Complex 2 Sigma 1 Subunit	58	2.15
AP3B1	Adaptor Related Protein Complex 3 Beta 1 Subunit	61	4.67
APAF1	Apoptotic Peptidase Activating Factor 1	67	0.85
APBB1	Amyloid Beta Precursor Protein Binding Family B Member 1	62	0.85
APC	APC, WNT Signaling Pathway Regulator	70	12.13
APEH	Acylaminoacyl-Peptide Hydrolase	56	1.03
APEX1	Apurinic/Apyrimidinic Endodeoxyribonuclease 1	64	1.28
APH1A	Aph-1 Homolog A, Gamma-Secretase Subunit	61	3.34
APLN	Apelin	54	1.34
APOA1	Apolipoprotein A1	70	16.56
APOA2	Apolipoprotein A2	63	1.75
APOA5	Apolipoprotein A5	61	1.11
APOB	Apolipoprotein B	65	4.52
APOBEC3G	Apolipoprotein B MRNA Editing Enzyme Catalytic Subunit 3G	58	1.03
APOC2	Apolipoprotein C2	59	4.04
APOC3	Apolipoprotein C3	60	1.39
APOD	Apolipoprotein D	61	0.81
APOE	Apolipoprotein E	71	26.96
APOH	Apolipoprotein H	62	12.16
APP	Amyloid Beta Precursor Protein	74	23.16
APRT	Adenine Phosphoribosyltransferase	65	8.97
AQP1	Aquaporin 1 (Colton Blood Group)	66	6.17
AQP2	Aquaporin 2	66	5.63
AQP3	Aquaporin 3 (Gill Blood Group)	65	0.85

AQP4	Aquaporin 4	64	8.05
AR	Androgen Receptor	76	4.48
AREG	Amphiregulin	64	7.23
ARG1	Arginase 1	70	1.05
ARHGEF10	Rho Guanine Nucleotide Exchange Factor 10	57	4.72
ARMS2	Age-Related Maculopathy Susceptibility 2	45	2.8
ARNT	Aryl Hydrocarbon Receptor Nuclear Translocator	63	0.57
ARRB2	Arrestin Beta 2	64	1.48
ARSA	Arylsulfatase A	65	9.46
ARVCF	Armadillo Repeat Gene Deleted In Velocardiofacial Syndrome	52	4.79
ASIC1	Acid Sensing Ion Channel Subunit 1	61	3.69
ASIC2	Acid Sensing Ion Channel Subunit 2	55	1.48
ASIC3	Acid Sensing Ion Channel Subunit 3	58	6.68
ASIC4	Acid Sensing Ion Channel Subunit Family Member 4	56	1.05
ATF1	Activating Transcription Factor 1	67	1.84
ATF3	Activating Transcription Factor 3	65	2.69
ATF4	Activating Transcription Factor 4	67	1.16
ATF6	Activating Transcription Factor 6	66	0.99
ATG16L1	Autophagy Related 16 Like 1	62	20.25
ATL3	Atlastin GTPase 3	53	4.16
ATM	ATM Serine/Threonine Kinase	77	6.97
ATN1	Atrophin 1	60	1.26
ATOX1	Antioxidant 1 Copper Chaperone	57	1.03
ATP12A	ATPase H+/K+ Transporting Non-Gastric Alpha2 Subunit	59	5.83
ATP1A2	ATPase Na+/K+ Transporting Subunit Alpha 2	66	9.9
ATP2A2	ATPase Sarcoplasmic/Endoplasmic Reticulum Ca2+ Transporting 2	70	2.64
ATP4A	ATPase H+/K+ Transporting Alpha Subunit	59	9.38
ATP6V0A2	ATPase H+ Transporting V0 Subunit A2	59	2.26
ATP7A	ATPase Copper Transporting Alpha	64	4.88
ATP7B	ATPase Copper Transporting Beta	66	9.21
ATP8B1	ATPase Phospholipid Transporting 8B1	57	3.24
ATR	ATR Serine/Threonine Kinase	72	1.03
ATXN3	Ataxin 3	62	4.75
AVP	Arginine Vasopressin	65	10.52
AVPR2	Arginine Vasopressin Receptor 2	67	3.67
AXIN1	Axin 1	63	3.26
AXL	AXL Receptor Tyrosine Kinase	71	1.56
AZU1	Azurocidin 1	56	4.4
B2M	Beta-2-Microglobulin	71	14.38
B3GAT1	Beta-1,3-Glucuronyltransferase 1	61	1.88
BACE1	Beta-Secretase 1	66	5.87
BACE1-AS	BACE1 Antisense RNA	13	4.42
BACE2	Beta-Site APP-Cleaving Enzyme 2	61	5.03
BACH2	BTB Domain And CNC Homolog 2	54	0.81
BAD	BCL2 Associated Agonist Of Cell Death	67	0.95
BAG1	BCL2 Associated Athanogene 1	62	0.73
BAK1	BCL2 Antagonist/Killer 1	64	1.24
BANK1	B-Cell Scaffold Protein With Ankyrin Repeats 1	52	4.57
BAP1	BRCA1 Associated Protein 1	62	3
BAX	BCL2 Associated X, Apoptosis Regulator	67	12.19
BAZ1B	Bromodomain Adjacent To Zinc Finger Domain 1B	57	6.72
BBS2	Bardet-Biedl Syndrome 2	55	1.18
BCAM	Basal Cell Adhesion Molecule (Lutheran Blood Group)	58	0.73
BCHE	Butyrylcholinesterase	69	1.94
BCKDHA	Branched Chain Keto Acid Dehydrogenase E1, Alpha Polypeptide	62	4.43
BCL10	B-Cell CLL/Lymphoma 10	62	8
BCL2	BCL2, Apoptosis Regulator	75	11.97
BCL2A1	BCL2 Related Protein A1	62	2.22
BCL2L1	BCL2 Like 1	70	8.46

BCL3	B-Cell CLL/Lymphoma 3	58	1.18
BCL6	B-Cell CLL/Lymphoma 6	67	9.47
BCL6B	B-Cell CLL/Lymphoma 6B	52	0.81
BCL7A	BCL Tumor Suppressor 7A	51	2.97
BCL7B	BCL Tumor Suppressor 7B	51	3.51
BCOR	BCL6 Corepressor	56	1.74
BCR	BCR, RhoGEF And GTPase Activating Protein	69	3.77
BCS1L	BCS1 Homolog, Ubiquinol-Cytochrome C Reductase Complex Chaperone	59	3.15
BDKRB1	Bradykinin Receptor B1	62	7.58
BDKRB2	Bradykinin Receptor B2	65	3.87
BDNF	Brain Derived Neurotrophic Factor	70	23.7
BECN1	Beclin 1	68	0.85
BGLAP	Bone Gamma-Carboxyglutamate Protein	62	13.43
BID	BH3 Interacting Domain Death Agonist	66	1.44
BIRC2	Baculoviral IAP Repeat Containing 2	66	2.3
BIRC3	Baculoviral IAP Repeat Containing 3	66	9.25
BIRC5	Baculoviral IAP Repeat Containing 5	68	3.88
BLNK	B-Cell Linker	66	4.45
BLVRB	Biliverdin Reductase B	57	1.66
BMI1	BMI1 Proto-Oncogene, Polycomb Ring Finger	63	1.19
BMP2	Bone Morphogenetic Protein 2	70	6.15
BMP4	Bone Morphogenetic Protein 4	70	1.64
BMP6	Bone Morphogenetic Protein 6	63	7.54
BMP7	Bone Morphogenetic Protein 7	66	1.99
BMPR1A	Bone Morphogenetic Protein Receptor Type 1A	71	5.5
BMPR1B	Bone Morphogenetic Protein Receptor Type 1B	69	0.93
BMPR2	Bone Morphogenetic Protein Receptor Type 2	68	7.98
BMX	BMX Non-Receptor Tyrosine Kinase	65	0.85
BPGM	Bisphosphoglycerate Mutase	63	0.65
BPI	Bactericidal/Permeability-Increasing Protein	60	8.2
BPIFA1	BPI Fold Containing Family A Member 1	52	1.91
BRAF	B-Raf Proto-Oncogene, Serine/Threonine Kinase	78	14.75
BRCA1	BRCA1, DNA Repair Associated	70	8.4
BRCA2	BRCA2, DNA Repair Associated	69	4.43
BRD4	Bromodomain Containing 4	62	7.61
BSCL2	BSCL2, Seipin Lipid Droplet Biogenesis Associated	58	3.84
BSG	Basigin (Ok Blood Group)	63	3.73
BST2	Bone Marrow Stromal Cell Antigen 2	55	3.74
BTD	Biotinidase	62	6.05
BTK	Bruton Tyrosine Kinase	77	3.98
BTNL2	Butyrophilin Like 2	52	17.03
BTRC	Beta-Transducin Repeat Containing E3 Ubiquitin Protein Ligase	65	0.85
BUD23	BUD23, RRNA Methyltransferase And Ribosome Maturation Factor	39	1.02
C1QA	Complement C1q A Chain	66	4.7
C1QB	Complement C1q B Chain	62	4.7
C1QC	Complement C1q C Chain	62	5.14
C1R	Complement C1r	63	8.65
C1S	Complement C1s	65	7.19
C2	Complement C2	64	2.64
C21orf33	Chromosome 21 Open Reading Frame 33	53	1.32
C3	Complement C3	70	8.52
C4A	Complement C4A (Rodgers Blood Group)	57	22.73
C4B	Complement C4B (Chido Blood Group)	59	7.24
C5	Complement C5	66	3.28
C5AR1	Complement C5a Receptor 1	63	2.37
C5orf30	Chromosome 5 Open Reading Frame 30	47	4.22
CA2	Carbonic Anhydrase 2	72	4.04
CA8	Carbonic Anhydrase 8	64	3.23
CA9	Carbonic Anhydrase 9	67	1.74

CABIN1	Calcineurin Binding Protein 1	59	4.57
CACNA1B	Calcium Voltage-Gated Channel Subunit Alpha1 B	70	7.79
CACNA1C	Calcium Voltage-Gated Channel Subunit Alpha1 C	67	1.58
CACNA1H	Calcium Voltage-Gated Channel Subunit Alpha1 H	68	2.41
CALB2	Calbindin 2	58	5.38
CALCA	Calcitonin Related Polypeptide Alpha	66	16.77
CALCB	Calcitonin Related Polypeptide Beta	57	1.14
CALCRL	Calcitonin Receptor Like Receptor	62	2.72
CALD1	Caldesmon 1	62	2.6
CALM1	Calmodulin 1	66	1.18
CALM2	Calmodulin 2	63	1.18
CALM3	Calmodulin 3	63	1.38
CALML5	Calmodulin Like 5	54	0.99
CALR	Calreticulin	72	12.69
CAMK1	Calcium/Calmodulin Dependent Protein Kinase I	62	0.91
CAMK2A	Calcium/Calmodulin Dependent Protein Kinase II Alpha	69	2.4
CAMK2B	Calcium/Calmodulin Dependent Protein Kinase II Beta	69	2.4
CAMK2D	Calcium/Calmodulin Dependent Protein Kinase II Delta	68	2.77
CAMK2G	Calcium/Calmodulin Dependent Protein Kinase II Gamma	69	3.8
CAMP	Cathelicidin Antimicrobial Peptide	59	3.22
CANT1	Calcium Activated Nucleotidase 1	62	0.77
CAPN3	Calpain 3	65	2.62
CARD11	Caspase Recruitment Domain Family Member 11	65	2.13
CARD8	Caspase Recruitment Domain Family Member 8	54	4.54
CARD9	Caspase Recruitment Domain Family Member 9	62	2.43
CARMIL1	Capping Protein Regulator And Myosin 1 Linker 1	37	2.6
CARS	Cysteinyl-TRNA Synthetase	58	10.43
CASP1	Caspase 1	73	12.19
CASP10	Caspase 10	70	8.44
CASP14	Caspase 14	62	0.73
CASP3	Caspase 3	75	9.81
CASP4	Caspase 4	65	2.62
CASP7	Caspase 7	73	4.39
CASP8	Caspase 8	75	15.48
CASP9	Caspase 9	70	10.57
CASR	Calcium Sensing Receptor	73	9.23
CAT	Catalase	70	12.06
CAV1	Caveolin 1	68	9.82
CAV3	Caveolin 3	61	10.88
CBS	Cystathionine-Beta-Synthase	68	4.78
CCBE1	Collagen And Calcium Binding EGF Domains 1	54	2.14
CCDC180	Coiled-Coil Domain Containing 180	42	3.93
CCDC85B	Coiled-Coil Domain Containing 85B	46	0.73
CCK	Cholecystokinin	59	14.07
CCKBR	Cholecystokinin B Receptor	66	2.32
CCL1	C-C Motif Chemokine Ligand 1	60	3.59
CCL11	C-C Motif Chemokine Ligand 11	64	28.08
CCL13	C-C Motif Chemokine Ligand 13	58	2.07
CCL15	C-C Motif Chemokine Ligand 15	50	1.71
CCL17	C-C Motif Chemokine Ligand 17	60	8.55
CCL18	C-C Motif Chemokine Ligand 18	51	6.75
CCL19	C-C Motif Chemokine Ligand 19	59	3.23
CCL2	C-C Motif Chemokine Ligand 2	70	27.2
CCL20	C-C Motif Chemokine Ligand 20	61	9.91
CCL22	C-C Motif Chemokine Ligand 22	58	7.58
CCL24	C-C Motif Chemokine Ligand 24	55	3.98
CCL26	C-C Motif Chemokine Ligand 26	58	5.77
CCL27	C-C Motif Chemokine Ligand 27	54	4.79
CCL3	C-C Motif Chemokine Ligand 3	58	19.8

CCL4	C-C Motif Chemokine Ligand 4	60	8.79
CCL4L1	C-C Motif Chemokine Ligand 4 Like 1	41	2.15
CCL5	C-C Motif Chemokine Ligand 5	63	19.36
CCND1	Cyclin D1	73	22.7
CCND2	Cyclin D2	71	4.28
CCND3	Cyclin D3	68	1.85
CCNE1	Cyclin E1	68	3.56
CCR1	C-C Motif Chemokine Receptor 1	64	21.33
CCR2	C-C Motif Chemokine Receptor 2	65	6.31
CCR3	C-C Motif Chemokine Receptor 3	66	6.92
CCR4	C-C Motif Chemokine Receptor 4	64	4.31
CCR5	C-C Motif Chemokine Receptor 5 (Gene/Pseudogene)	70	21.02
CCR6	C-C Motif Chemokine Receptor 6	63	12.01
CCR7	C-C Motif Chemokine Receptor 7	66	1.84
CCR9	C-C Motif Chemokine Receptor 9	62	3.68
CCS	Copper Chaperone For Superoxide Dismutase	58	0.85
CD14	CD14 Molecule	66	6.5
CD160	CD160 Molecule	56	2.2
CD163	CD163 Molecule	60	5.91
CD177	CD177 Molecule	56	4.29
CD19	CD19 Molecule	70	9.37
CD1A	CD1a Molecule	58	0.81
CD2	CD2 Molecule	62	5.07
CD200	CD200 Molecule	57	2.88
CD200R1	CD200 Receptor 1	55	2.8
CD207	CD207 Molecule	57	0.72
CD209	CD209 Molecule	63	12.96
CD226	CD226 Molecule	60	3.97
CD244	CD244 Molecule	59	8.72
CD247	CD247 Molecule	66	7.34
CD27	CD27 Molecule	66	9.5
CD274	CD274 Molecule	66	1.51
CD276	CD276 Molecule	56	3.48
CD28	CD28 Molecule	66	7.57
CD2AP	CD2 Associated Protein	58	2.6
CD300E	CD300e Molecule	50	2.6
CD302	CD302 Molecule	51	2.36
CD33	CD33 Molecule	64	1.27
CD34	CD34 Molecule	65	8.59
CD36	CD36 Molecule	70	15.92
CD38	CD38 Molecule	66	2.27
CD4	CD4 Molecule	70	12.74
CD40	CD40 Molecule	69	11.12
CD40LG	CD40 Ligand	67	27.78
CD44	CD44 Molecule (Indian Blood Group)	70	8.98
CD5	CD5 Molecule	60	5.36
CD52	CD52 Molecule	53	0.93
CD53	CD53 Molecule	56	0.73
CD55	CD55 Molecule (Cromer Blood Group)	66	3.88
CD58	CD58 Molecule	59	5.93
CD59	CD59 Molecule (CD59 Blood Group)	67	4.19
CD6	CD6 Molecule	59	6.32
CD68	CD68 Molecule	60	7.16
CD69	CD69 Molecule	58	5.01
CD7	CD7 Molecule	58	4.46
CD70	CD70 Molecule	60	2.8
CD72	CD72 Molecule	58	0.81
CD79A	CD79a Molecule	66	22.45
CD79B	CD79b Molecule	66	4.44

CD80	CD80 Molecule	62	7.98
CD81	CD81 Molecule	64	7.99
CD86	CD86 Molecule	63	7.54
CD8A	CD8a Molecule	66	12.63
CD9	CD9 Molecule	66	1.44
CD96	CD96 Molecule	58	0.81
CD99L2	CD99 Molecule Like 2	52	0.95
CDC25C	Cell Division Cycle 25C	70	0.95
CDC42	Cell Division Cycle 42	73	3.33
CDC6	Cell Division Cycle 6	62	0.57
CDC73	Cell Division Cycle 73	61	14.69
CDH1	Cadherin 1	74	26.5
CDH11	Cadherin 11	63	0.73
CDH13	Cadherin 13	60	1.19
CDH3	Cadherin 3	66	3.17
CDH5	Cadherin 5	72	3.79
CDIPT	CDP-Diacylglycerol--Inositol 3-Phosphatidyltransferase	59	2.36
CDK1	Cyclin Dependent Kinase 1	66	1.85
CDK2	Cyclin Dependent Kinase 2	78	2.22
CDK4	Cyclin Dependent Kinase 4	79	9.37
CDK5	Cyclin Dependent Kinase 5	74	2.25
CDK6	Cyclin Dependent Kinase 6	75	3.41
CDKN1A	Cyclin Dependent Kinase Inhibitor 1A	70	10.94
CDKN1B	Cyclin Dependent Kinase Inhibitor 1B	70	10.28
CDKN1C	Cyclin Dependent Kinase Inhibitor 1C	65	2.8
CDKN2A	Cyclin Dependent Kinase Inhibitor 2A	69	17.6
CDKN2B	Cyclin Dependent Kinase Inhibitor 2B	66	9.24
CDKN2B-AS1	CDKN2B Antisense RNA 1	27	4.09
CDKN2C	Cyclin Dependent Kinase Inhibitor 2C	62	6.87
CDKN3	Cyclin Dependent Kinase Inhibitor 3	58	2.94
CDO1	Cysteine Dioxygenase Type 1	59	0.66
CDSN	Corneodesmosin	55	11.87
CDX1	Caudal Type Homeobox 1	54	0.95
CDX2	Caudal Type Homeobox 2	64	6.06
CEACAM3	Carcinoembryonic Antigen Related Cell Adhesion Molecule 3	58	1.63
CEACAM5	Carcinoembryonic Antigen Related Cell Adhesion Molecule 5	61	6.5
CEACAM6	Carcinoembryonic Antigen Related Cell Adhesion Molecule 6	61	4.72
CEACAM7	Carcinoembryonic Antigen Related Cell Adhesion Molecule 7	54	0.85
CEBPA	CCAAT/Enhancer Binding Protein Alpha	64	1.74
CEBPB	CCAAT/Enhancer Binding Protein Beta	62	3.72
CEBPE	CCAAT/Enhancer Binding Protein Epsilon	56	0.85
CELA1	Chymotrypsin Like Elastase Family Member 1	53	0.66
CEP68	Centrosomal Protein 68	51	0.51
CES1	Carboxylesterase 1	65	1.49
CETP	Cholesteryl Ester Transfer Protein	64	2.15
CFB	Complement Factor B	65	3.35
CFH	Complement Factor H	65	7.32
CFHR5	Complement Factor H Related 5	54	1.47
CFI	Complement Factor I	62	2.69
CFL2	Cofilin 2	62	2.85
CFLAR	CASP8 And FADD Like Apoptosis Regulator	66	3.95
CFP	Complement Factor Properdin	62	3.76
CFTR	Cystic Fibrosis Transmembrane Conductance Regulator	70	16.72
CGA	Glycoprotein Hormones, Alpha Polypeptide	65	4.6
CGB5	Chorionic Gonadotropin Beta Subunit 5	47	2.24
CHAT	Choline O-Acetyltransferase	68	6
CHD7	Chromodomain Helicase DNA Binding Protein 7	62	6.45
CHEK2	Checkpoint Kinase 2	76	4.4
CHGA	Chromogranin A	62	10.48

CHGB	Chromogranin B	60	3.51
CHI3L1	Chitinase 3 Like 1	61	11.89
CHIA	Chitinase, Acidic	55	5.04
CHIT1	Chitinase 1	62	6.27
CHKA	Choline Kinase Alpha	58	2.12
CHKB	Choline Kinase Beta	61	8.69
CHRFAM7A	CHRNA7 (Exons 5-10) And FAM7A (Exons A-E) Fusion	50	0.85
CHRM2	Cholinergic Receptor Muscarinic 2	68	1.9
CHRM3	Cholinergic Receptor Muscarinic 3	69	3.33
CHRNA4	Cholinergic Receptor Nicotinic Alpha 4 Subunit	68	4.13
CHRNA7	Cholinergic Receptor Nicotinic Alpha 7 Subunit	67	5.45
CHRNB2	Cholinergic Receptor Nicotinic Beta 2 Subunit	65	2
CHST14	Carbohydrate Sulfotransferase 14	57	1.77
CHST4	Carbohydrate Sulfotransferase 4	54	0.66
CHST8	Carbohydrate Sulfotransferase 8	58	10.39
CHUK	Conserved Helix-Loop-Helix Ubiquitous Kinase	72	7.77
CIB1	Calcium And Integrin Binding 1	57	0.85
CIDEC	Cell Death Inducing DFFA Like Effector C	58	2.01
CIITA	Class II Major Histocompatibility Complex Transactivator	62	12.27
CISH	Cytokine Inducible SH2 Containing Protein	62	1.51
CLC	Charcot-Leyden Crystal Galectin	56	1.41
CLCN1	Chloride Voltage-Gated Channel 1	60	3.33
CLCN7	Chloride Voltage-Gated Channel 7	61	6.23
CLDN1	Claudin 1	68	2.13
CLDN11	Claudin 11	57	3.13
CLDN18	Claudin 18	57	3.56
CLDN3	Claudin 3	58	0.85
CLDN4	Claudin 4	60	1.12
CLDN5	Claudin 5	60	1.38
CLDN8	Claudin 8	52	0.93
CLEC11A	C-Type Lectin Domain Containing 11A	56	2.88
CLEC12B	C-Type Lectin Domain Family 12 Member B	51	3.93
CLEC4M	C-Type Lectin Domain Family 4 Member M	58	1.32
CLEC7A	C-Type Lectin Domain Containing 7A	63	5.9
CLIP2	CAP-Gly Domain Containing Linker Protein 2	52	6.72
CLN3	CLN3, Battenin	60	1.1
CLOCK	Clock Circadian Regulator	62	1.34
CLTC	Clathrin Heavy Chain	64	10.09
CLU	Clusterin	66	1.69
CMA1	Chymase 1	62	4.11
CMKLR1	Chemerin Chemokine-Like Receptor 1	59	1.92
CNBP	CCHC-Type Zinc Finger Nucleic Acid Binding Protein	58	2.25
CNP	2',3'-Cyclic Nucleotide 3' Phosphodiesterase	59	2.6
CNR1	Cannabinoid Receptor 1	66	9.62
CNR2	Cannabinoid Receptor 2	64	7.43
CNTF	Ciliary Neurotrophic Factor	60	3.35
CNTN1	Contactin 1	62	1.25
CNTN2	Contactin 2	64	2.78
COG2	Component Of Oligomeric Golgi Complex 2	54	1.77
COL10A1	Collagen Type X Alpha 1 Chain	58	2.12
COL11A1	Collagen Type XI Alpha 1 Chain	59	10.44
COL11A2	Collagen Type XI Alpha 2 Chain	59	8.88
COL14A1	Collagen Type XIV Alpha 1 Chain	59	2.6
COL17A1	Collagen Type XVII Alpha 1 Chain	60	5.97
COL1A1	Collagen Type I Alpha 1 Chain	70	16.5
COL1A2	Collagen Type I Alpha 2 Chain	66	11.02
COL2A1	Collagen Type II Alpha 1 Chain	68	20.58
COL3A1	Collagen Type III Alpha 1 Chain	65	5.26
COL4A2	Collagen Type IV Alpha 2 Chain	62	1.69

COL4A3	Collagen Type IV Alpha 3 Chain	65	4.09
COL4A4	Collagen Type IV Alpha 4 Chain	58	1.74
COL4A5	Collagen Type IV Alpha 5 Chain	62	1.84
COL5A1	Collagen Type V Alpha 1 Chain	58	11.56
COL5A2	Collagen Type V Alpha 2 Chain	58	2.67
COL6A1	Collagen Type VI Alpha 1 Chain	61	6.53
COL6A2	Collagen Type VI Alpha 2 Chain	60	3.34
COL6A3	Collagen Type VI Alpha 3 Chain	60	3.34
COL7A1	Collagen Type VII Alpha 1 Chain	62	2.22
COL9A1	Collagen Type IX Alpha 1 Chain	60	12.05
COL9A2	Collagen Type IX Alpha 2 Chain	60	7.43
COL9A3	Collagen Type IX Alpha 3 Chain	59	8.31
COMMD1	Copper Metabolism Domain Containing 1	57	1.03
COMP	Cartilage Oligomeric Matrix Protein	68	20.44
COMT	Catechol-O-Methyltransferase	71	23.61
CORT	Cortistatin	54	1.34
COX5A	Cytochrome C Oxidase Subunit 5A	58	12.03
CP	Ceruloplasmin	67	10.12
CPA3	Carboxypeptidase A3	57	2.06
CPB2	Carboxypeptidase B2	65	2.46
CPD	Carboxypeptidase D	58	1.09
CPOX	Coproporphyrinogen Oxidase	61	8.06
CPQ	Carboxypeptidase Q	50	0.93
CPT1B	Carnitine Palmitoyltransferase 1B	62	3.34
CPT2	Carnitine Palmitoyltransferase 2	68	9.68
CR1	Complement C3b/C4b Receptor 1 (Knops Blood Group)	63	16.01
CR2	Complement C3d Receptor 2	62	21.84
CRABP1	Cellular Retinoic Acid Binding Protein 1	60	2.05
CREB1	CAMP Responsive Element Binding Protein 1	69	11.92
CREB3	CAMP Responsive Element Binding Protein 3	57	1.18
CREB3L3	CAMP Responsive Element Binding Protein 3 Like 3	54	2.2
CREB5	CAMP Responsive Element Binding Protein 5	54	0.73
CREBBP	CREB Binding Protein	71	1.78
CREM	CAMP Responsive Element Modulator	58	0.81
CRH	Corticotropin Releasing Hormone	61	11.77
CRHBP	Corticotropin Releasing Hormone Binding Protein	54	1.8
CRHR1	Corticotropin Releasing Hormone Receptor 1	66	2.9
CRHR2	Corticotropin Releasing Hormone Receptor 2	62	1.47
CRISP3	Cysteine Rich Secretory Protein 3	54	0.85
CRLF1	Cytokine Receptor Like Factor 1	58	7.15
CRLF2	Cytokine Receptor Like Factor 2	50	0.73
CRP	C-Reactive Protein	68	36.77
CRTC3	CREB Regulated Transcription Coactivator 3	55	0.57
CRYAA	Crystallin Alpha A	62	2.97
CRYAB	Crystallin Alpha B	64	4.9
CS	Citrate Synthase	63	0.85
CSE1L	Chromosome Segregation 1 Like	55	0.73
CSF1	Colony Stimulating Factor 1	65	13.85
CSF1R	Colony Stimulating Factor 1 Receptor	75	6.19
CSF2	Colony Stimulating Factor 2	65	16.77
CSF3	Colony Stimulating Factor 3	64	12.18
CSF3R	Colony Stimulating Factor 3 Receptor	67	1.03
CSK	C-Terminal Src Kinase	69	0.95
CSN1S1	Casein Alpha S1	50	1.63
CSN3	Casein Kappa	53	3.01
CST3	Cystatin C	64	4.52
CST7	Cystatin F	54	0.57
CTF1	Cardiotrophin 1	55	0.73
CTGF	Connective Tissue Growth Factor	68	16.75

CTLA4	Cytotoxic T-Lymphocyte Associated Protein 4	70	29.29
CTNNA1	Catenin Alpha 1	67	0.85
CTNNB1	Catenin Beta 1	75	25.85
CTNNBIP1	Catenin Beta Interacting Protein 1	58	0.81
CTNND1	Catenin Delta 1	62	1.51
CTRL	Chymotrypsin Like	58	1.08
CTSA	Cathepsin A	63	6.28
CTSB	Cathepsin B	72	6.56
CTSC	Cathepsin C	65	5.03
CTSD	Cathepsin D	75	2.46
CTSG	Cathepsin G	66	8.34
CTSH	Cathepsin H	66	1.1
CTSK	Cathepsin K	70	18.38
CTSL	Cathepsin L	65	6.12
CTSS	Cathepsin S	68	2.13
CTTN	Cortactin	62	3.28
CUL2	Cullin 2	58	1.12
CUX1	Cut Like Homeobox 1	58	3.19
CX3CL1	C-X3-C Motif Chemokine Ligand 1	60	2.92
CX3CR1	C-X3-C Motif Chemokine Receptor 1	63	9.58
CXADR	CXADR, Ig-Like Cell Adhesion Molecule	61	3.34
CXCL1	C-X-C Motif Chemokine Ligand 1	62	10.44
CXCL10	C-X-C Motif Chemokine Ligand 10	63	8.44
CXCL11	C-X-C Motif Chemokine Ligand 11	61	4.71
CXCL12	C-X-C Motif Chemokine Ligand 12	65	8.39
CXCL13	C-X-C Motif Chemokine Ligand 13	59	6.39
CXCL14	C-X-C Motif Chemokine Ligand 14	56	1.75
CXCL2	C-X-C Motif Chemokine Ligand 2	58	9.85
CXCL5	C-X-C Motif Chemokine Ligand 5	59	8.93
CXCL6	C-X-C Motif Chemokine Ligand 6	58	5.76
CXCL8	C-X-C Motif Chemokine Ligand 8	57	33.48
CXCL9	C-X-C Motif Chemokine Ligand 9	58	10.01
CXCR1	C-X-C Motif Chemokine Receptor 1	63	3.47
CXCR2	C-X-C Motif Chemokine Receptor 2	68	5.33
CXCR3	C-X-C Motif Chemokine Receptor 3	64	12.75
CXCR4	C-X-C Motif Chemokine Receptor 4	75	14
CXCR5	C-X-C Motif Chemokine Receptor 5	62	2.69
CYBB	Cytochrome B-245 Beta Chain	64	7.1
CYCS	Cytochrome C, Somatic	68	3.24
CYLD	CYLD Lysine 63 Deubiquitinase	67	1.19
CYP11B1	Cytochrome P450 Family 11 Subfamily B Member 1	62	1.51
CYP11B2	Cytochrome P450 Family 11 Subfamily B Member 2	65	1.31
CYP19A1	Cytochrome P450 Family 19 Subfamily A Member 1	70	7.92
CYP1A1	Cytochrome P450 Family 1 Subfamily A Member 1	68	4.16
CYP1A2	Cytochrome P450 Family 1 Subfamily A Member 2	68	7.3
CYP1B1	Cytochrome P450 Family 1 Subfamily B Member 1	68	1.46
CYP21A2	Cytochrome P450 Family 21 Subfamily A Member 2	62	4.63
CYP24A1	Cytochrome P450 Family 24 Subfamily A Member 1	64	1.03
CYP26C1	Cytochrome P450 Family 26 Subfamily C Member 1	57	2.05
CYP27A1	Cytochrome P450 Family 27 Subfamily A Member 1	63	1.48
CYP2A6	Cytochrome P450 Family 2 Subfamily A Member 6	65	2.03
CYP2B6	Cytochrome P450 Family 2 Subfamily B Member 6	69	2.44
CYP2C19	Cytochrome P450 Family 2 Subfamily C Member 19	67	7.08
CYP2C8	Cytochrome P450 Family 2 Subfamily C Member 8	69	3.32
CYP2C9	Cytochrome P450 Family 2 Subfamily C Member 9	69	5.81
CYP2D6	Cytochrome P450 Family 2 Subfamily D Member 6	73	8.13
CYP2E1	Cytochrome P450 Family 2 Subfamily E Member 1	66	2.17
CYP3A4	Cytochrome P450 Family 3 Subfamily A Member 4	74	5.07
CYP3A5	Cytochrome P450 Family 3 Subfamily A Member 5	68	6

CYP4F2	Cytochrome P450 Family 4 Subfamily F Member 2	62	1.51
CYP4F22	Cytochrome P450 Family 4 Subfamily F Member 22	54	2.21
CYP4F3	Cytochrome P450 Family 4 Subfamily F Member 3	60	3.15
CYP7A1	Cytochrome P450 Family 7 Subfamily A Member 1	60	2.03
CYP7B1	Cytochrome P450 Family 7 Subfamily B Member 1	61	3.51
CYR61	Cysteine Rich Angiogenic Inducer 61	60	0.95
CYSLTR1	Cysteinyl Leukotriene Receptor 1	66	4.91
CYSLTR2	Cysteinyl Leukotriene Receptor 2	66	12.58
DAB2IP	DAB2 Interacting Protein	54	1.79
DAG1	Dystroglycan 1	64	4.16
DAO	D-Amino Acid Oxidase	61	2.41
DAPK1	Death Associated Protein Kinase 1	66	1.52
DBH	Dopamine Beta-Hydroxylase	73	6.25
DCC	DCC Netrin 1 Receptor	63	2.17
DCD	Dermcidin	52	0.57
DCLRE1C	DNA Cross-Link Repair 1C	59	2.48
DCN	Decorin	66	3.82
DCT	Dopachrome Tautomerase	59	0.57
DCX	Doublecortin	62	1.32
DDAH2	Dimethylarginine Dimethylaminohydrolase 2	61	0.85
DDB2	Damage Specific DNA Binding Protein 2	62	5.01
DDIT3	DNA Damage Inducible Transcript 3	66	13.06
DDR2	Discoidin Domain Receptor Tyrosine Kinase 2	75	1.93
DDX39B	DExD-Box Helicase 39B	54	0.66
DDX41	DEAD-Box Helicase 41	59	3.67
DDX58	DExD/H-Box Helicase 58	67	5
DECR1	2,4-Dienoyl-CoA Reductase 1	59	2.26
DEFA1	Defensin Alpha 1	57	0.95
DEFA3	Defensin Alpha 3	52	1.03
DEFB1	Defensin Beta 1	54	4.61
DEFB4A	Defensin Beta 4A	51	6.12
DEK	DEK Proto-Oncogene	58	3.41
DES	Desmin	68	10.47
DHDH	Dihydrodiol Dehydrogenase	54	0.73
DHFR	Dihydrofolate Reductase	69	1.11
DHODH	Dihydroorotate Dehydrogenase (Quinone)	61	0.57
DIABLO	Diablo IAP-Binding Mitochondrial Protein	67	1.19
DIO3	Iodothyronine Deiodinase 3	54	0.57
DKC1	Dyskerin Pseudouridine Synthase 1	65	3.45
DLAT	Dihydrolipoamide S-Acetyltransferase	65	3.96
DLG2	Discs Large MAGUK Scaffold Protein 2	58	7.56
DLG4	Discs Large MAGUK Scaffold Protein 4	65	1.61
DLG5	Discs Large MAGUK Scaffold Protein 5	54	7.82
DLX4	Distal-Less Homeobox 4	56	1.19
DMBT1	Deleted In Malignant Brain Tumors 1	60	4.32
DMD	Dystrophin	63	13.49
DNAH8	Dynein Axonemal Heavy Chain 8	52	2.83
DNAI1	Dynein Axonemal Intermediate Chain 1	55	3.8
DNAJC17	DnaJ Heat Shock Protein Family (Hsp40) Member C17	51	2.6
DNASE1	Deoxyribonuclease 1	58	8.33
DNASE1L3	Deoxyribonuclease 1 Like 3	57	17.7
DNMT1	DNA Methyltransferase 1	74	3.88
DNMT3B	DNA Methyltransferase 3 Beta	69	2.35
DNTT	DNA Nucleotidylexotransferase	60	1.64
DOCK8	Dedicator Of Cytokinesis 8	59	5.54
DPM1	Dolichyl-Phosphate Mannosyltransferase Subunit 1, Catalytic	59	1.04
DPP4	Dipeptidyl Peptidase 4	73	0.98
DPT	Dermatopontin	54	2.9
DPYD	Dihydropyrimidine Dehydrogenase	70	5.23

DPYSL2	Dihydropyrimidinase Like 2	62	2.31
DRD2	Dopamine Receptor D2	70	4.64
DRGX	Dorsal Root Ganglia Homeobox	43	1.11
DSC2	Desmocollin 2	65	3.13
DSG1	Desmoglein 1	60	8.72
DSG2	Desmoglein 2	63	5.01
DSG3	Desmoglein 3	57	2.6
DSP	Desmoplakin	68	3.76
DSPP	Dentin Sialophosphoprotein	51	2.46
DUSP1	Dual Specificity Phosphatase 1	66	1.02
DUSP2	Dual Specificity Phosphatase 2	58	0.51
DUSP6	Dual Specificity Phosphatase 6	69	1.56
DYNC2H1	Dynein Cytoplasmic 2 Heavy Chain 1	54	2.36
DYNLT1	Dynein Light Chain Tctex-Type 1	55	4.85
DYSF	Dysferlin	59	6.04
E2F1	E2F Transcription Factor 1	62	1.46
EBI3	Epstein-Barr Virus Induced 3	58	2.13
ECE1	Endothelin Converting Enzyme 1	65	6.01
ECM1	Extracellular Matrix Protein 1	59	4.87
EDA	Ectodysplasin A	58	6.12
EDN1	Endothelin 1	67	13.12
EDN2	Endothelin 2	61	1.5
EDN3	Endothelin 3	63	5.09
EDNRA	Endothelin Receptor Type A	70	4.9
EDNRB	Endothelin Receptor Type B	70	8.22
EFNB2	Ephrin B2	64	0.66
EGF	Epidermal Growth Factor	73	8.11
EGFR	Epidermal Growth Factor Receptor	79	31.78
EGOT	Eosinophil Granule Ontogeny Transcript (Non-Protein Coding)	26	3.28
EGR1	Early Growth Response 1	66	10.12
EGR2	Early Growth Response 2	64	4.28
EIF1AX	Eukaryotic Translation Initiation Factor 1A, X-Linked	58	2.05
EIF2AK2	Eukaryotic Translation Initiation Factor 2 Alpha Kinase 2	65	5.21
EIF2S1	Eukaryotic Translation Initiation Factor 2 Subunit Alpha	65	1.16
EIF4E	Eukaryotic Translation Initiation Factor 4E	70	0.88
EIF4EBP1	Eukaryotic Translation Initiation Factor 4E Binding Protein 1	70	1.11
EIF4G1	Eukaryotic Translation Initiation Factor 4 Gamma 1	65	0.81
ELANE	Elastase, Neutrophil Expressed	68	31.8
ELAVL1	ELAV Like RNA Binding Protein 1	59	0.88
ELF2	E74 Like ETS Transcription Factor 2	51	3.28
ELF3	E74 Like ETS Transcription Factor 3	55	0.86
ELN	Elastin	62	17.95
ELP1	Elongator Complex Protein 1	46	12.64
EMD	Emerin	62	6.19
EML4	Echinoderm Microtubule Associated Protein Like 4	54	5.93
EMSY	EMSY, BRCA2 Interacting Transcriptional Repressor	38	0.73
ENG	Endoglin	64	10.88
ENO1	Enolase 1	68	6.77
ENO2	Enolase 2	70	2.99
ENPP1	Ectonucleotide Pyrophosphatase/Phosphodiesterase 1	66	8.94
ENPP2	Ectonucleotide Pyrophosphatase/Phosphodiesterase 2	66	2.25
ENPP3	Ectonucleotide Pyrophosphatase/Phosphodiesterase 3	61	0.57
ENTPD1	Ectonucleoside Triphosphate Diphosphohydrolase 1	66	3.34
EP300	E1A Binding Protein P300	72	3.65
EPAS1	Endothelial PAS Domain Protein 1	66	1.46
EPCAM	Epithelial Cell Adhesion Molecule	64	12.37
EPG5	Ectopic P-Granules Autophagy Protein 5 Homolog	49	1.77
EPHA3	EPH Receptor A3	65	0.57
EPHB2	EPH Receptor B2	70	2.93

EPHB4	EPH Receptor B4	71	0.66
EPHX1	Epoxide Hydrolase 1	64	3.69
EPHX2	Epoxide Hydrolase 2	65	2.24
EPO	Erythropoietin	63	4.73
EPOR	Erythropoietin Receptor	69	3.83
EPRS	Glutamyl-Prolyl-TRNA Synthetase	62	2.6
EPSTI1	Epithelial Stromal Interaction 1	49	3.51
EPX	Eosinophil Peroxidase	60	8.74
ERAP1	Endoplasmic Reticulum Aminopeptidase 1	62	16.78
ERAS	ES Cell Expressed Ras	50	2.65
ERBB2	Erb-B2 Receptor Tyrosine Kinase 2	79	22.03
ERBB3	Erb-B2 Receptor Tyrosine Kinase 3	76	4.28
ERCC2	ERCC Excision Repair 2, TFIIH Core Complex Helicase Subunit	66	6.21
ERCC3	ERCC Excision Repair 3, TFIIH Core Complex Helicase Subunit	68	5.01
ERCC4	ERCC Excision Repair 4, Endonuclease Catalytic Subunit	61	5.01
ERCC5	ERCC Excision Repair 5, Endonuclease	62	5.65
ERCC6	ERCC Excision Repair 6, Chromatin Remodeling Factor	60	4.18
EREG	Epiregulin	62	0.81
ERF	ETS2 Repressor Factor	55	3.65
ERN1	Endoplasmic Reticulum To Nucleus Signaling 1	67	0.93
ERVK-6	Endogenous Retrovirus Group K Member 6, Envelope	24	0.68
ERVW-1	Endogenous Retrovirus Group W Member 1, Envelope	45	4.63
ESD	Esterase D	61	1.79
ESR1	Estrogen Receptor 1	79	31.52
ESR2	Estrogen Receptor 2	69	4.02
ETS1	ETS Proto-Oncogene 1, Transcription Factor	69	1.83
ETS2	ETS Proto-Oncogene 2, Transcription Factor	66	1.03
EWSR1	EWS RNA Binding Protein 1	58	11.55
EXO1	Exonuclease 1	62	1.03
EXOSC9	Exosome Component 9	55	3.01
EZH2	Enhancer Of Zeste 2 Polycomb Repressive Complex 2 Subunit	75	1.91
EZR	Ezrin	65	3.88
F10	Coagulation Factor X	70	3.72
F12	Coagulation Factor XII	67	4.34
F13A1	Coagulation Factor XIII A Chain	64	8.03
F13B	Coagulation Factor XIII B Chain	55	4.11
F2	Coagulation Factor II, Thrombin	70	27.35
F2R	Coagulation Factor II Thrombin Receptor	68	5.8
F2RL1	F2R Like Trypsin Receptor 1	66	7.02
F2RL3	F2R Like Thrombin/Trypsin Receptor 3	63	2.8
F3	Coagulation Factor III, Tissue Factor	66	13.2
F5	Coagulation Factor V	63	28.59
F7	Coagulation Factor VII	67	9.64
F8	Coagulation Factor VIII	66	6.03
F9	Coagulation Factor IX	66	3.52
FA2H	Fatty Acid 2-Hydroxylase	61	3.8
FAAH	Fatty Acid Amide Hydrolase	67	3.72
FABP12	Fatty Acid Binding Protein 12	45	1.74
FABP2	Fatty Acid Binding Protein 2	62	3.48
FABP4	Fatty Acid Binding Protein 4	66	1.67
FADS1	Fatty Acid Desaturase 1	62	1.19
FAM20C	FAM20C, Golgi Associated Secretory Pathway Kinase	58	2.8
FANCA	Fanconi Anemia Complementation Group A	66	0.66
FANCD2	Fanconi Anemia Complementation Group D2	60	0.81
FAS	Fas Cell Surface Death Receptor	70	26.18
FASLG	Fas Ligand	70	16.54
FASN	Fatty Acid Synthase	71	1.44
FBL	Fibrillarin	61	2.8
FBLN2	Fibulin 2	60	1.03

FBLN5	Fibulin 5	63	0.99
FBN2	Fibrillin 2	59	3.13
FCER1A	Fc Fragment Of IgE Receptor Ia	63	4.11
FCER2	Fc Fragment Of IgE Receptor II	66	6.56
FCGR1A	Fc Fragment Of IgG Receptor Ia	62	4.23
FCGR2A	Fc Fragment Of IgG Receptor IIa	64	9.46
FCGR2B	Fc Fragment Of IgG Receptor IIb	66	9.34
FCGR3A	Fc Fragment Of IgG Receptor IIIa	62	8.67
FCGR3B	Fc Fragment Of IgG Receptor IIIb	62	2.81
FCN1	Ficolin 1	59	3.67
FCN2	Ficolin 2	59	4.11
FCN3	Ficolin 3	59	4.85
FCRL3	Fc Receptor Like 3	54	5.63
FCRL4	Fc Receptor Like 4	51	3.13
FECH	Ferrochelatase	64	4.64
FERMT1	Fermitin Family Member 1	56	4.03
FFAR1	Free Fatty Acid Receptor 1	62	0.97
FGA	Fibrinogen Alpha Chain	66	4.47
FGB	Fibrinogen Beta Chain	65	2.44
FGF1	Fibroblast Growth Factor 1	70	6.72
FGF14	Fibroblast Growth Factor 14	60	1.09
FGF2	Fibroblast Growth Factor 2	70	11.34
FGF20	Fibroblast Growth Factor 20	59	2.97
FGF23	Fibroblast Growth Factor 23	63	6.41
FGF3	Fibroblast Growth Factor 3	64	2.36
FGF4	Fibroblast Growth Factor 4	63	3.13
FGF7	Fibroblast Growth Factor 7	62	4.14
FGF9	Fibroblast Growth Factor 9	64	0.81
FGFR1	Fibroblast Growth Factor Receptor 1	80	5.37
FGFR1OP	FGFR1 Oncogene Partner	52	3.76
FGFR2	Fibroblast Growth Factor Receptor 2	79	14.32
FGFR3	Fibroblast Growth Factor Receptor 3	80	14.96
FGG	Fibrinogen Gamma Chain	65	1.14
FGL2	Fibrinogen Like 2	58	0.85
FHIT	Fragile Histidine Triad	62	4.08
FHL1	Four And A Half LIM Domains 1	63	8.35
FIP1L1	Factor Interacting With PAPOLA And CPSF1	52	7.08
FKBP5	FK506 Binding Protein 5	63	1.14
FLG	Filaggrin	55	16.33
FLI1	Fli-1 Proto-Oncogene, ETS Transcription Factor	69	3.06
FLII	FLII, Actin Remodeling Protein	58	6.33
FLNA	Filamin A	66	12.93
FLNC	Filamin C	62	9.84
FLT1	Fms Related Tyrosine Kinase 1	75	8.28
FLT3	Fms Related Tyrosine Kinase 3	75	2.88
FLT3LG	Fms Related Tyrosine Kinase 3 Ligand	60	0.66
FLT4	Fms Related Tyrosine Kinase 4	75	4.99
FMR1	Fragile X Mental Retardation 1	62	7.85
FN1	Fibronectin 1	72	3.34
FOLR2	Folate Receptor Beta	57	4.5
FOS	Fos Proto-Oncogene, AP-1 Transcription Factor Subunit	71	15.36
FOSB	FosB Proto-Oncogene, AP-1 Transcription Factor Subunit	58	0.66
FOSL1	FOS Like 1, AP-1 Transcription Factor Subunit	61	0.78
FOXC2	Forkhead Box C2	58	4.54
FOXF1	Forkhead Box F1	55	1.95
FOXM1	Forkhead Box M1	61	0.85
FOXO1	Forkhead Box O1	64	1.26
FOXO3	Forkhead Box O3	60	0.88
FOXP1	Forkhead Box P1	62	6.74

FOXP2	Forkhead Box P2	58	1.38
FOXP3	Forkhead Box P3	67	12.38
FPR2	Formyl Peptide Receptor 2	67	2.66
FSCN1	Fascin Actin-Bundling Protein 1	62	3.82
FSTL1	Follistatin Like 1	61	5.57
FTH1	Ferritin Heavy Chain 1	68	1.32
FTL	Ferritin Light Chain	66	2.03
FURIN	Furin, Paired Basic Amino Acid Cleaving Enzyme	70	2.8
FUS	FUS RNA Binding Protein	62	11.31
FUT2	Fucosyltransferase 2	61	5.68
FUT3	Fucosyltransferase 3 (Lewis Blood Group)	61	5.83
FUT4	Fucosyltransferase 4	54	0.73
FYN	FYN Proto-Oncogene, Src Family Tyrosine Kinase	71	2.79
G6PC	Glucose-6-Phosphatase Catalytic Subunit	62	2.24
G6PC3	Glucose-6-Phosphatase Catalytic Subunit 3	57	0.94
G6PD	Glucose-6-Phosphate Dehydrogenase	68	15.92
GABPA	GA Binding Protein Transcription Factor Alpha Subunit	56	1.28
GAD1	Glutamate Decarboxylase 1	70	4.81
GAD2	Glutamate Decarboxylase 2	66	1.5
GADD45A	Growth Arrest And DNA Damage Inducible Alpha	67	2
GAL	Galanin And GMAP Prepropeptide	62	3.74
GALC	Galactosylceramidase	63	1.19
GALE	UDP-Galactose-4-Epimerase	63	4.28
GALNS	Galactosamine (N-Acetyl)-6-Sulfatase	62	0.81
GALNT3	Polypeptide N-Acetylgalactosaminyltransferase 3	62	11.22
GALR2	Galanin Receptor 2	62	1.55
GALT	Galactose-1-Phosphate Uridylyltransferase	62	1.45
GAP43	Growth Associated Protein 43	60	1.88
GAPDH	Glyceraldehyde-3-Phosphate Dehydrogenase	70	2.12
GARS	Glycyl-TRNA Synthetase	64	6.53
GAS6	Growth Arrest Specific 6	63	2.46
GAST	Gastrin	57	8.84
GATA1	GATA Binding Protein 1	65	6.86
GATA2	GATA Binding Protein 2	66	6.83
GATA3	GATA Binding Protein 3	67	11.14
GATA4	GATA Binding Protein 4	67	2.17
GATA6	GATA Binding Protein 6	63	1.49
GBA	Glucosylceramidase Beta	68	9.15
GBP1	Guanylate Binding Protein 1	60	1.02
GC	GC, Vitamin D Binding Protein	62	2.27
GCG	Glucagon	61	2.75
GCH1	GTP Cyclohydrolase 1	63	6.51
GCKR	Glucokinase Regulator	57	1.03
GCLC	Glutamate-Cysteine Ligase Catalytic Subunit	62	1.64
GCLM	Glutamate-Cysteine Ligase Modifier Subunit	57	1.57
GDF15	Growth Differentiation Factor 15	62	2.69
GDF2	Growth Differentiation Factor 2	58	1.08
GDF5	Growth Differentiation Factor 5	66	4.22
GDNF	Glial Cell Derived Neurotrophic Factor	68	23.69
GFAP	Glial Fibrillary Acidic Protein	69	11.88
GFI1	Growth Factor Independent 1 Transcriptional Repressor	62	2.46
GFRA1	GDNF Family Receptor Alpha 1	62	4.79
GGT1	Gamma-Glutamyltransferase 1	66	8.91
GGT5	Gamma-Glutamyltransferase 5	57	0.66
GH1	Growth Hormone 1	65	12.03
GHR	Growth Hormone Receptor	66	2.01
GHRL	Ghrelin And Obestatin Prepropeptide	65	9.9
GHSR	Growth Hormone Secretagogue Receptor	67	3.43
GIF	Gastric Intrinsic Factor	62	3.05

GIMAP2	GTPase, IMAP Family Member 2	51	3.93
GIMAP4	GTPase, IMAP Family Member 4	52	3.93
GIP	Gastric Inhibitory Polypeptide	61	3.93
GIPR	Gastric Inhibitory Polypeptide Receptor	62	2.6
GJA1	Gap Junction Protein Alpha 1	71	4.51
GJA4	Gap Junction Protein Alpha 4	61	1.34
GJB1	Gap Junction Protein Beta 1	66	6.93
GJB2	Gap Junction Protein Beta 2	65	11.99
GJB3	Gap Junction Protein Beta 3	62	1.77
GJB4	Gap Junction Protein Beta 4	59	1.77
GJB6	Gap Junction Protein Beta 6	62	5.85
GJC2	Gap Junction Protein Gamma 2	56	4.29
GK	Glycerol Kinase	65	0.57
GKN1	Gastrokine 1	54	0.57
GLA	Galactosidase Alpha	69	8.83
GLI1	GLI Family Zinc Finger 1	63	1.46
GLI3	GLI Family Zinc Finger 3	67	1.19
GLP1R	Glucagon Like Peptide 1 Receptor	66	0.73
GLRA1	Glycine Receptor Alpha 1	63	2.7
GLRA3	Glycine Receptor Alpha 3	57	2.48
GLRB	Glycine Receptor Beta	66	2.42
GLUL	Glutamate-Ammonia Ligase	67	2.89
GMPPB	GDP-Mannose Pyrophosphorylase B	61	0.91
GNA11	G Protein Subunit Alpha 11	67	12.6
GNAQ	G Protein Subunit Alpha Q	69	8.79
GNAS	GNAS Complex Locus	68	18.08
GNB1L	G Protein Subunit Beta 1 Like	49	2.8
GNB3	G Protein Subunit Beta 3	66	2.94
GNE	Glucosamine (UDP-N-Acetyl)-2-Epimerase/N-Acetylmannosamine Kinase	62	13.92
GNLY	Granulysin	55	0.85
GNPTAB	N-Acetylglucosamine-1-Phosphate Transferase Alpha And Beta Subunits	57	3.2
GNRH1	Gonadotropin Releasing Hormone 1	61	6.04
GP1BB	Glycoprotein Ib Platelet Beta Subunit	58	5.16
GPBAR1	G Protein-Coupled Bile Acid Receptor 1	58	2.71
GPER1	G Protein-Coupled Estrogen Receptor 1	53	2.07
GPI	Glucose-6-Phosphate Isomerase	67	1.48
GPIHBP1	Glycosylphosphatidylinositol High Density Lipoprotein Binding Protein 1	50	6.72
GPR101	G Protein-Coupled Receptor 101	54	5.8
GPR35	G Protein-Coupled Receptor 35	61	4.92
GPR4	G Protein-Coupled Receptor 4	56	0.57
GPR55	G Protein-Coupled Receptor 55	58	3.33
GPR65	G Protein-Coupled Receptor 65	58	1.14
GPR68	G Protein-Coupled Receptor 68	58	1.21
GPRC5B	G Protein-Coupled Receptor Class C Group 5 Member B	54	1.26
GPRC6A	G Protein-Coupled Receptor Class C Group 6 Member A	54	0.97
GPT	Glutamic--Pyruvic Transaminase	59	8.53
GPX1	Glutathione Peroxidase 1	66	3.23
GPX2	Glutathione Peroxidase 2	63	0.73
GPX3	Glutathione Peroxidase 3	64	1.42
GRB2	Growth Factor Receptor Bound Protein 2	71	3.41
GRIA1	Glutamate Ionotropic Receptor AMPA Type Subunit 1	68	2.91
GRIA2	Glutamate Ionotropic Receptor AMPA Type Subunit 2	69	2.73
GRIA3	Glutamate Ionotropic Receptor AMPA Type Subunit 3	69	1.64
GRIN1	Glutamate Ionotropic Receptor NMDA Type Subunit 1	70	3.55
GRK2	G Protein-Coupled Receptor Kinase 2	51	1.97
GRN	Granulin Precursor	66	2.93
GRP	Gastrin Releasing Peptide	59	2.96
GSK3B	Glycogen Synthase Kinase 3 Beta	72	2.29
GSN	Gelsolin	70	5.91

GSR	Glutathione-Disulfide Reductase	70	4.79
GSTA1	Glutathione S-Transferase Alpha 1	61	0.57
GSTA4	Glutathione S-Transferase Alpha 4	60	0.73
GSTM1	Glutathione S-Transferase Mu 1	61	11.54
GSTP1	Glutathione S-Transferase Pi 1	72	8.5
GSTT1	Glutathione S-Transferase Theta 1	49	5.41
GTF2I	General Transcription Factor IIi	58	7.16
GTF2IRD1	GTF2I Repeat Domain Containing 1	57	6.96
GUCY1A3	Guanylate Cyclase 1 Soluble Subunit Alpha	63	4.72
GUCY2C	Guanylate Cyclase 2C	62	8.84
GUCY2D	Guanylate Cyclase 2D, Retinal	59	0.46
GUSB	Glucuronidase Beta	66	5.27
GZMA	Granzyme A	62	0.88
GZMB	Granzyme B	67	6.04
GZMH	Granzyme H	58	0.57
H2AFX	H2A Histone Family Member X	64	0.85
H6PD	Hexose-6-Phosphate Dehydrogenase/Glucose 1-Dehydrogenase	58	1.92
HABP2	Hyaluronan Binding Protein 2	62	2.88
HADHA	Hydroxyacyl-CoA Dehydrogenase, Alpha Subunit	66	4.08
HAMP	Hepcidin Antimicrobial Peptide	57	3.04
HAPLN1	Hyaluronan And Proteoglycan Link Protein 1	58	3.83
HARS	Histidyl-TRNA Synthetase	66	6.21
HAS1	Hyaluronan Synthase 1	56	1.49
HAVCR1	Hepatitis A Virus Cellular Receptor 1	62	4.29
HAVCR2	Hepatitis A Virus Cellular Receptor 2	61	1.69
HBA1	Hemoglobin Subunit Alpha 1	60	4.89
HBA2	Hemoglobin Subunit Alpha 2	58	4.34
HBB	Hemoglobin Subunit Beta	66	11.94
HBEGF	Heparin Binding EGF Like Growth Factor	62	3.4
HBG2	Hemoglobin Subunit Gamma 2	59	5.27
HCK	HCK Proto-Oncogene, Src Family Tyrosine Kinase	74	4.06
HCRT	Hypocretin Neuropeptide Precursor	62	8.3
HCRTR2	Hypocretin Receptor 2	65	1.54
HDAC1	Histone Deacetylase 1	73	1.9
HDAC2	Histone Deacetylase 2	72	1.24
HDAC4	Histone Deacetylase 4	70	3.17
HDAC6	Histone Deacetylase 6	70	0.85
HDAC9	Histone Deacetylase 9	64	2.22
HDC	Histidine Decarboxylase	62	2.76
HERC2	HECT And RLD Domain Containing E3 Ubiquitin Protein Ligase 2	59	1.75
HFE	Hemochromatosis	61	6.83
HGD	Homogentisate 1,2-Dioxygenase	61	6.95
HGF	Hepatocyte Growth Factor	75	10.94
HIF1A	Hypoxia Inducible Factor 1 Alpha Subunit	74	3.96
HIRA	Histone Cell Cycle Regulator	58	4.79
HIST2H2BE	Histone Cluster 2 H2B Family Member E	56	2.36
HK1	Hexokinase 1	71	2.57
HK2	Hexokinase 2	67	0.85
HLA-A	Major Histocompatibility Complex, Class I, A	66	27.02
HLA-B	Major Histocompatibility Complex, Class I, B	65	41.83
HLA-C	Major Histocompatibility Complex, Class I, C	63	18.88
HLA-DMA	Major Histocompatibility Complex, Class II, DM Alpha	58	4.99
HLA-DMB	Major Histocompatibility Complex, Class II, DM Beta	58	4.48
HLA-DPB1	Major Histocompatibility Complex, Class II, DP Beta 1	60	23.92
HLA-DQA1	Major Histocompatibility Complex, Class II, DQ Alpha 1	58	19.34
HLA-DQB1	Major Histocompatibility Complex, Class II, DQ Beta 1	62	29.84
HLA-DQB2	Major Histocompatibility Complex, Class II, DQ Beta 2	52	2.9
HLA-DRA	Major Histocompatibility Complex, Class II, DR Alpha	64	5.21
HLA-DRB1	Major Histocompatibility Complex, Class II, DR Beta 1	63	48.4

HLA-DRB4	Major Histocompatibility Complex, Class II, DR Beta 4	41	0.72
HLA-DRB5	Major Histocompatibility Complex, Class II, DR Beta 5	53	5.07
HLA-G	Major Histocompatibility Complex, Class I, G	63	9.77
HMGA1	High Mobility Group AT-Hook 1	63	1.11
HMGA2	High Mobility Group AT-Hook 2	60	4.88
HMGB1	High Mobility Group Box 1	64	12.99
HMGB2	High Mobility Group Box 2	58	4.39
HMGCR	3-Hydroxy-3-Methylglutaryl-CoA Reductase	65	2.88
HMOX1	Heme Oxygenase 1	74	7.67
HMOX2	Heme Oxygenase 2	68	1.39
HNF1B	HNF1 Homeobox B	62	3.67
HNF4A	Hepatocyte Nuclear Factor 4 Alpha	71	3.76
HNMT	Histamine N-Methyltransferase	62	9.25
HNRNPA1	Heterogeneous Nuclear Ribonucleoprotein A1	64	7.16
HNRNPA2B1	Heterogeneous Nuclear Ribonucleoprotein A2/B1	62	2.08
HNRNPK	Heterogeneous Nuclear Ribonucleoprotein K	61	5.93
HOGA1	4-Hydroxy-2-Oxoglutarate Aldolase 1	52	14.27
HP	Haptoglobin	63	15.97
HPGD	Hydroxyprostaglandin Dehydrogenase 15-(NAD)	66	13.58
HPRT1	Hypoxanthine Phosphoribosyltransferase 1	66	9.4
HPS1	HPS1, Biogenesis Of Lysosomal Organelles Complex 3 Subunit 1	55	6.28
HPSE	Heparanase	65	2.28
HRAS	HRas Proto-Oncogene, GTPase	75	12.42
HRH1	Histamine Receptor H1	67	7.66
HRH2	Histamine Receptor H2	66	10.18
HRH4	Histamine Receptor H4	62	1.66
HSD11B1	Hydroxysteroid 11-Beta Dehydrogenase 1	70	4.96
HSD11B2	Hydroxysteroid 11-Beta Dehydrogenase 2	63	4.45
HSD3B1	Hydroxy-Delta-5-Steroid Dehydrogenase, 3 Beta- And Steroid Delta-Isomerase 1	60	0.88
HSD3B2	Hydroxy-Delta-5-Steroid Dehydrogenase, 3 Beta- And Steroid Delta-Isomerase 2	63	2.44
HSD3B7	Hydroxy-Delta-5-Steroid Dehydrogenase, 3 Beta- And Steroid Delta-Isomerase 7	58	1.47
HSF1	Heat Shock Transcription Factor 1	65	1.49
HSF4	Heat Shock Transcription Factor 4	56	5.57
HSP90AA1	Heat Shock Protein 90 Alpha Family Class A Member 1	70	3.41
HSP90B1	Heat Shock Protein 90 Beta Family Member 1	66	0.85
HSPA1A	Heat Shock Protein Family A (Hsp70) Member 1A	62	3.47
HSPA1B	Heat Shock Protein Family A (Hsp70) Member 1B	55	0.8
HSPA2	Heat Shock Protein Family A (Hsp70) Member 2	62	3.35
HSPA4	Heat Shock Protein Family A (Hsp70) Member 4	61	7.19
HSPA5	Heat Shock Protein Family A (Hsp70) Member 5	66	1.89
HSPA8	Heat Shock Protein Family A (Hsp70) Member 8	68	5.48
HSPB1	Heat Shock Protein Family B (Small) Member 1	71	5.84
HSPD1	Heat Shock Protein Family D (Hsp60) Member 1	67	12.26
HSPE1	Heat Shock Protein Family E (Hsp10) Member 1	58	0.88
HSPG2	Heparan Sulfate Proteoglycan 2	66	8.77
HTN3	Histatin 3	50	0.73
HTR1A	5-Hydroxytryptamine Receptor 1A	65	12.87
HTR2A	5-Hydroxytryptamine Receptor 2A	66	9.69
HTR2B	5-Hydroxytryptamine Receptor 2B	64	2.54
HTR2C	5-Hydroxytryptamine Receptor 2C	69	2.03
HTR3A	5-Hydroxytryptamine Receptor 3A	65	7.91
HTRA1	HtrA Serine Peptidase 1	61	7.68
HYAL1	Hyaluronoglucosaminidase 1	64	2.41
HYDIN	HYDIN, Axonemal Central Pair Apparatus Protein	49	1.74
IAPP	Islet Amyloid Polypeptide	56	2.13
IARS	Isoleucyl-TRNA Synthetase	63	4.42
IBD11	Inflammatory Bowel Disease 11	4	7.19
ICAM1	Intercellular Adhesion Molecule 1	72	31.13
ICAM3	Intercellular Adhesion Molecule 3	60	1.28

ICOS	Inducible T-Cell Costimulator	64	5.61
ICOSLG	Inducible T-Cell Costimulator Ligand	56	5.28
IDH1	Isocitrate Dehydrogenase (NADP(+)) 1, Cytosolic	75	3.53
IDH2	Isocitrate Dehydrogenase (NADP(+)) 2, Mitochondrial	73	8.19
IDO1	Indoleamine 2,3-Dioxygenase 1	65	7.46
IDS	Iduronate 2-Sulfatase	66	3.29
IFI16	Interferon Gamma Inducible Protein 16	59	1.97
IFI27	Interferon Alpha Inducible Protein 27	54	1.46
IFI35	Interferon Induced Protein 35	53	2.26
IFIH1	Interferon Induced With Helicase C Domain 1	68	15.52
IFITM1	Interferon Induced Transmembrane Protein 1	60	1.25
IFITM2	Interferon Induced Transmembrane Protein 2	53	3.17
IFITM3	Interferon Induced Transmembrane Protein 3	58	3.51
IFNA1	Interferon Alpha 1	58	11.69
IFNA2	Interferon Alpha 2	59	9.6
IFNAR1	Interferon Alpha And Beta Receptor Subunit 1	66	4.91
IFNAR2	Interferon Alpha And Beta Receptor Subunit 2	66	1.71
IFNB1	Interferon Beta 1	62	9.51
IFNG	Interferon Gamma	69	52.76
IFNGR1	Interferon Gamma Receptor 1	68	9.5
IFNGR2	Interferon Gamma Receptor 2	64	3.95
IFNL3	Interferon Lambda 3	50	4.63
IFT172	Intraflagellar Transport 172	52	2.7
IGES	Immunoglobulin E Concentration, Serum	10	4.83
IGF1	Insulin Like Growth Factor 1	70	11.22
IGF1R	Insulin Like Growth Factor 1 Receptor	79	5.59
IGF2	Insulin Like Growth Factor 2	67	6.53
IGF2R	Insulin Like Growth Factor 2 Receptor	63	2.92
IGFBP1	Insulin Like Growth Factor Binding Protein 1	62	1.55
IGFBP2	Insulin Like Growth Factor Binding Protein 2	62	1.69
IGFBP3	Insulin Like Growth Factor Binding Protein 3	66	9.12
IGFBP4	Insulin Like Growth Factor Binding Protein 4	62	1.1
IGFL1	IGF Like Family Member 1	44	0.57
IGFL2	IGF Like Family Member 2	45	0.57
IGFL3	IGF Like Family Member 3	47	0.85
IGFLR1	IGF Like Family Receptor 1	50	0.57
IGH	Immunoglobulin Heavy Locus	25	6.64
IGHE	Immunoglobulin Heavy Constant Epsilon	38	8.04
IGHG1	Immunoglobulin Heavy Constant Gamma 1 (G1m Marker)	45	2.63
IGHM	Immunoglobulin Heavy Constant Mu	38	5.07
IGHMBP2	Immunoglobulin Mu Binding Protein 2	57	8.74
IGKC	Immunoglobulin Kappa Constant	45	0.57
IGLL1	Immunoglobulin Lambda Like Polypeptide 1	59	3.8
IGSF6	Immunoglobulin Superfamily Member 6	50	6
IGSF6-DREV1	Region Containing Immunoglobulin Superfamily, Member 6 And DREV1	4	4.12
IKBKB	Inhibitor Of Nuclear Factor Kappa B Kinase Subunit Beta	75	5.71
IKBKG	Inhibitor Of Nuclear Factor Kappa B Kinase Subunit Gamma	66	13.39
IKZF1	IKAROS Family Zinc Finger 1	63	8.82
IKZF3	IKAROS Family Zinc Finger 3	58	1.49
IL10	Interleukin 10	66	68.57
IL10RA	Interleukin 10 Receptor Subunit Alpha	62	26.84
IL10RB	Interleukin 10 Receptor Subunit Beta	63	20.85
IL11	Interleukin 11	58	10.78
IL12A	Interleukin 12A	64	18.1
IL12A-AS1	IL12A Antisense RNA 1	20	15.35
IL12B	Interleukin 12B	66	22.96
IL12RB1	Interleukin 12 Receptor Subunit Beta 1	62	9.41
IL12RB2	Interleukin 12 Receptor Subunit Beta 2	62	7.77
IL13	Interleukin 13	68	30.77

IL15	Interleukin 15	62	11.16
IL16	Interleukin 16	61	6.19
IL17A	Interleukin 17A	67	23.71
IL17B	Interleukin 17B	58	0.77
IL17C	Interleukin 17C	54	2.26
IL17F	Interleukin 17F	63	5.31
IL17RA	Interleukin 17 Receptor A	63	10.2
IL17RC	Interleukin 17 Receptor C	58	5.44
IL17RE	Interleukin 17 Receptor E	56	0.66
IL18	Interleukin 18	62	21.76
IL18BP	Interleukin 18 Binding Protein	56	5.86
IL18R1	Interleukin 18 Receptor 1	62	3.91
IL18RAP	Interleukin 18 Receptor Accessory Protein	58	7.73
IL1A	Interleukin 1 Alpha	65	24.32
IL1B	Interleukin 1 Beta	70	45.02
IL1R1	Interleukin 1 Receptor Type 1	68	18.51
IL1R2	Interleukin 1 Receptor Type 2	63	3.78
IL1RAPL2	Interleukin 1 Receptor Accessory Protein Like 2	55	11.55
IL1RL1	Interleukin 1 Receptor Like 1	60	1.54
IL1RN	Interleukin 1 Receptor Antagonist	66	27.95
IL2	Interleukin 2	71	21.47
IL21	Interleukin 21	62	16.35
IL21R	Interleukin 21 Receptor	64	2.1
IL23A	Interleukin 23 Subunit Alpha	61	8.87
IL23R	Interleukin 23 Receptor	62	33.5
IL24	Interleukin 24	60	1.5
IL27	Interleukin 27	54	4.05
IL2RA	Interleukin 2 Receptor Subunit Alpha	73	20.92
IL2RB	Interleukin 2 Receptor Subunit Beta	66	13.73
IL3	Interleukin 3	64	9.6
IL31RA	Interleukin 31 Receptor A	61	3.39
IL32	Interleukin 32	55	1.32
IL33	Interleukin 33	58	3.17
IL36RN	Interleukin 36 Receptor Antagonist	55	11.82
IL37	Interleukin 37	51	6.34
IL3RA	Interleukin 3 Receptor Subunit Alpha	62	0.85
IL4	Interleukin 4	69	22.07
IL4R	Interleukin 4 Receptor	66	6.46
IL5	Interleukin 5	68	17.53
IL6	Interleukin 6	70	76.33
IL6R	Interleukin 6 Receptor	68	7.03
IL6ST	Interleukin 6 Signal Transducer	65	6.6
IL7	Interleukin 7	62	4.94
IL7R	Interleukin 7 Receptor	65	6.12
IL9	Interleukin 9	65	4.1
IMP3	IMP3, U3 Small Nucleolar Ribonucleoprotein	51	0.57
INHA	Inhibin Alpha Subunit	61	3.13
INHBA	Inhibin Beta A Subunit	65	1.14
INPP5E	Inositol Polyphosphate-5-Phosphatase E	58	1.75
INS	Insulin	67	12.96
INSR	Insulin Receptor	78	1.55
INVS	Inversin	60	2.05
IRAK1	Interleukin 1 Receptor Associated Kinase 1	70	1.48
IRAK2	Interleukin 1 Receptor Associated Kinase 2	62	1.01
IRAK3	Interleukin 1 Receptor Associated Kinase 3	67	3.49
IRAK4	Interleukin 1 Receptor Associated Kinase 4	68	4.09
IRF1	Interferon Regulatory Factor 1	63	2.68
IRF3	Interferon Regulatory Factor 3	66	2.01
IRF4	Interferon Regulatory Factor 4	62	4.58

IRF5	Interferon Regulatory Factor 5	65	20.69
IRF7	Interferon Regulatory Factor 7	65	1.6
IRF8	Interferon Regulatory Factor 8	62	1.03
IRGM	Immunity Related GTPase M	54	15.23
IRS1	Insulin Receptor Substrate 1	66	4.66
IRS2	Insulin Receptor Substrate 2	60	0.72
ISG15	ISG15 Ubiquitin-Like Modifier	69	0.66
ISG20	Interferon Stimulated Exonuclease Gene 20	57	0.85
ISL1	ISL LIM Homeobox 1	62	0.66
ITCH	Itchy E3 Ubiquitin Protein Ligase	63	8.31
ITGA1	Integrin Subunit Alpha 1	59	1.12
ITGA2	Integrin Subunit Alpha 2	66	6.58
ITGA2B	Integrin Subunit Alpha 2b	70	1.69
ITGA3	Integrin Subunit Alpha 3	65	0.93
ITGA4	Integrin Subunit Alpha 4	66	8.9
ITGA6	Integrin Subunit Alpha 6	69	3.5
ITGAE	Integrin Subunit Alpha E	58	1.11
ITGAL	Integrin Subunit Alpha L	65	7.26
ITGAM	Integrin Subunit Alpha M	66	17.09
ITGAV	Integrin Subunit Alpha V	69	1.63
ITGAX	Integrin Subunit Alpha X	65	3.22
ITGB1	Integrin Subunit Beta 1	71	4.79
ITGB2	Integrin Subunit Beta 2	72	9.97
ITGB3	Integrin Subunit Beta 3	70	2.95
ITGB4	Integrin Subunit Beta 4	67	2.49
ITGB6	Integrin Subunit Beta 6	66	0.54
ITGB8	Integrin Subunit Beta 8	58	0.57
ITIH4	Inter-Alpha-Trypsin Inhibitor Heavy Chain Family Member 4	59	2.27
ITPR1	Inositol 1,4,5-Trisphosphate Receptor Type 1	68	1.52
ITPR3	Inositol 1,4,5-Trisphosphate Receptor Type 3	66	0.99
JAK1	Janus Kinase 1	73	1.86
JAK2	Janus Kinase 2	76	28.02
JAK3	Janus Kinase 3	73	2.62
JAZF1	JAZF Zinc Finger 1	55	0.95
JMJD1C	Jumonji Domain Containing 1C	58	5.43
JMJD6	Arginine Demethylase And Lysine Hydroxylase	59	0.72
JUN	Jun Proto-Oncogene, AP-1 Transcription Factor Subunit	73	14.06
JUND	JunD Proto-Oncogene, AP-1 Transcription Factor Subunit	58	1.03
KANTR	KDM5C Adjacent Transcript	19	6.84
KARS	Lysyl-TRNA Synthetase	66	5.81
KBTBD13	Kelch Repeat And BTB Domain Containing 13	45	2.96
KCNA2	Potassium Voltage-Gated Channel Subfamily A Member 2	63	2.58
KCNE5	Potassium Voltage-Gated Channel Subfamily E Regulatory Subunit 5	44	0.93
KCNH2	Potassium Voltage-Gated Channel Subfamily H Member 2	70	1.49
KCNJ11	Potassium Voltage-Gated Channel Subfamily J Member 11	68	3.13
KCNJ6	Potassium Voltage-Gated Channel Subfamily J Member 6	62	1.66
KCNQ1	Potassium Voltage-Gated Channel Subfamily Q Member 1	67	2.66
KCTD1	Potassium Channel Tetramerization Domain Containing 1	54	2.18
KDR	Kinase Insert Domain Receptor	79	7.45
KEAP1	Kelch Like ECH Associated Protein 1	65	0.88
KIAA1109	KIAA1109	50	0.95
KIAA1468	KIAA1468	50	3.14
KIF5A	Kinesin Family Member 5A	63	2.82
KIR2DL1	Killer Cell Immunoglobulin Like Receptor, Two Ig Domains And Long Cytoplasmic Tail 1	53	4.57
KIR3DL1	Killer Cell Immunoglobulin Like Receptor, Three Ig Domains And Long Cytoplasmic Tail 1	60	4.75
KIR3DS1	Killer Cell Immunoglobulin Like Receptor, Three Ig Domains And Short Cytoplasmic Tail 1	36	2.6
KIT	KIT Proto-Oncogene Receptor Tyrosine Kinase	75	29.68
KITLG	KIT Ligand	63	7.54
KL	Klotho	62	3.77

KLF12	Kruppel Like Factor 12	54	3.51
KLF4	Kruppel Like Factor 4	65	1.48
KLF5	Kruppel Like Factor 5	62	0.73
KLF6	Kruppel Like Factor 6	61	2.17
KLHDC8B	Kelch Domain Containing 8B	51	5.82
KLHL40	Kelch Like Family Member 40	50	2.6
KLHL41	Kelch Like Family Member 41	50	2.6
KLK3	Kallikrein Related Peptidase 3	68	8.99
KLK4	Kallikrein Related Peptidase 4	60	3.66
KLK7	Kallikrein Related Peptidase 7	59	3.25
KLKB1	Kallikrein B1	69	1.52
KLRC3	Killer Cell Lectin Like Receptor C3	53	3.93
KLRC4	Killer Cell Lectin Like Receptor C4	50	15.35
KLRK1	Killer Cell Lectin Like Receptor K1	58	1.15
KMO	Kynurenine 3-Monooxygenase	61	0.95
KMT2D	Lysine Methyltransferase 2D	57	1.47
KNG1	Kininogen 1	63	15.77
KPNA2	Karyopherin Subunit Alpha 2	66	0.66
KRAS	KRAS Proto-Oncogene, GTPase	70	7.71
KRT1	Keratin 1	62	5.3
KRT10	Keratin 10	59	8.2
KRT13	Keratin 13	58	3
KRT14	Keratin 14	66	14.09
KRT16	Keratin 16	59	12.46
KRT17	Keratin 17	65	9.18
KRT18	Keratin 18	69	4.76
KRT19	Keratin 19	66	6.21
KRT5	Keratin 5	66	4.48
KRT6B	Keratin 6B	56	2.38
KRT7	Keratin 7	60	4.37
KRT75	Keratin 75	50	0.86
KRT8	Keratin 8	67	11.34
KRT9	Keratin 9	51	4.05
KYNU	Kynureninase	62	1.69
LACC1	Laccase Domain Containing 1	45	22.14
LACTB	Lactamase Beta	53	4.17
LAIR1	Leukocyte Associated Immunoglobulin Like Receptor 1	59	2.26
LALBA	Lactalbumin Alpha	56	4.59
LAMA2	Laminin Subunit Alpha 2	60	3.96
LAMA3	Laminin Subunit Alpha 3	62	2.15
LAMP1	Lysosomal Associated Membrane Protein 1	63	0.73
LAMP2	Lysosomal Associated Membrane Protein 2	66	2.43
LAP3	Leucine Aminopeptidase 3	60	2.97
LAT2	Linker For Activation Of T-Cells Family Member 2	56	0.73
LBP	Lipopolysaccharide Binding Protein	66	6.64
LBR	Lamin B Receptor	64	6.57
LCAT	Lecithin-Cholesterol Acyltransferase	65	5.94
LCK	LCK Proto-Oncogene, Src Family Tyrosine Kinase	75	2.86
LCN2	Lipocalin 2	65	1.94
LCT	Lactase	62	0.88
LDB3	LIM Domain Binding 3	57	9.9
LDHB	Lactate Dehydrogenase B	68	1.5
LDLR	Low Density Lipoprotein Receptor	72	8.22
LEP	Leptin	67	14.23
LEPQTL1	Leptin, Serum Levels Of	8	1.72
LEPR	Leptin Receptor	70	1.61
LGALS1	Galectin 1	62	1.07
LGALS2	Galectin 2	57	1.34
LGALS3	Galectin 3	66	3.85

LGALS4	Galectin 4	55	0.81
LHCGR	Luteinizing Hormone/Choriogonadotropin Receptor	67	2.31
LIAS	Lipoic Acid Synthetase	61	0.66
LIF	LIF, Interleukin 6 Family Cytokine	64	1.91
LIFR	LIF Receptor Alpha	67	5.09
LIG4	DNA Ligase 4	66	3.23
LILRB3	Leukocyte Immunoglobulin Like Receptor B3	58	3.12
LIMK1	LIM Domain Kinase 1	69	7.9
LIPA	Lipase A, Lysosomal Acid Type	64	1.34
LIPC	Lipase C, Hepatic Type	63	4.03
LIPE	Lipase E, Hormone Sensitive Type	66	0.66
LIPN	Lipase Family Member N	49	3.3
LITAF	Lipopolysaccharide Induced TNF Factor	58	2.42
LMBRD1	LMBR1 Domain Containing 1	56	2.04
LMF1	Lipase Maturation Factor 1	51	6.57
LMNA	Lamin A/C	66	10.45
LMNB2	Lamin B2	59	2.96
LMOD1	Leiomodin 1	53	0.57
LMOD3	Leiomodin 3	54	2.6
LMX1B	LIM Homeobox Transcription Factor 1 Beta	61	1.83
LNPEP	Leucyl And Cystinyl Aminopeptidase	65	3.19
LOR	Loricrin	51	2.46
LOX	Lysyl Oxidase	62	6.58
LOXL3	Lysyl Oxidase Like 3	59	1.31
LPA	Lipoprotein(A)	58	6.66
LPAR1	Lysophosphatidic Acid Receptor 1	66	2.42
LPIN1	Lipin 1	64	8.36
LPIN2	Lipin 2	58	18.7
LPL	Lipoprotein Lipase	70	10.58
LPO	Lactoperoxidase	56	2.43
LRBA	LPS Responsive Beige-Like Anchor Protein	54	6.81
LRG1	Leucine Rich Alpha-2-Glycoprotein 1	56	0.95
LRP1	LDL Receptor Related Protein 1	66	8.76
LRP5	LDL Receptor Related Protein 5	67	6.69
LRRC8A	Leucine Rich Repeat Containing 8 Family Member A	55	3.8
LRRFIP2	LRR Binding FLII Interacting Protein 2	52	0.57
LRRK2	Leucine Rich Repeat Kinase 2	69	4.02
LTA	Lymphotoxin Alpha	63	25.94
LTBP1	Latent Transforming Growth Factor Beta Binding Protein 1	63	0.66
LTC4S	Leukotriene C4 Synthase	59	5.54
LTF	Lactotransferrin	63	10.45
LUZP4	Leucine Zipper Protein 4	47	2.05
LXN	Latexin	52	1.92
LY75	Lymphocyte Antigen 75	54	3.28
LY86	Lymphocyte Antigen 86	52	1.42
LY9	Lymphocyte Antigen 9	54	2.05
LY96	Lymphocyte Antigen 96	62	2.21
LYN	LYN Proto-Oncogene, Src Family Tyrosine Kinase	72	2.69
LYRM4	LYR Motif Containing 4	54	0.95
LYST	Lysosomal Trafficking Regulator	54	3.89
LYVE1	Lymphatic Vessel Endothelial Hyaluronan Receptor 1	59	3.42
LYZ	Lysozyme	66	5.96
LZTS1	Leucine Zipper Tumor Suppressor 1	51	6.55
MADCAM1	Mucosal Vascular Addressin Cell Adhesion Molecule 1	54	5.49
MAF	MAF BZIP Transcription Factor	59	2.27
MAG	Myelin Associated Glycoprotein	65	6.31
MAGEC1	MAGE Family Member C1	46	2.8
MALAT1	Metastasis Associated Lung Adenocarcinoma Transcript 1 (Non-Protein Coding)	26	0.93
MALT1	MALT1 Paracaspase	66	6.99

MAML2	Mastermind Like Transcriptional Coactivator 2	52	1.03
MAN1B1	Mannosidase Alpha Class 1B Member 1	65	1.38
MANBA	Mannosidase Beta	59	0.73
MAOA	Monoamine Oxidase A	66	3.65
MAOB	Monoamine Oxidase B	61	2.86
MAP2	Microtubule Associated Protein 2	62	4.63
MAP2K1	Mitogen-Activated Protein Kinase Kinase 1	79	2.66
MAP2K2	Mitogen-Activated Protein Kinase Kinase 2	76	1.77
MAP2K3	Mitogen-Activated Protein Kinase Kinase 3	71	2.9
MAP2K4	Mitogen-Activated Protein Kinase Kinase 4	66	2.78
MAP2K5	Mitogen-Activated Protein Kinase Kinase 5	66	1.38
MAP2K6	Mitogen-Activated Protein Kinase Kinase 6	67	2.85
MAP3K8	Mitogen-Activated Protein Kinase Kinase Kinase 8	66	1.4
MAPK1	Mitogen-Activated Protein Kinase 1	75	8.17
MAPK10	Mitogen-Activated Protein Kinase 10	72	4.92
MAPK11	Mitogen-Activated Protein Kinase 11	70	2.72
MAPK12	Mitogen-Activated Protein Kinase 12	67	2.35
MAPK13	Mitogen-Activated Protein Kinase 13	67	2.24
MAPK14	Mitogen-Activated Protein Kinase 14	75	10.41
MAPK3	Mitogen-Activated Protein Kinase 3	73	8.82
MAPK7	Mitogen-Activated Protein Kinase 7	67	3
MAPK8	Mitogen-Activated Protein Kinase 8	74	7.5
MAPK8IP1	Mitogen-Activated Protein Kinase 8 Interacting Protein 1	62	1.03
MAPK9	Mitogen-Activated Protein Kinase 9	70	2.36
MAPKAPK3	Mitogen-Activated Protein Kinase-Activated Protein Kinase 3	70	1.03
MAPT	Microtubule Associated Protein Tau	74	18.37
MASP2	Mannan Binding Lectin Serine Peptidase 2	61	11.66
MATN1	Matrilin 1, Cartilage Matrix Protein	55	3
MATR3	Matrin 3	58	7.55
MAVS	Mitochondrial Antiviral Signaling Protein	58	1.2
MB	Myoglobin	60	11.97
MB21D1	Mab-21 Domain Containing 1	50	0.73
MBL2	Mannose Binding Lectin 2	66	10.86
MBP	Myelin Basic Protein	65	10.65
MBTPS2	Membrane Bound Transcription Factor Peptidase, Site 2	58	11.36
MC1R	Melanocortin 1 Receptor	66	5.27
MC2R	Melanocortin 2 Receptor	66	3.93
MCAM	Melanoma Cell Adhesion Molecule	58	1.05
MCL1	MCL1, BCL2 Family Apoptosis Regulator	69	5.98
MCM2	Minichromosome Maintenance Complex Component 2	66	0.73
MCM3	Minichromosome Maintenance Complex Component 3	65	0.97
MCRS1	Microspherule Protein 1	54	0.73
MDM2	MDM2 Proto-Oncogene	73	11.53
MECP2	Methyl-CpG Binding Protein 2	63	10.18
MEF2C	Myocyte Enhancer Factor 2C	66	0.99
MEFV	MEFV, Pyrin Innate Immunity Regulator	59	40.64
MEGF10	Multiple EGF Like Domains 10	56	3.51
MEN1	Menin 1	64	17.11
MERTK	MER Proto-Oncogene, Tyrosine Kinase	70	0.54
MET	MET Proto-Oncogene, Receptor Tyrosine Kinase	79	17.94
METTL9	Methyltransferase Like 9	48	4.12
MFGE8	Milk Fat Globule-EGF Factor 8 Protein	62	0.78
MGAM	Maltase-Glucoamylase	59	1.56
MGLL	Monoglyceride Lipase	64	4.56
MGME1	Mitochondrial Genome Maintenance Exonuclease 1	48	3.67
MGMT	O-6-Methylguanine-DNA Methyltransferase	69	2
MGP	Matrix Gla Protein	58	4.81
MIA	Melanoma Inhibitory Activity	55	2.05
MICA	MHC Class I Polypeptide-Related Sequence A	54	9.76

MICB	MHC Class I Polypeptide-Related Sequence B	58	5.56
MIF	Macrophage Migration Inhibitory Factor (Glycosylation-Inhibiting Factor)	68	35.15
MIR132	MicroRNA 132	23	1.27
MIR195	MicroRNA 195	19	0.57
MIR34A	MicroRNA 34a	26	1.14
MITF	Melanogenesis Associated Transcription Factor	66	8.64
MKI67	Marker Of Proliferation Ki-67	60	8.13
MKKS	McKusick-Kaufman Syndrome	54	0.99
MKL1	Megakaryoblastic Leukemia (Translocation) 1	56	0.73
MLANA	Melan-A	54	2.56
MLH1	MutL Homolog 1	66	17.48
MLH3	MutL Homolog 3	55	3.04
MLX	MLX, MAX Dimerization Protein	59	11.19
MLXIPL	MLX Interacting Protein Like	57	3.52
MME	Membrane Metalloendopeptidase	73	5.45
MMEL1	Membrane Metalloendopeptidase Like 1	56	2
MMP1	Matrix Metallopeptidase 1	74	17.67
MMP10	Matrix Metallopeptidase 10	68	3.97
MMP11	Matrix Metallopeptidase 11	64	3.17
MMP12	Matrix Metallopeptidase 12	64	1.35
MMP13	Matrix Metallopeptidase 13	72	8.37
MMP14	Matrix Metallopeptidase 14	73	2.22
MMP2	Matrix Metallopeptidase 2	76	17.18
MMP24	Matrix Metallopeptidase 24	61	1.25
MMP3	Matrix Metallopeptidase 3	75	18.64
MMP7	Matrix Metallopeptidase 7	70	5.75
MMP8	Matrix Metallopeptidase 8	70	9.2
MMP9	Matrix Metallopeptidase 9	76	21.77
MNX1	Motor Neuron And Pancreas Homeobox 1	56	5.28
MOBP	Myelin-Associated Oligodendrocyte Basic Protein	50	2.6
MOG	Myelin Oligodendrocyte Glycoprotein	63	5.79
MORC3	MORC Family CW-Type Zinc Finger 3	51	3.34
MPO	Myeloperoxidase	71	33.45
MPV17	MPV17, Mitochondrial Inner Membrane Protein	57	6.51
MPZ	Myelin Protein Zero	61	22.41
MRAP	Melanocortin 2 Receptor Accessory Protein	51	10.33
MRC1	Mannose Receptor C-Type 1	54	1.15
MRI1	Methylthioribose-1-Phosphate Isomerase 1	52	3.13
MS4A1	Membrane Spanning 4-Domains A1	64	8.81
MS4A2	Membrane Spanning 4-Domains A2	61	5.49
MSH2	MutS Homolog 2	67	12.43
MSMB	Microseminoprotein Beta	54	6.55
MSN	Moesin	65	1.26
MSR1	Macrophage Scavenger Receptor 1	63	2.7
MST1	Macrophage Stimulating 1	63	6.87
MST1R	Macrophage Stimulating 1 Receptor	67	1.15
MSTN	Myostatin	64	4.78
MSX1	Msh Homeobox 1	62	2.36
MSX2	Msh Homeobox 2	64	1.45
MTA1	Metastasis Associated 1	60	2.2
MT-ATP8	Mitochondrially Encoded ATP Synthase 8	37	4.11
MT-CO1	Mitochondrially Encoded Cytochrome C Oxidase I	44	18.51
MT-CO2	Mitochondrially Encoded Cytochrome C Oxidase II	43	7.24
MT-CO3	Mitochondrially Encoded Cytochrome C Oxidase III	40	7.67
MT-CYB	Mitochondrially Encoded Cytochrome B	42	2.85
MTHFD1	Methylenetetrahydrofolate Dehydrogenase Synthetase 1	62	1.03
MTHFR	Methylenetetrahydrofolate Reductase	64	19.17
MTM1	Myotubularin 1	62	5.08
MTMR14	Myotubularin Related Protein 14	58	5.81

MTMR3	Myotubularin Related Protein 3	60	0.66
MT-ND1	Mitochondrially Encoded NADH:Ubiquinone Oxidoreductase Core Subunit 1	43	16.26
MT-ND4	Mitochondrially Encoded NADH:Ubiquinone Oxidoreductase Core Subunit 4	43	14.51
MT-ND5	Mitochondrially Encoded NADH:Ubiquinone Oxidoreductase Core Subunit 5	42	7.68
MT-ND6	Mitochondrially Encoded NADH:Ubiquinone Oxidoreductase Core Subunit 6	41	8.98
MTOR	Mechanistic Target Of Rapamycin	75	8.13
MTR	5-Methyltetrahydrofolate-Homocysteine Methyltransferase	65	1.51
MTRR	5-Methyltetrahydrofolate-Homocysteine Methyltransferase Reductase	59	1.29
MT-TE	Mitochondrially Encoded TRNA Glutamic Acid	20	2.55
MT-TF	Mitochondrially Encoded TRNA Phenylalanine	20	7.06
MT-TH	Mitochondrially Encoded TRNA Histidine	19	7.06
MT-TK	Mitochondrially Encoded TRNA Lysine	20	6.95
MT-TL1	Mitochondrially Encoded TRNA Leucine 1 (UUA/G)	21	7.54
MTTP	Microsomal Triglyceride Transfer Protein	58	1.49
MT-TQ	Mitochondrially Encoded TRNA Glutamine	16	7.4
MT-TS1	Mitochondrially Encoded TRNA Serine 1 (UCN)	20	7.06
MT-TS2	Mitochondrially Encoded TRNA Serine 2 (AGU/C)	20	7.06
MT-TV	Mitochondrially Encoded TRNA Valine	21	0.91
MT-TW	Mitochondrially Encoded TRNA Tryptophan	20	11.17
MUC1	Mucin 1, Cell Surface Associated	66	4.7
MUC12	Mucin 12, Cell Surface Associated	43	0.73
MUC13	Mucin 13, Cell Surface Associated	50	0.73
MUC16	Mucin 16, Cell Surface Associated	54	5.02
MUC17	Mucin 17, Cell Surface Associated	52	0.73
MUC19	Mucin 19, Oligomeric	41	2.4
MUC2	Mucin 2, Oligomeric Mucus/Gel-Forming	58	9.37
MUC3A	Mucin 3A, Cell Surface Associated	45	3.74
MUC3B	Mucin 3B, Cell Surface Associated	26	0.73
MUC4	Mucin 4, Cell Surface Associated	57	6
MUC5AC	Mucin 5AC, Oligomeric Mucus/Gel-Forming	52	7.64
MUC5B	Mucin 5B, Oligomeric Mucus/Gel-Forming	56	9.44
MUC6	Mucin 6, Oligomeric Mucus/Gel-Forming	54	4.38
MUC7	Mucin 7, Secreted	51	6.28
MUT	Methylmalonyl-CoA Mutase	62	5.47
MUTYH	MutY DNA Glycosylase	59	2.55
MVK	Mevalonate Kinase	67	23.81
MX1	MX Dynamin Like GTPase 1	58	5.85
MYB	MYB Proto-Oncogene, Transcription Factor	70	3.24
MYC	MYC Proto-Oncogene, BHLH Transcription Factor	75	10.09
MYD88	Myeloid Differentiation Primary Response 88	68	7.45
MYDGF	Myeloid Derived Growth Factor	45	0.88
MYF6	Myogenic Factor 6	57	6.28
MYH11	Myosin Heavy Chain 11	62	4.95
MYH2	Myosin Heavy Chain 2	63	9.29
MYH6	Myosin Heavy Chain 6	62	9.38
MYH7	Myosin Heavy Chain 7	66	10.78
MYH9	Myosin Heavy Chain 9	67	3.55
MYL4	Myosin Light Chain 4	63	2.31
MYLK	Myosin Light Chain Kinase	71	7.45
MYO9B	Myosin IXB	59	7.34
MYOC	Myocilin	61	0.57
MYOD1	Myogenic Differentiation 1	63	1.33
MYOG	Myogenin	60	5.89
MYOM2	Myomesin 2	55	5.86
MYOT	Myotilin	56	4.09
MYOZ2	Myozenin 2	53	2.3
NAGLU	N-Acetyl-Alpha-Glucosaminidase	59	4.95
NAIP	NLR Family Apoptosis Inhibitory Protein	58	1.88
NAMPT	Nicotinamide Phosphoribosyltransferase	67	2.41

NANOG	Nanog Homeobox	62	0.95
NARS	Asparaginyl-TRNA Synthetase	62	2.05
NAT1	N-Acetyltransferase 1	63	2.39
NAT2	N-Acetyltransferase 2	61	4.67
NBN	Nibrin	65	4.27
NBR1	NBR1, Autophagy Cargo Receptor	56	3.67
NCAM1	Neural Cell Adhesion Molecule 1	66	6.73
NCF1	Neutrophil Cytosolic Factor 1	66	6.03
NCK1	NCK Adaptor Protein 1	63	2.05
NCKIPSD	NCK Interacting Protein With SH3 Domain	50	2.05
NCOR1	Nuclear Receptor Corepressor 1	63	1.2
NCOR2	Nuclear Receptor Corepressor 2	62	1
NCR3	Natural Cytotoxicity Triggering Receptor 3	55	1.13
NCSTN	Nicastrin	66	13.15
NDRG1	N-Myc Downstream Regulated 1	62	3.14
NDUFA13	NADH:Ubiquinone Oxidoreductase Subunit A13	60	3.76
NDUFS3	NADH:Ubiquinone Oxidoreductase Core Subunit S3	64	2.6
NEB	Nebulin	57	2.85
NEDD4	Neural Precursor Cell Developmentally Down-Regulated 4, E3 Ubiquitin Protein Ligase	65	1.6
NEDD4L	Neural Precursor Cell Developmentally Down-Regulated 4-Like, E3 Ubiquitin Protein Ligase	64	0.57
NEK7	NIMA Related Kinase 7	60	0.73
NEK8	NIMA Related Kinase 8	57	5.82
NES	Nestin	58	1.59
NEU1	Neuraminidase 1	61	1.44
NEXN	Nexilin F-Actin Binding Protein	53	8.53
NF1	Neurofibromin 1	67	12.42
NFASC	Neurofascin	61	1.25
NFE2L2	Nuclear Factor, Erythroid 2 Like 2	66	4
NFIA	Nuclear Factor I A	59	0.57
NFKB1	Nuclear Factor Kappa B Subunit 1	75	16.82
NFKB2	Nuclear Factor Kappa B Subunit 2	73	7.69
NFKBIA	NFKB Inhibitor Alpha	72	15.24
NFKBIB	NFKB Inhibitor Beta	58	0.71
NFKBIE	NFKB Inhibitor Epsilon	55	1.03
NFKBIL1	NFKB Inhibitor Like 1	51	9.77
NFKBIZ	NFKB Inhibitor Zeta	54	2.5
NGF	Nerve Growth Factor	70	31.99
NGFR	Nerve Growth Factor Receptor	66	7.88
NHP2	NHP2 Ribonucleoprotein	58	1.68
NID1	Nidogen 1	60	2.6
NINJ1	Ninjurin 1	55	1.46
NIPAL4	NIPA Like Domain Containing 4	54	2.09
NIPSNAP1	Nipsnap Homolog 1	57	2.24
NKX2-3	NK2 Homeobox 3	52	4.25
NKX3-1	NK3 Homeobox 1	58	0.85
NLRC4	NLR Family CARD Domain Containing 4	61	8.94
NLRP1	NLR Family Pyrin Domain Containing 1	62	4.64
NLRP12	NLR Family Pyrin Domain Containing 12	62	13.69
NLRP3	NLR Family Pyrin Domain Containing 3	65	35.25
NME1	NME/NM23 Nucleoside Diphosphate Kinase 1	65	13.6
NMU	Neuromedin U	55	2.85
NMUR1	Neuromedin U Receptor 1	58	0.97
NOD1	Nucleotide Binding Oligomerization Domain Containing 1	62	10.57
NOD2	Nucleotide Binding Oligomerization Domain Containing 2	62	52.98
NOP10	NOP10 Ribonucleoprotein	54	1.68
NOS1	Nitric Oxide Synthase 1	67	5.17
NOS2	Nitric Oxide Synthase 2	68	23.76
NOS3	Nitric Oxide Synthase 3	72	18.38
NOTCH1	Notch 1	73	2.46

NOTCH2	Notch 2	70	6.12
NOTCH3	Notch 3	69	9.51
NOV	Nephroblastoma Overexpressed	60	6.16
NOX1	NADPH Oxidase 1	58	3.69
NOX4	NADPH Oxidase 4	65	1.28
NPBWR1	Neuropeptides B And W Receptor 1	55	0.97
NPFF	Neuropeptide FF-Amide Peptide Precursor	50	2.81
NPHP1	Nephrocystin 1	58	3.53
NPHP3	Nephrocystin 3	54	6.29
NPHP4	Nephrocystin 4	52	2.05
NPHS1	NPHS1, Nephrin	66	1.11
NPM1	Nucleophosmin	66	12.94
NPPA	Natriuretic Peptide A	65	5.17
NPPB	Natriuretic Peptide B	63	9.5
NPR3	Natriuretic Peptide Receptor 3	62	0.57
NPSR1	Neuropeptide S Receptor 1	58	2.42
NPY	Neuropeptide Y	66	6.04
NPY2R	Neuropeptide Y Receptor Y2	63	0.59
NPY5R	Neuropeptide Y Receptor Y5	58	1.39
NQO1	NAD(P)H Quinone Dehydrogenase 1	70	3.63
NR0B2	Nuclear Receptor Subfamily 0 Group B Member 2	62	0.73
NR1H2	Nuclear Receptor Subfamily 1 Group H Member 2	68	3.52
NR1H3	Nuclear Receptor Subfamily 1 Group H Member 3	66	6.21
NR1H4	Nuclear Receptor Subfamily 1 Group H Member 4	68	2.47
NR1I2	Nuclear Receptor Subfamily 1 Group I Member 2	66	7.51
NR1I3	Nuclear Receptor Subfamily 1 Group I Member 3	64	1.04
NR3C1	Nuclear Receptor Subfamily 3 Group C Member 1	73	12.44
NR3C2	Nuclear Receptor Subfamily 3 Group C Member 2	67	3.74
NR4A2	Nuclear Receptor Subfamily 4 Group A Member 2	64	6.48
NR5A2	Nuclear Receptor Subfamily 5 Group A Member 2	62	1.24
NRAS	NRAS Proto-Oncogene, GTPase	70	10.9
NRG1	Neuregulin 1	65	4.48
NRTN	Neurturin	60	5.43
NSD1	Nuclear Receptor Binding SET Domain Protein 1	59	5.82
NSD2	Nuclear Receptor Binding SET Domain Protein 2	45	1.93
NSMF	NMDA Receptor Synaptonuclear Signaling And Neuronal Migration Factor	51	0.57
NSUN2	NOP2/Sun RNA Methyltransferase Family Member 2	58	4.3
NT5C1A	5'-Nucleotidase, Cytosolic IA	54	4.26
NT5E	5'-Nucleotidase Ecto	70	2.46
NTAN1	N-Terminal Asparagine Amidase	52	0.85
NTF3	Neurotrophin 3	65	2.37
NTF4	Neurotrophin 4	63	1.27
NTN1	Netrin 1	59	0.95
NTRK1	Neurotrophic Receptor Tyrosine Kinase 1	75	26.63
NTRK2	Neurotrophic Receptor Tyrosine Kinase 2	75	4.72
NTS	Neurotensin	57	4.17
NTSR1	Neurotensin Receptor 1	64	1.7
NUDT1	Nudix Hydrolase 1	59	0.57
NUP98	Nucleoporin 98	62	3.13
NUPR1	Nuclear Protein 1, Transcriptional Regulator	52	0.94
NXF1	Nuclear RNA Export Factor 1	58	0.57
OAT	Ornithine Aminotransferase	66	3.13
OBSCN	Obscurin, Cytoskeletal Calmodulin And Titin-Interacting RhoGEF	58	2.36
OCLN	Occludin	62	0.57
OCRL	OCRL, Inositol Polyphosphate-5-Phosphatase	61	2.67
ODC1	Ornithine Decarboxylase 1	67	4.46
OFD1	OFD1, Centriole And Centriolar Satellite Protein	56	3.08
OGG1	8-Oxoguanine DNA Glycosylase	64	7.65
OLAH	Oleoyl-ACP Hydrolase	50	3.93

OLR1	Oxidized Low Density Lipoprotein Receptor 1	62	4.19
OMP	Olfactory Marker Protein	53	3.82
OPA3	OPA3, Outer Mitochondrial Membrane Lipid Metabolism Regulator	54	2.31
OPLAH	5-Oxoprolinase (ATP-Hydrolysing)	54	6.86
OPRL1	Opioid Related Nociceptin Receptor 1	64	4.72
OPRM1	Opioid Receptor Mu 1	70	14.98
OPTN	Optineurin	60	3.28
ORAI1	ORAI Calcium Release-Activated Calcium Modulator 1	62	3.76
ORC6	Origin Recognition Complex Subunit 6	55	1.47
OSM	Oncostatin M	64	6.22
OSMR	Oncostatin M Receptor	64	0.94
OTC	Ornithine Carbamoyltransferase	64	1.64
OTULIN	OTU Deubiquitinase With Linear Linkage Specificity	47	2.38
OVOL2	Ovo Like Zinc Finger 2	51	1.83
OXA1L	OXA1L, Mitochondrial Inner Membrane Protein	54	2.46
OXT	Oxytocin/Neurophysin I Prepropeptide	61	7.8
OXTR	Oxytocin Receptor	66	1.71
P2RX1	Purinergic Receptor P2X 1	63	1.82
P2RX3	Purinergic Receptor P2X 3	58	6.27
P2RX4	Purinergic Receptor P2X 4	62	8.48
P2RX7	Purinergic Receptor P2X 7	66	10.26
P2RY1	Purinergic Receptor P2Y1	62	2.07
P2RY12	Purinergic Receptor P2Y12	67	3.04
P2RY2	Purinergic Receptor P2Y2	66	1.72
P2RY4	Pyrimidinergic Receptor P2Y4	59	0.97
P2RY6	Pyrimidinergic Receptor P2Y6	64	1.79
PABPN1	Poly(A) Binding Protein Nuclear 1	61	4.2
PADI2	Peptidyl Arginine Deiminase 2	59	0.85
PADI4	Peptidyl Arginine Deiminase 4	62	13.6
PAEP	Progestagen Associated Endometrial Protein	54	2.09
PAH	Phenylalanine Hydroxylase	66	2.88
PAK1	P21 (RAC1) Activated Kinase 1	67	1
PAPPA	Pappalysin 1	58	2.86
PARK7	Parkinsonism Associated Deglycase	64	2.92
PARP1	Poly(ADP-Ribose) Polymerase 1	72	3.15
PARP9	Poly(ADP-Ribose) Polymerase Family Member 9	55	1.44
PAX5	Paired Box 5	63	8.93
PAX6	Paired Box 6	65	2.05
PAX9	Paired Box 9	62	2.36
PBX1	PBX Homeobox 1	66	2.13
PC	Pyruvate Carboxylase	65	3.13
PCNA	Proliferating Cell Nuclear Antigen	70	4.95
PCSK9	Proprotein Convertase Subtilisin/Kexin Type 9	70	1.86
PCYT1A	Phosphate Cytidylyltransferase 1, Choline, Alpha	65	4.76
PDCD1	Programmed Cell Death 1	65	11.52
PDCD4	Programmed Cell Death 4	61	1.28
PDE10A	Phosphodiesterase 10A	64	0.95
PDE11A	Phosphodiesterase 11A	63	3.45
PDE3A	Phosphodiesterase 3A	66	0.68
PDE4A	Phosphodiesterase 4A	64	8.72
PDE4D	Phosphodiesterase 4D	70	3.09
PDE5A	Phosphodiesterase 5A	62	1.97
PDE6A	Phosphodiesterase 6A	63	0.57
PDE6B	Phosphodiesterase 6B	62	1.21
PDE6C	Phosphodiesterase 6C	61	0.57
PDE6G	Phosphodiesterase 6G	59	0.57
PDE6H	Phosphodiesterase 6H	60	0.57
PDE7A	Phosphodiesterase 7A	63	0.84
PDE8B	Phosphodiesterase 8B	64	0.57

PDGFA	Platelet Derived Growth Factor Subunit A	65	1.93
PDGFB	Platelet Derived Growth Factor Subunit B	70	4.45
PDGFRA	Platelet Derived Growth Factor Receptor Alpha	80	15.59
PDGFRB	Platelet Derived Growth Factor Receptor Beta	78	7.09
PDGFRL	Platelet Derived Growth Factor Receptor Like	58	2.58
PDIA3	Protein Disulfide Isomerase Family A Member 3	63	0.85
PDLIM4	PDZ And LIM Domain 4	53	1.25
PDPK1	3-Phosphoinositide Dependent Protein Kinase 1	69	0.55
PDPN	Podoplanin	60	3.74
PDYN	Prodynorphin	60	6.36
PEBP1	Phosphatidylethanolamine Binding Protein 1	62	1.34
PECAM1	Platelet And Endothelial Cell Adhesion Molecule 1	59	4.31
PEPD	Peptidase D	62	2.85
PER2	Period Circadian Clock 2	60	1.3
PER3	Period Circadian Clock 3	56	1.61
PF4	Platelet Factor 4	59	6.61
PFKM	Phosphofructokinase, Muscle	70	1.57
PGF	Placental Growth Factor	65	2.55
PGLYRP1	Peptidoglycan Recognition Protein 1	55	0.66
PGM1	Phosphoglucomutase 1	65	2.26
PGM3	Phosphoglucomutase 3	58	3.31
PGR	Progesterone Receptor	70	12.36
PGRMC1	Progesterone Receptor Membrane Component 1	62	3.48
PHACTR1	Phosphatase And Actin Regulator 1	49	3
PHB	Prohibitin	62	6.46
PHB2	Prohibitin 2	58	4.08
PHF10	PHD Finger Protein 10	50	2.05
PHKA2	Phosphorylase Kinase Regulatory Subunit Alpha 2	63	0.84
PHOX2A	Paired Like Homeobox 2a	59	2.05
PHOX2B	Paired Like Homeobox 2b	58	3.92
PHYH	Phytanoyl-CoA 2-Hydroxylase	62	2.31
PI3	Peptidase Inhibitor 3	58	3.29
PIEZO1	Piezo Type Mechanosensitive Ion Channel Component 1	49	1.47
PIGA	Phosphatidylinositol Glycan Anchor Biosynthesis Class A	60	7.85
PIGR	Polymeric Immunoglobulin Receptor	61	0.57
PIK3C2A	Phosphatidylinositol-4-Phosphate 3-Kinase Catalytic Subunit Type 2 Alpha	62	9
PIK3CA	Phosphatidylinositol-4,5-Bisphosphate 3-Kinase Catalytic Subunit Alpha	75	13.47
PIK3CB	Phosphatidylinositol-4,5-Bisphosphate 3-Kinase Catalytic Subunit Beta	69	1.22
PIK3CG	Phosphatidylinositol-4,5-Bisphosphate 3-Kinase Catalytic Subunit Gamma	69	5.71
PIK3R1	Phosphoinositide-3-Kinase Regulatory Subunit 1	70	7.29
PIK3R2	Phosphoinositide-3-Kinase Regulatory Subunit 2	66	3.03
PIK3R3	Phosphoinositide-3-Kinase Regulatory Subunit 3	61	2.32
PIM2	Pim-2 Proto-Oncogene, Serine/Threonine Kinase	64	0.95
PIN1	Peptidylprolyl Cis/Trans Isomerase, NIMA-Interacting 1	66	0.95
PINK1	PTEN Induced Putative Kinase 1	66	1.38
PITRM1	Pitrilysin Metallopeptidase 1	54	0.85
PITX1	Paired Like Homeodomain 1	60	3.45
PITX2	Paired Like Homeodomain 2	64	2.79
PKDREJ	Polycystin Family Receptor For Egg Jelly	49	2.36
PKLR	Pyruvate Kinase, Liver And RBC	66	1.47
PKM	Pyruvate Kinase, Muscle	66	0.88
PLA2G10	Phospholipase A2 Group X	63	2.05
PLA2G2A	Phospholipase A2 Group IIA	66	16.11
PLA2G4A	Phospholipase A2 Group IVA	70	6
PLA2G4C	Phospholipase A2 Group IVC	58	2
PLA2G6	Phospholipase A2 Group VI	67	5.14
PLA2G7	Phospholipase A2 Group VII	70	13.99
PLAT	Plasminogen Activator, Tissue Type	68	9.65
PLAU	Plasminogen Activator, Urokinase	75	13.35

PLAUR	Plasminogen Activator, Urokinase Receptor	66	3.21
PLCB1	Phospholipase C Beta 1	67	2.76
PLCB2	Phospholipase C Beta 2	66	2.32
PLCB3	Phospholipase C Beta 3	67	2.49
PLCB4	Phospholipase C Beta 4	65	4.17
PLCD1	Phospholipase C Delta 1	66	5.13
PLCE1	Phospholipase C Epsilon 1	58	1.75
PLCG1	Phospholipase C Gamma 1	67	3.08
PLCG2	Phospholipase C Gamma 2	68	14.99
PLEC	Plectin	57	2
PLEK	Pleckstrin	57	0.97
PLG	Plasminogen	68	9.85
PLOD1	Procollagen-Lysine,2-Oxoglutarate 5-Dioxygenase 1	60	3.45
PLP1	Proteolipid Protein 1	59	10.92
PLS3	Plastin 3	56	1.03
PLTP	Phospholipid Transfer Protein	60	1.2
PMEL	Premelanosome Protein	54	2.8
PML	Promyelocytic Leukemia	66	2.42
PMP2	Peripheral Myelin Protein 2	58	4.11
PMP22	Peripheral Myelin Protein 22	60	25.42
PMPCA	Peptidase, Mitochondrial Processing Alpha Subunit	58	1.75
PNKD	Paroxysmal Nonkinesigenic Dyskinesia	58	1.11
PNLIP	Pancreatic Lipase	66	4.38
PNOC	Prepronociceptin	57	5.19
PNPLA1	Patatin Like Phospholipase Domain Containing 1	50	2.01
PNPLA2	Patatin Like Phospholipase Domain Containing 2	62	4.21
PNPLA3	Patatin Like Phospholipase Domain Containing 3	60	1.81
PNPLA6	Patatin Like Phospholipase Domain Containing 6	61	4.56
POLB	DNA Polymerase Beta	66	3.63
POLG	DNA Polymerase Gamma, Catalytic Subunit	65	5.31
POLH	DNA Polymerase Eta	65	3.13
POMC	Proopiomelanocortin	68	16.21
POMGNT2	Protein O-Linked Mannose N-Acetylglucosaminyltransferase 2 (Beta 1,4-)	49	1.03
POMT1	Protein O-Mannosyltransferase 1	61	2.31
POMT2	Protein O-Mannosyltransferase 2	59	6.28
PON1	Paraoxonase 1	65	16.54
PON2	Paraoxonase 2	61	3.09
PON3	Paraoxonase 3	63	2.79
POR	Cytochrome P450 Oxidoreductase	68	2.09
POSTN	Periostin	64	1.18
POU2AF1	POU Class 2 Associating Factor 1	53	3.46
POU2F1	POU Class 2 Homeobox 1	63	0.81
POU5F1	POU Class 5 Homeobox 1	68	0.73
PPARA	Peroxisome Proliferator Activated Receptor Alpha	70	6.41
PPARD	Peroxisome Proliferator Activated Receptor Delta	69	4.18
PPARG	Peroxisome Proliferator Activated Receptor Gamma	76	23.37
PPARGC1A	PPARG Coactivator 1 Alpha	64	2.78
PPBP	Pro-Platelet Basic Protein	62	7.09
PPFIBP1	PPFIA Binding Protein 1	54	5.01
PPIA	Peptidylprolyl Isomerase A	70	3.08
PPIG	Peptidylprolyl Isomerase G	58	1
PPM1B	Protein Phosphatase, Mg2+/Mn2+ Dependent 1B	61	0.57
PPP1R14A	Protein Phosphatase 1 Regulatory Inhibitor Subunit 14A	55	0.57
PPP2R1A	Protein Phosphatase 2 Scaffold Subunit Aalpha	65	0.83
PPP2R1B	Protein Phosphatase 2 Scaffold Subunit Abeta	65	2.02
PPP3CA	Protein Phosphatase 3 Catalytic Subunit Alpha	73	0.93
PPRC1	Peroxisome Proliferator-Activated Receptor Gamma, Coactivator-Related 1	51	0.73
PRDM1	PR/SET Domain 1	65	3.54
PRDX3	Peroxiredoxin 3	62	0.57

PRDX5	Peroxiredoxin 5	65	1.65
PRF1	Perforin 1	64	13.05
PRG2	Proteoglycan 2, Pro Eosinophil Major Basic Protein	58	3.54
PRG4	Proteoglycan 4	56	11.76
PRICKLE2	Prickle Planar Cell Polarity Protein 2	55	0.46
PRKAA1	Protein Kinase AMP-Activated Catalytic Subunit Alpha 1	69	0.71
PRKAA2	Protein Kinase AMP-Activated Catalytic Subunit Alpha 2	72	1.12
PRKAB1	Protein Kinase AMP-Activated Non-Catalytic Subunit Beta 1	68	0.6
PRKACA	Protein Kinase CAMP-Activated Catalytic Subunit Alpha	71	10.39
PRKACB	Protein Kinase CAMP-Activated Catalytic Subunit Beta	67	2.42
PRKACG	Protein Kinase CAMP-Activated Catalytic Subunit Gamma	66	2.42
PRKAR1A	Protein Kinase CAMP-Dependent Type I Regulatory Subunit Alpha	71	13.25
PRKCA	Protein Kinase C Alpha	72	3.92
PRKCB	Protein Kinase C Beta	70	2.47
PRKCD	Protein Kinase C Delta	78	11.66
PRKCE	Protein Kinase C Epsilon	72	2.4
PRKCG	Protein Kinase C Gamma	73	6.36
PRKCH	Protein Kinase C Eta	71	3.81
PRKCI	Protein Kinase C Iota	71	1.52
PRKCQ	Protein Kinase C Theta	73	4.19
PRKCZ	Protein Kinase C Zeta	72	3.48
PRKD1	Protein Kinase D1	68	7.59
PRKDC	Protein Kinase, DNA-Activated, Catalytic Polypeptide	70	1.94
PRKG1	Protein Kinase, CGMP-Dependent, Type I	68	1.81
PRKG2	Protein Kinase, CGMP-Dependent, Type II	65	2.6
PRKN	Parkin RBR E3 Ubiquitin Protein Ligase	51	3.29
PRKRA	Protein Activator Of Interferon Induced Protein Kinase EIF2AK2	58	4.26
PRL	Prolactin	62	10.12
PRND	Prion Like Protein Doppel	53	2.6
PRNP	Prion Protein	66	14.43
PROC	Protein C, Inactivator Of Coagulation Factors Va And VIIIa	70	2.42
PROCR	Protein C Receptor	61	1.56
PROK1	Prokineticin 1	56	1.46
PROK2	Prokineticin 2	58	3.38
PROKR1	Prokineticin Receptor 1	58	0.85
PROS1	Protein S (Alpha)	66	2.49
PROX1	Prospero Homeobox 1	58	5.83
PROZ	Protein Z, Vitamin K Dependent Plasma Glycoprotein	58	0.95
PRPF8	Pre-MRNA Processing Factor 8	54	0.46
PRPS1	Phosphoribosyl Pyrophosphate Synthetase 1	62	11.43
PRRC2A	Proline Rich Coiled-Coil 2A	46	0.9
PRSS1	Protease, Serine 1	66	8.81
PRTN3	Proteinase 3	63	25.43
PSAT1	Phosphoserine Aminotransferase 1	66	0.85
PSC	Cholangitis, Primary Sclerosing	4	4.72
PSEN1	Presenilin 1	74	18.65
PSEN2	Presenilin 2	67	1.69
PSENEN	Presenilin Enhancer Gamma-Secretase Subunit	59	15.56
PSMA6	Proteasome Subunit Alpha 6	64	1.92
PSMB8	Proteasome Subunit Beta 8	70	10.08
PSMB9	Proteasome Subunit Beta 9	65	0.88
PSMD10	Proteasome 26S Subunit, Non-ATPase 10	59	0.73
PSMD9	Proteasome 26S Subunit, Non-ATPase 9	62	4.09
PSMG1	Proteasome Assembly Chaperone 1	54	0.57
PSORS1C1	Psoriasis Susceptibility 1 Candidate 1	41	3.87
PSTPIP1	Proline-Serine-Threonine Phosphatase Interacting Protein 1	61	27.88
PTAFR	Platelet Activating Factor Receptor	64	3.32
PTCH1	Patched 1	69	1.25
PTCRA	Pre T-Cell Antigen Receptor Alpha	54	1.11

PTEN	Phosphatase And Tensin Homolog	74	17.82
PTGDR	Prostaglandin D2 Receptor	66	1.72
PTGDS	Prostaglandin D2 Synthase	65	1.27
PTGER1	Prostaglandin E Receptor 1	62	2.47
PTGER2	Prostaglandin E Receptor 2	68	4.98
PTGER3	Prostaglandin E Receptor 3	66	2.58
PTGER4	Prostaglandin E Receptor 4	66	5.37
PTGES	Prostaglandin E Synthase	59	9.99
PTGES2	Prostaglandin E Synthase 2	61	1.1
PTGIR	Prostaglandin I2 (Prostacyclin) Receptor (IP)	66	1.16
PTGIS	Prostaglandin I2 Synthase	64	3.77
PTGS1	Prostaglandin-Endoperoxide Synthase 1	68	21.72
PTGS2	Prostaglandin-Endoperoxide Synthase 2	70	38.35
PTH	Parathyroid Hormone	64	6.27
PTHLH	Parathyroid Hormone Like Hormone	63	2.76
PTK2	Protein Tyrosine Kinase 2	70	1.95
PTK2B	Protein Tyrosine Kinase 2 Beta	69	3.09
PTN	Pleiotrophin	62	0.81
PTP4A3	Protein Tyrosine Phosphatase Type IVA, Member 3	60	2.05
PTPN1	Protein Tyrosine Phosphatase, Non-Receptor Type 1	71	0.88
PTPN11	Protein Tyrosine Phosphatase, Non-Receptor Type 11	71	2.44
PTPN13	Protein Tyrosine Phosphatase, Non-Receptor Type 13	63	2.6
PTPN2	Protein Tyrosine Phosphatase, Non-Receptor Type 2	66	7.07
PTPN22	Protein Tyrosine Phosphatase, Non-Receptor Type 22	63	32.7
PTPRC	Protein Tyrosine Phosphatase, Receptor Type C	70	9.94
PTPRJ	Protein Tyrosine Phosphatase, Receptor Type J	67	4.92
PTPRO	Protein Tyrosine Phosphatase, Receptor Type O	63	0.57
PTX3	Pentraxin 3	58	7.16
PUS1	Pseudouridylate Synthase 1	58	2.9
PVR	Poliovirus Receptor	62	1.02
PXK	PX Domain Containing Serine/Threonine Kinase Like	52	2.34
PYCARD	PYD And CARD Domain Containing	62	7.6
PYDC1	Pyrin Domain Containing 1	45	0.57
PYY	Peptide YY	61	3.95
RAB27A	RAB27A, Member RAS Oncogene Family	66	0.57
RAB7A	RAB7A, Member RAS Oncogene Family	66	3.94
RAC1	Ras-Related C3 Botulinum Toxin Substrate 1 (Rho Family, Small GTP Binding Protein Rac1)	69	3.43
RACK1	Receptor For Activated C Kinase 1	46	0.66
RAD21	RAD21 Cohesin Complex Component	62	1.83
RAF1	Raf-1 Proto-Oncogene, Serine/Threonine Kinase	79	4.23
RAG1	Recombination Activating 1	63	2.63
RAI1	Retinoic Acid Induced 1	54	9.39
RALB	RAS Like Proto-Oncogene B	65	0.85
RALBP1	RalA Binding Protein 1	65	0.88
RAN	RAN, Member RAS Oncogene Family	63	0.85
RANBP2	RAN Binding Protein 2	65	12.1
RAP1A	RAP1A, Member Of RAS Oncogene Family	65	1.22
RAPGEF3	Rap Guanine Nucleotide Exchange Factor 3	63	2.06
RARA	Retinoic Acid Receptor Alpha	71	2.02
RARB	Retinoic Acid Receptor Beta	68	2.26
RARS	Arginyl-TRNA Synthetase	63	1.5
RASA1	RAS P21 Protein Activator 1	65	9.89
RASGRP1	RAS Guanyl Releasing Protein 1	62	3.09
RASSF1	Ras Association Domain Family Member 1	62	4.21
RB1	RB Transcriptional Corepressor 1	69	4.75
RBCK1	RANBP2-Type And C3HC4-Type Zinc Finger Containing 1	58	4.79
RBM8A	RNA Binding Motif Protein 8A	55	2.57
RBP3	Retinol Binding Protein 3	54	5.23
RBP4	Retinol Binding Protein 4	63	2.63

RBPJ	Recombination Signal Binding Protein For Immunoglobulin Kappa J Region	65	2.62
RCAN1	Regulator Of Calcineurin 1	58	1.09
RDX	Radixin	67	1.11
RECK	Reversion Inducing Cysteine Rich Protein With Kazal Motifs	56	0.98
REEP1	Receptor Accessory Protein 1	54	1.2
REG3A	Regenerating Family Member 3 Alpha	54	1.91
REG4	Regenerating Family Member 4	54	4.42
REL	REL Proto-Oncogene, NF-KB Subunit	66	6.73
RELA	RELA Proto-Oncogene, NF-KB Subunit	71	9.35
REN	Renin	68	7.18
RET	Ret Proto-Oncogene	76	19.33
RETN	Resistin	61	3.07
RETNLB	Resistin Like Beta	54	1.21
RETREG1	Reticulophagy Regulator 1	38	6.34
RFC2	Replication Factor C Subunit 2	61	6.72
RFX5	Regulatory Factor X5	56	1.85
RHNO1	RAD9-HUS1-RAD1 Interacting Nuclear Orphan 1	41	3.89
RHOA	Ras Homolog Family Member A	67	3.82
RHOC	Ras Homolog Family Member C	58	1.38
RHOH	Ras Homolog Family Member H	57	2.86
RIMBP2	RIMS Binding Protein 2	53	3.28
RLBP1	Retinaldehyde Binding Protein 1	62	0.91
RLN2	Relaxin 2	54	0.73
RMRP	RNA Component Of Mitochondrial RNA Processing Endoribonuclease	29	4.28
RNASE3	Ribonuclease A Family Member 3	57	12.45
RNASEL	Ribonuclease L	62	3.49
RNASET2	Ribonuclease T2	62	0.57
RNF125	Ring Finger Protein 125	54	5.2
RNF213	Ring Finger Protein 213	54	13.31
RNF39	Ring Finger Protein 39	50	3.93
RNF5	Ring Finger Protein 5	56	3.67
RNPC3	RNA Binding Region (RNP1, RRM) Containing 3	46	7.75
RNPEP	Arginyl Aminopeptidase	58	3.66
RNU4ATAC	RNA, U4atac Small Nuclear (U12-Dependent Splicing)	27	1.92
RORA	RAR Related Orphan Receptor A	66	2.01
RORC	RAR Related Orphan Receptor C	66	5.01
ROS1	ROS Proto-Oncogene 1, Receptor Tyrosine Kinase	67	1.03
RPGR	Retinitis Pigmentosa GTPase Regulator	58	2.7
RPL7	Ribosomal Protein L7	57	2.05
RPS19	Ribosomal Protein S19	63	1.5
RPS27A	Ribosomal Protein S27a	60	8.05
RPS28	Ribosomal Protein S28	52	2.85
RPS6KA1	Ribosomal Protein S6 Kinase A1	71	1.4
RPS6KA3	Ribosomal Protein S6 Kinase A3	73	1.52
RPS6KB1	Ribosomal Protein S6 Kinase B1	72	1.57
RPS9	Ribosomal Protein S9	58	2.6
RPSA	Ribosomal Protein SA	62	3.98
RPTOR	Regulatory Associated Protein Of MTOR Complex 1	62	2.97
RREB1	Ras Responsive Element Binding Protein 1	58	5.16
RRM2B	Ribonucleotide Reductase Regulatory TP53 Inducible Subunit M2B	67	4.36
RSAD2	Radical S-Adenosyl Methionine Domain Containing 2	56	3.89
RSF1	Remodeling And Spacing Factor 1	50	1.03
RSPO1	R-Spondin 1	60	2.36
RTEL1	Regulator Of Telomere Elongation Helicase 1	54	1.77
RTN4	Reticulon 4	63	1.11
RUBCN	RUN And Cysteine Rich Domain Containing Beclin 1 Interacting Protein	41	2.37
RUNX1	Runt Related Transcription Factor 1	67	3.61
RUNX1T1	RUNX1 Translocation Partner 1	57	2.8
RUNX2	Runt Related Transcription Factor 2	65	2.72

RUNX3	Runt Related Transcription Factor 3	61	4.96
RXFP1	Relaxin/Insulin Like Family Peptide Receptor 1	61	1.11
RXRB	Retinoid X Receptor Beta	68	3.51
RYR1	Ryanodine Receptor 1	67	11.91
S100A1	S100 Calcium Binding Protein A1	61	1.27
S100A12	S100 Calcium Binding Protein A12	58	11.18
S100A4	S100 Calcium Binding Protein A4	62	1.51
S100A8	S100 Calcium Binding Protein A8	60	10.56
S100A9	S100 Calcium Binding Protein A9	62	12.05
S100B	S100 Calcium Binding Protein B	67	9.6
S100P	S100 Calcium Binding Protein P	57	0.73
S1PR1	Sphingosine-1-Phosphate Receptor 1	66	0.78
S1PR3	Sphingosine-1-Phosphate Receptor 3	61	1.42
SAA1	Serum Amyloid A1	59	17.83
SAA2	Serum Amyloid A2	50	3.71
SAA4	Serum Amyloid A4, Constitutive	55	4.15
SAG	S-Antigen Visual Arrestin	62	5.05
SAMD9	Sterile Alpha Motif Domain Containing 9	50	3.86
SAMHD1	SAM And HD Domain Containing Deoxynucleoside Triphosphate Triphosphohydrolase 1	59	9.26
SARM1	Sterile Alpha And TIR Motif Containing 1	51	0.57
SAT1	Spermidine/Spermine N1-Acetyltransferase 1	65	6.85
SBDS	SBDS, Ribosome Maturation Factor	58	4.05
SCARB1	Scavenger Receptor Class B Member 1	63	1.4
SCARB2	Scavenger Receptor Class B Member 2	62	4.48
SCARF2	Scavenger Receptor Class F Member 2	54	2.6
SCD	Stearoyl-CoA Desaturase	67	1.03
SCG2	Secretogranin II	57	3.62
SCGB1A1	Secretoglobin Family 1A Member 1	58	6.71
SCN11A	Sodium Voltage-Gated Channel Alpha Subunit 11	60	20.79
SCN1A	Sodium Voltage-Gated Channel Alpha Subunit 1	62	14.83
SCN2B	Sodium Voltage-Gated Channel Beta Subunit 2	62	8.63
SCN4A	Sodium Voltage-Gated Channel Alpha Subunit 4	62	6.82
SCN7A	Sodium Voltage-Gated Channel Alpha Subunit 7	55	2.25
SCN9A	Sodium Voltage-Gated Channel Alpha Subunit 9	64	31.84
SCNN1A	Sodium Channel Epithelial 1 Alpha Subunit	65	1.96
SCNN1B	Sodium Channel Epithelial 1 Beta Subunit	65	1.67
SCT	Secretin	51	7.65
SCYL1	SCY1 Like Pseudokinase 1	57	2.42
SDC1	Syndecan 1	65	1.69
SDCCAG8	Serologically Defined Colon Cancer Antigen 8	54	2.7
SDHA	Succinate Dehydrogenase Complex Flavoprotein Subunit A	66	2.63
SDHB	Succinate Dehydrogenase Complex Iron Sulfur Subunit B	66	17.2
SDHC	Succinate Dehydrogenase Complex Subunit C	61	8.35
SDHD	Succinate Dehydrogenase Complex Subunit D	62	11.3
SEC24C	SEC24 Homolog C, COPII Coat Complex Component	58	4.79
SEC31A	SEC31 Homolog A, COPII Coat Complex Component	58	5.24
SECTM1	Secreted And Transmembrane 1	55	0.57
SELE	Selectin E	69	18.31
SELENON	Selenoprotein N	41	3.34
SELL	Selectin L	61	10.11
SELP	Selectin P	66	12.09
SELPLG	Selectin P Ligand	59	2.71
SEMA3C	Semaphorin 3C	59	4.61
SEMA3D	Semaphorin 3D	52	3.98
SEMA4A	Semaphorin 4A	62	0.46
SEMA4D	Semaphorin 4D	67	0.88
SENP8	SUMO/Sentrin Peptidase Family Member, NEDD8 Specific	57	1.11
SEPSECS	Sep (O-Phosphoserine) TRNA:Sec (Selenocysteine) TRNA Synthase	58	2.97
SERAC1	Serine Active Site Containing 1	50	0.93

SERPINA1	Serpin Family A Member 1	70	11.76
SERPINA3	Serpin Family A Member 3	62	8.84
SERPINA6	Serpin Family A Member 6	60	2.19
SERPINA7	Serpin Family A Member 7	56	1.69
SERPINB1	Serpin Family B Member 1	58	4.61
SERPINB2	Serpin Family B Member 2	62	1.67
SERPINB5	Serpin Family B Member 5	62	1.8
SERPINC1	Serpin Family C Member 1	66	11.93
SERPIND1	Serpin Family D Member 1	62	1.29
SERPINE1	Serpin Family E Member 1	72	9.38
SERPINE2	Serpin Family E Member 2	59	0.85
SERPINF1	Serpin Family F Member 1	62	1.81
SERPINF2	Serpin Family F Member 2	61	5.12
SERPING1	Serpin Family G Member 1	66	11.45
SERPINH1	Serpin Family H Member 1	64	7.42
SETBP1	SET Binding Protein 1	54	5.59
SETD2	SET Domain Containing 2	63	1.69
SETX	Senataxin	57	1.8
SF3B1	Splicing Factor 3b Subunit 1	61	8.45
SFRP1	Secreted Frizzled Related Protein 1	66	0.95
SFRP2	Secreted Frizzled Related Protein 2	59	0.85
SFRP5	Secreted Frizzled Related Protein 5	57	0.83
SFTPB	Surfactant Protein B	57	3.62
SFTPC	Surfactant Protein C	58	5.07
SFTPD	Surfactant Protein D	64	8.05
SGCA	Sarcoglycan Alpha	58	1.1
SGCB	Sarcoglycan Beta	54	0.57
SGCG	Sarcoglycan Gamma	58	1.47
SH2D1A	SH2 Domain Containing 1A	65	5.52
SH3TC2	SH3 Domain And Tetratricopeptide Repeats 2	52	3.72
SHANK3	SH3 And Multiple Ankyrin Repeat Domains 3	55	7.55
SHBG	Sex Hormone Binding Globulin	58	5.67
SHH	Sonic Hedgehog	72	2.09
SHOC2	SHOC2, Leucine Rich Repeat Scaffold Protein	57	3.76
SI	Sucrase-Isomaltase	60	2.6
SIGLEC1	Sialic Acid Binding Ig Like Lectin 1	56	1.66
SIGLEC7	Sialic Acid Binding Ig Like Lectin 7	55	0.85
SIGMAR1	Sigma Non-Opioid Intracellular Receptor 1	63	1.97
SIL1	SIL1 Nucleotide Exchange Factor	57	2.31
SIRPA	Signal Regulatory Protein Alpha	64	1.34
SIRT1	Sirtuin 1	72	2.91
SIX3	SIX Homeobox 3	55	2.36
SKAP2	Src Kinase Associated Phosphoprotein 2	55	2.56
SKP2	S-Phase Kinase Associated Protein 2	62	1.69
SLAMF6	SLAM Family Member 6	58	2.05
SLC10A2	Solute Carrier Family 10 Member 2	59	1.12
SLC11A1	Solute Carrier Family 11 Member 1	63	9.41
SLC11A2	Solute Carrier Family 11 Member 2	66	1.44
SLC12A2	Solute Carrier Family 12 Member 2	64	1.89
SLC15A1	Solute Carrier Family 15 Member 1	64	0.66
SLC16A1	Solute Carrier Family 16 Member 1	66	1.42
SLC16A9	Solute Carrier Family 16 Member 9	54	2.6
SLC17A1	Solute Carrier Family 17 Member 1	57	2.6
SLC17A3	Solute Carrier Family 17 Member 3	55	2.6
SLC17A5	Solute Carrier Family 17 Member 5	61	4.93
SLC19A1	Solute Carrier Family 19 Member 1	63	1.11
SLC22A11	Solute Carrier Family 22 Member 11	58	3.13
SLC22A12	Solute Carrier Family 22 Member 12	62	2.6
SLC22A4	Solute Carrier Family 22 Member 4	62	18.73

SLC22A5	Solute Carrier Family 22 Member 5	65	7.31
SLC22A6	Solute Carrier Family 22 Member 6	60	2.82
SLC22A7	Solute Carrier Family 22 Member 7	56	0.74
SLC22A8	Solute Carrier Family 22 Member 8	58	0.89
SLC25A13	Solute Carrier Family 25 Member 13	62	1.74
SLC25A15	Solute Carrier Family 25 Member 15	60	2.7
SLC25A4	Solute Carrier Family 25 Member 4	65	1.65
SLC26A2	Solute Carrier Family 26 Member 2	59	4.37
SLC26A3	Solute Carrier Family 26 Member 3	62	1.12
SLC29A1	Solute Carrier Family 29 Member 1 (Augustine Blood Group)	67	3.76
SLC29A2	Solute Carrier Family 29 Member 2	63	0.81
SLC2A1	Solute Carrier Family 2 Member 1	74	2.13
SLC2A4	Solute Carrier Family 2 Member 4	67	3.62
SLC2A4RG	SLC2A4 Regulator	50	0.85
SLC2A9	Solute Carrier Family 2 Member 9	63	2.6
SLC35A1	Solute Carrier Family 35 Member A1	55	1.47
SLC37A2	Solute Carrier Family 37 Member 2	50	2.36
SLC39A1	Solute Carrier Family 39 Member 1	55	1.2
SLC39A14	Solute Carrier Family 39 Member 14	59	1.26
SLC39A2	Solute Carrier Family 39 Member 2	53	0.66
SLC39A4	Solute Carrier Family 39 Member 4	60	3.8
SLC39A8	Solute Carrier Family 39 Member 8	58	1.83
SLC3A1	Solute Carrier Family 3 Member 1	62	1.87
SLC3A2	Solute Carrier Family 3 Member 2	58	0.73
SLC40A1	Solute Carrier Family 40 Member 1	64	3.72
SLC4A1	Solute Carrier Family 4 Member 1 (Diego Blood Group)	67	1.93
SLC5A2	Solute Carrier Family 5 Member 2	66	2.36
SLC5A8	Solute Carrier Family 5 Member 8	57	0.8
SLC6A19	Solute Carrier Family 6 Member 19	62	3.52
SLC6A3	Solute Carrier Family 6 Member 3	70	6.58
SLC6A4	Solute Carrier Family 6 Member 4	69	5.86
SLC6A6	Solute Carrier Family 6 Member 6	62	0.57
SLC7A10	Solute Carrier Family 7 Member 10	56	0.57
SLC7A11	Solute Carrier Family 7 Member 11	61	2.36
SLC7A7	Solute Carrier Family 7 Member 7	63	1.47
SLC9A1	Solute Carrier Family 9 Member A1	70	2.01
SLC9A3	Solute Carrier Family 9 Member A3	64	4.45
SLC9A6	Solute Carrier Family 9 Member A6	62	0.54
SLC9A9	Solute Carrier Family 9 Member A9	53	0.93
SLCO1A2	Solute Carrier Organic Anion Transporter Family Member 1A2	59	2.34
SLCO1B1	Solute Carrier Organic Anion Transporter Family Member 1B1	66	2.67
SLCO2A1	Solute Carrier Organic Anion Transporter Family Member 2A1	60	15.93
SLCO2B1	Solute Carrier Organic Anion Transporter Family Member 2B1	62	0.74
SLCO4A1	Solute Carrier Organic Anion Transporter Family Member 4A1	60	0.57
SLEB3	Systemic Lupus Erythematosus Susceptibility 3	7	2.05
SLEB4	Systemic Lupus Erythematosus, Susceptibility To, 4	4	2.05
SLEN1	Systemic Lupus Erythematosus With Nephritis 1	7	2.05
SLIT2	Slit Guidance Ligand 2	64	2.26
SLPI	Secretory Leukocyte Peptidase Inhibitor	57	8.5
SLURP1	Secreted LY6/PLAUR Domain Containing 1	54	2.56
SMAD1	SMAD Family Member 1	63	0.93
SMAD2	SMAD Family Member 2	68	2.33
SMAD3	SMAD Family Member 3	70	3.8
SMAD4	SMAD Family Member 4	70	13.63
SMAD7	SMAD Family Member 7	63	4.34
SMARCA2	SWI/SNF Related, Matrix Associated, Regulator Of Chromatin, Subfamily A, Member 2	66	1.77
SMARCAL1	SWI/SNF Related, Matrix Associated, Regulator Of Chromatin, Subfamily A Like 1	58	1.08
SMG1	SMG1, Nonsense Mediated MRNA Decay Associated PI3K Related Kinase	55	0.51
SMPD1	Sphingomyelin Phosphodiesterase 1	66	1.11

SMUG1	Single-Strand-Selective Monofunctional Uracil-DNA Glycosylase 1	60	7.38
SNAI1	Snail Family Transcriptional Repressor 1	67	3.54
SNCA	Synuclein Alpha	70	18.52
SNRNP70	Small Nuclear Ribonucleoprotein U1 Subunit 70	56	4.73
SNRPB	Small Nuclear Ribonucleoprotein Polypeptides B And B1	60	3.51
SNRPD1	Small Nuclear Ribonucleoprotein D1 Polypeptide	55	3.51
SNRPD3	Small Nuclear Ribonucleoprotein D3 Polypeptide	53	3.67
SNRPE	Small Nuclear Ribonucleoprotein Polypeptide E	59	4.6
SNRPN	Small Nuclear Ribonucleoprotein Polypeptide N	58	6.22
SNX10	Sorting Nexin 10	54	4.97
SNX9	Sorting Nexin 9	57	2.05
SOAT1	Sterol O-Acyltransferase 1	62	1.06
SOCS1	Suppressor Of Cytokine Signaling 1	60	1.64
SOCS3	Suppressor Of Cytokine Signaling 3	64	4.21
SOD1	Superoxide Dismutase 1	74	14.96
SOD2	Superoxide Dismutase 2	71	7.02
SOD3	Superoxide Dismutase 3	58	3.33
SORL1	Sortilin Related Receptor 1	60	1.57
SOS1	SOS Ras/Rac Guanine Nucleotide Exchange Factor 1	66	1.07
SOST	Sclerostin	65	2.5
SOX17	SRY-Box 17	62	7.05
SOX18	SRY-Box 18	53	1.74
SOX9	SRY-Box 9	66	1.44
SP1	Sp1 Transcription Factor	66	2.04
SP100	SP100 Nuclear Antigen	60	2.97
SP140	SP140 Nuclear Body Protein	52	4.29
SP7	Sp7 Transcription Factor	58	0.95
SPAG1	Sperm Associated Antigen 1	52	2.57
SPARC	Secreted Protein Acidic And Cysteine Rich	70	7.94
SPI1	Spi-1 Proto-Oncogene	61	3.45
SPINK1	Serine Peptidase Inhibitor, Kazal Type 1	61	8.04
SPINK5	Serine Peptidase Inhibitor, Kazal Type 5	59	5.89
SPINT2	Serine Peptidase Inhibitor, Kunitz Type 2	60	0.73
SPN	Sialophorin	56	2.65
SPP1	Secreted Phosphoprotein 1	68	9.07
SPRED1	Sprouty Related EVH1 Domain Containing 1	60	1.11
SPRN	Shadow Of Prion Protein	46	2.6
SPRR3	Small Proline Rich Protein 3	51	0.95
SPRY2	Sprouty RTK Signaling Antagonist 2	63	0.57
SPRY4	Sprouty RTK Signaling Antagonist 4	58	0.57
SPTAN1	Spectrin Alpha, Non-Erythrocytic 1	66	2.36
SPTLC1	Serine Palmitoyltransferase Long Chain Base Subunit 1	64	20.03
SPTLC2	Serine Palmitoyltransferase Long Chain Base Subunit 2	66	8.18
SQSTM1	Sequestosome 1	70	14.8
SRC	SRC Proto-Oncogene, Non-Receptor Tyrosine Kinase	75	10.39
SRCAP	Snf2 Related CREBBP Activator Protein	55	1.84
SRD5A1	Steroid 5 Alpha-Reductase 1	61	1.88
SREBF1	Sterol Regulatory Element Binding Transcription Factor 1	62	0.73
SREBF2	Sterol Regulatory Element Binding Transcription Factor 2	62	0.83
SRF	Serum Response Factor	59	0.88
SRGN	Serglycin	54	1.4
SRSF6	Serine And Arginine Rich Splicing Factor 6	53	3.93
SRY	Sex Determining Region Y	54	2.33
SSB	Sjogren Syndrome Antigen B	56	5.25
SSNA1	SS Nuclear Autoantigen 1	50	2.05
SSSCA1	Sjogren Syndrome/Scleroderma Autoantigen 1	49	2.05
SST	Somatostatin	62	11.39
SSTR2	Somatostatin Receptor 2	67	1.03
ST14	Suppression Of Tumorigenicity 14	66	1.03

ST3GAL6	ST3 Beta-Galactoside Alpha-2,3-Sialyltransferase 6	58	0.57
STAB2	Stabilin 2	55	3.67
STAR	Steroidogenic Acute Regulatory Protein	63	1.03
STARD13	StAR Related Lipid Transfer Domain Containing 13	54	0.73
STAT1	Signal Transducer And Activator Of Transcription 1	75	13.71
STAT3	Signal Transducer And Activator Of Transcription 3	73	21.42
STAT4	Signal Transducer And Activator Of Transcription 4	63	28.03
STAT5A	Signal Transducer And Activator Of Transcription 5A	67	1.96
STAT5B	Signal Transducer And Activator Of Transcription 5B	70	2.87
STAT6	Signal Transducer And Activator Of Transcription 6	69	6.16
STEAP4	STEAP4 Metalloreductase	56	5.07
STIM1	Stromal Interaction Molecule 1	70	9.56
STK10	Serine/Threonine Kinase 10	62	0.73
STK11	Serine/Threonine Kinase 11	69	4.36
STK4	Serine/Threonine Kinase 4	67	0.91
STS	Steroid Sulfatase	62	1.88
STUB1	STIP1 Homology And U-Box Containing Protein 1	65	3.21
STX16	Syntaxin 16	58	5.82
STYK1	Serine/Threonine/Tyrosine Kinase 1	58	3.15
SULT1A1	Sulfotransferase Family 1A Member 1	62	3.66
SULT1A2	Sulfotransferase Family 1A Member 2	58	0.6
SULT1A3	Sulfotransferase Family 1A Member 3	53	7.82
SULT1B1	Sulfotransferase Family 1B Member 1	60	2.9
SULT1E1	Sulfotransferase Family 1E Member 1	62	1.75
SULT2A1	Sulfotransferase Family 2A Member 1	64	3.84
SUMO1	Small Ubiquitin-Like Modifier 1	66	1.1
SUMO4	Small Ubiquitin-Like Modifier 4	53	0.57
SUOX	Sulfite Oxidase	64	2.14
SYK	Spleen Associated Tyrosine Kinase	73	5.07
SYNPO	Synaptopodin	58	1.03
SYP	Synaptophysin	62	3.61
T	T Brachyury Transcription Factor	60	2.17
TAC1	Tachykinin Precursor 1	60	18.74
TACR1	Tachykinin Receptor 1	67	8.81
TACR2	Tachykinin Receptor 2	66	2.59
TACR3	Tachykinin Receptor 3	66	1.5
TACSTD2	Tumor Associated Calcium Signal Transducer 2	61	3.53
TAGAP	T-Cell Activation RhoGTPase Activating Protein	53	4.94
TALDO1	Transaldolase 1	64	2.97
TAP1	Transporter 1, ATP Binding Cassette Subfamily B Member	65	1.91
TAP2	Transporter 2, ATP Binding Cassette Subfamily B Member	58	4.24
TAPBP	TAP Binding Protein	59	6.14
TARDBP	TAR DNA Binding Protein	62	14.11
TARS	Threonyl-TRNA Synthetase	61	3.32
TAT	Tyrosine Aminotransferase	62	8.42
TAZ	Tafazzin	61	3.05
TBK1	TANK Binding Kinase 1	69	5.03
TBL2	Transducin Beta Like 2	53	6.72
TBX1	T-Box 1	56	5.16
TBX19	T-Box 19	58	1.62
TBX21	T-Box 21	59	4.12
TBXAS1	Thromboxane A Synthase 1	69	1.64
TCF3	Transcription Factor 3	64	5.9
TCF4	Transcription Factor 4	64	7.73
TCF7	Transcription Factor 7	62	0.93
TCF7L2	Transcription Factor 7 Like 2	64	1.71
TCHH	Trichohyalin	46	3.14
TCIRG1	T-Cell Immune Regulator 1, ATPase H+ Transporting V0 Subunit A3	60	5.41
TCN2	Transcobalamin 2	61	1.2

TDGF1	Teratocarcinoma-Derived Growth Factor 1	62	0.95
TECPR2	Tectonin Beta-Propeller Repeat Containing 2	47	3.39
TEK	TEK Receptor Tyrosine Kinase	74	17.92
TERC	Telomerase RNA Component	35	3.28
TERT	Telomerase Reverse Transcriptase	72	5.91
TET2	Tet Methylcytosine Dioxygenase 2	60	10.39
TF	Transferrin	71	8.99
TFCP2	Transcription Factor CP2	57	1.17
TFF1	Trefoil Factor 1	62	1.75
TFF2	Trefoil Factor 2	58	1.41
TFF3	Trefoil Factor 3	56	3.24
TFG	TRK-Fused Gene	61	4.55
TFPI	Tissue Factor Pathway Inhibitor	65	1.04
TFRC	Transferrin Receptor	70	7.55
TG	Thyroglobulin	61	2
TGFA	Transforming Growth Factor Alpha	66	2.37
TGFB1	Transforming Growth Factor Beta 1	75	40.52
TGFB2	Transforming Growth Factor Beta 2	69	8.66
TGFB3	Transforming Growth Factor Beta 3	71	6.09
TGFBI	Transforming Growth Factor Beta Induced	64	3.75
TGFBR1	Transforming Growth Factor Beta Receptor 1	76	2.64
TGFBR2	Transforming Growth Factor Beta Receptor 2	74	5.37
TGIF1	TGFB Induced Factor Homeobox 1	62	4.09
TGM1	Transglutaminase 1	62	2.46
TGM2	Transglutaminase 2	68	6.18
TH	Tyrosine Hydroxylase	73	4.33
THBD	Thrombomodulin	62	13.4
THBS1	Thrombospondin 1	66	8.36
THBS2	Thrombospondin 2	64	13.92
THBS3	Thrombospondin 3	58	1.9
THBS4	Thrombospondin 4	62	6.9
THEM5	Thioesterase Superfamily Member 5	50	2.9
THPO	Thrombopoietin	62	5.26
THY1	Thy-1 Cell Surface Antigen	65	0.66
TIA1	TIA1 Cytotoxic Granule Associated RNA Binding Protein	54	3.59
TICAM1	Toll Like Receptor Adaptor Molecule 1	62	1.69
TIMP1	TIMP Metallopeptidase Inhibitor 1	67	12.69
TIMP2	TIMP Metallopeptidase Inhibitor 2	63	6.32
TIMP3	TIMP Metallopeptidase Inhibitor 3	65	3.87
TINF2	TERF1 Interacting Nuclear Factor 2	57	2.05
TIRAP	TIR Domain Containing Adaptor Protein	57	11.86
TJP1	Tight Junction Protein 1	62	2.82
TJP2	Tight Junction Protein 2	63	1.03
TKT	Transketolase	65	4.95
TLR1	Toll Like Receptor 1	68	7.68
TLR10	Toll Like Receptor 10	59	2.59
TLR2	Toll Like Receptor 2	72	31.46
TLR3	Toll Like Receptor 3	73	9.21
TLR4	Toll Like Receptor 4	73	50.63
TLR5	Toll Like Receptor 5	67	18.87
TLR6	Toll Like Receptor 6	64	8.45
TLR7	Toll Like Receptor 7	68	5.92
TLR8	Toll Like Receptor 8	69	2.35
TLR9	Toll Like Receptor 9	68	10.58
TMC6	Transmembrane Channel Like 6	57	3.49
TMC8	Transmembrane Channel Like 8	55	3.49
TMEM173	Transmembrane Protein 173	58	6.26
TMEM201	Transmembrane Protein 201	49	2.05
TMEM79	Transmembrane Protein 79	50	2.6

TMSB4X	Thymosin Beta 4, X-Linked	58	2.21
TMTC1	Transmembrane And Tetratricopeptide Repeat Containing 1	51	3.76
TNC	Tenascin C	66	1.55
TNF	Tumor Necrosis Factor	77	79.24
TNFAIP6	TNF Alpha Induced Protein 6	58	6.61
TNFRSF10A	TNF Receptor Superfamily Member 10a	65	7.38
TNFRSF10B	TNF Receptor Superfamily Member 10b	69	6.23
TNFRSF10D	TNF Receptor Superfamily Member 10d	62	1.33
TNFRSF11A	TNF Receptor Superfamily Member 11a	67	20.59
TNFRSF11B	TNF Receptor Superfamily Member 11b	69	26.39
TNFRSF13B	TNF Receptor Superfamily Member 13B	67	5.44
TNFRSF13C	TNF Receptor Superfamily Member 13C	65	8.95
TNFRSF14	TNF Receptor Superfamily Member 14	63	3.63
TNFRSF17	TNF Receptor Superfamily Member 17	64	0.81
TNFRSF1A	TNF Receptor Superfamily Member 1A	70	46.81
TNFRSF1B	TNF Receptor Superfamily Member 1B	69	21.15
TNFRSF21	TNF Receptor Superfamily Member 21	66	0.93
TNFRSF25	TNF Receptor Superfamily Member 25	62	3.91
TNFRSF6B	TNF Receptor Superfamily Member 6b	61	1.38
TNFRSF8	TNF Receptor Superfamily Member 8	64	5.28
TNFRSF9	TNF Receptor Superfamily Member 9	65	1.16
TNFSF10	TNF Superfamily Member 10	68	3.89
TNFSF11	TNF Superfamily Member 11	73	29.83
TNFSF12	TNF Superfamily Member 12	59	6.26
TNFSF13	TNF Superfamily Member 13	64	4.79
TNFSF13B	TNF Superfamily Member 13b	68	8.54
TNFSF14	TNF Superfamily Member 14	59	1.06
TNFSF15	TNF Superfamily Member 15	64	7.19
TNFSF4	TNF Superfamily Member 4	59	11.08
TNFSF8	TNF Superfamily Member 8	58	3.32
TNIP1	TNFAIP3 Interacting Protein 1	57	7.49
TNNI2	Troponin I2, Fast Skeletal Type	62	0.57
TNNI3	Troponin I3, Cardiac Type	69	15.16
TNNT1	Troponin T1, Slow Skeletal Type	61	3.67
TNNT2	Troponin T2, Cardiac Type	67	6.17
TNPO1	Transportin 1	55	1.25
TNXB	Tenascin XB	60	11.3
TOMM40	Translocase Of Outer Mitochondrial Membrane 40	55	4.59
TOP2A	Topoisomerase (DNA) II Alpha	71	3.47
TOR1AIP2	Torsin 1A Interacting Protein 2	49	0.95
TP53	Tumor Protein P53	77	33.33
TP63	Tumor Protein P63	66	8.89
TP73	Tumor Protein P73	64	2.98
TPI1	Triosephosphate Isomerase 1	68	1.72
TPM2	Tropomyosin 2 (Beta)	63	3.16
TPM3	Tropomyosin 3	66	12.44
TPM4	Tropomyosin 4	56	9.82
TPMT	Thiopurine S-Methyltransferase	67	10.75
TPO	Thyroid Peroxidase	67	2.62
TPPP3	Tubulin Polymerization Promoting Protein Family Member 3	52	0.95
TPSAB1	Tryptase Alpha/Beta 1	59	0.81
TPT1	Tumor Protein, Translationally-Controlled 1	65	4.19
TRAF1	TNF Receptor Associated Factor 1	62	4.99
TRAF2	TNF Receptor Associated Factor 2	63	1.17
TRAF3	TNF Receptor Associated Factor 3	67	1.4
TRAF3IP2	TRAF3 Interacting Protein 2	60	12.57
TRAF6	TNF Receptor Associated Factor 6	66	3.96
TRAIP	TRAF Interacting Protein	54	0.57
TRAPPC11	Trafficking Protein Particle Complex 11	49	1.65

TRB	T-Cell Receptor Beta Locus	20	1.51
TRD	T-Cell Receptor Delta Locus	16	0.73
TREM1	Triggering Receptor Expressed On Myeloid Cells 1	59	6.01
TREM2	Triggering Receptor Expressed On Myeloid Cells 2	59	6.98
TREX1	Three Prime Repair Exonuclease 1	62	18.4
TRG	T-Cell Receptor Gamma Locus	17	0.66
TRH	Thyrotropin Releasing Hormone	58	3.45
TRIM13	Tripartite Motif Containing 13	51	1.25
TRIM21	Tripartite Motif Containing 21	60	10.26
TRIM22	Tripartite Motif Containing 22	53	0.66
TRIM25	Tripartite Motif Containing 25	62	0.85
TRIM32	Tripartite Motif Containing 32	58	2.07
TRIM33	Tripartite Motif Containing 33	61	2.36
TRIM39	Tripartite Motif Containing 39	54	3.93
TRIM5	Tripartite Motif Containing 5	62	1.23
TRIP10	Thyroid Hormone Receptor Interactor 10	57	2.05
TROVE2	TROVE Domain Family Member 2	54	4.79
TRPA1	Transient Receptor Potential Cation Channel Subfamily A Member 1	66	17.21
TRPM3	Transient Receptor Potential Cation Channel Subfamily M Member 3	57	1.29
TRPM8	Transient Receptor Potential Cation Channel Subfamily M Member 8	61	3.42
TRPV1	Transient Receptor Potential Cation Channel Subfamily V Member 1	68	25.92
TRPV2	Transient Receptor Potential Cation Channel Subfamily V Member 2	59	0.75
TRPV3	Transient Receptor Potential Cation Channel Subfamily V Member 3	59	1.81
TRPV4	Transient Receptor Potential Cation Channel Subfamily V Member 4	66	7.91
TSC2	Tuberous Sclerosis 2	69	5.16
TSG101	Tumor Susceptibility 101	62	2.88
TSHR	Thyroid Stimulating Hormone Receptor	66	1.03
TSLP	Thymic Stromal Lymphopoietin	62	7.25
TSN	Translin	57	2.97
TSPAN12	Tetraspanin 12	59	0.57
TSPAN2	Tetraspanin 2	53	1.34
TSPAN31	Tetraspanin 31	52	2.97
TSPAN33	Tetraspanin 33	50	0.95
TSPO	Translocator Protein	61	2.23
TTF2	Transcription Termination Factor 2	54	1.82
TTN	Titin	66	12.98
TTR	Transthyretin	70	24.01
TUBA1B	Tubulin Alpha 1b	61	0.85
TUBB	Tubulin Beta Class I	72	0.57
TUBB3	Tubulin Beta 3 Class III	73	2.36
TUSC2	Tumor Suppressor Candidate 2	48	0.81
TWIST1	Twist Family BHLH Transcription Factor 1	63	1.71
TXK	TXK Tyrosine Kinase	63	4.65
TXN	Thioredoxin	66	4.01
TYK2	Tyrosine Kinase 2	75	8.18
TYMP	Thymidine Phosphorylase	65	5.74
TYMS	Thymidylate Synthetase	70	4.92
TYR	Tyrosinase	69	5.65
TYROBP	TYRO Protein Tyrosine Kinase Binding Protein	60	4.6
UBA52	Ubiquitin A-52 Residue Ribosomal Protein Fusion Product 1	58	2.05
UBAC2	UBA Domain Containing 2	50	15.56
UBASH3A	Ubiquitin Associated And SH3 Domain Containing A	55	3.14
UBB	Ubiquitin B	62	4.59
UBD	Ubiquitin D	58	0.88
UBE2L3	Ubiquitin Conjugating Enzyme E2 L3	63	0.73
UBE4A	Ubiquitination Factor E4A	54	3.96
UBQLN4	Ubiquilin 4	56	0.57
UCHL1	Ubiquitin C-Terminal Hydrolase L1	70	7.45
UCN	Urocortin	51	5.64

UCN2	Urocortin 2	48	1.56
UCP1	Uncoupling Protein 1	63	1.74
UCP2	Uncoupling Protein 2	64	1.51
UFD1	Ubiquitin Recognition Factor In ER Associated Degradation 1	44	4.79
UGCG	UDP-Glucose Ceramide Glucosyltransferase	62	1.11
UGT1A	UDP Glucuronosyltransferase Family 1 Member A Complex Locus	12	2.54
UGT1A1	UDP Glucuronosyltransferase Family 1 Member A1	67	4.09
UGT1A10	UDP Glucuronosyltransferase Family 1 Member A10	53	2.29
UGT1A3	UDP Glucuronosyltransferase Family 1 Member A3	53	0.71
UGT1A4	UDP Glucuronosyltransferase Family 1 Member A4	57	0.98
UGT1A6	UDP Glucuronosyltransferase Family 1 Member A6	57	2.28
UGT1A7	UDP Glucuronosyltransferase Family 1 Member A7	52	2.35
UGT1A9	UDP Glucuronosyltransferase Family 1 Member A9	57	1.22
UGT2B4	UDP Glucuronosyltransferase Family 2 Member B4	57	0.96
UGT2B7	UDP Glucuronosyltransferase Family 2 Member B7	65	3.39
UMOD	Uromodulin	60	6.38
UNC119	Unc-119 Lipid Binding Chaperone	57	3.54
UNC13D	Unc-13 Homolog D	58	0.57
UNC93B1	Unc-93 Homolog B1 (C. Elegans)	54	3.13
UROS	Uroporphyrinogen III Synthase	59	3.08
USF1	Upstream Transcription Factor 1	61	0.66
USP5	Ubiquitin Specific Peptidase 5	58	0.97
USP8	Ubiquitin Specific Peptidase 8	67	6.41
UST	Uronyl 2-Sulfotransferase	53	4.11
UTRN	Utrophin	57	3.72
UTS2	Urotensin 2	57	1.32
VANGL1	VANGL Planar Cell Polarity Protein 1	57	5.72
VAPB	VAMP Associated Protein B And C	61	7.16
VASP	Vasodilator-Stimulated Phosphoprotein	62	0.51
VCAM1	Vascular Cell Adhesion Molecule 1	67	15.36
VCAN	Versican	65	2.05
VCL	Vinculin	67	2.91
VCP	Valosin Containing Protein	70	16.29
VDI	Vesicular Stomatitis Virus Defective Interfering Particle Suppression	8	2.05
VDR	Vitamin D Receptor	73	18.97
VEGFA	Vascular Endothelial Growth Factor A	75	16.59
VEGFC	Vascular Endothelial Growth Factor C	67	6.21
VEGFD	Vascular Endothelial Growth Factor D	46	1.49
VIM	Vimentin	70	11.34
VIP	Vasoactive Intestinal Peptide	63	8.19
VNN1	Vanin 1	62	1.55
VPREB1	V-Set Pre-B Cell Surrogate Light Chain 1	55	2.05
VPS33B	VPS33B, Late Endosome And Lysosome Associated	55	1.62
VRK1	Vaccinia Related Kinase 1	66	0.85
VSX1	Visual System Homeobox 1	52	7.13
VTN	Vitronectin	63	9.09
VWF	Von Willebrand Factor	68	10.32
WARS	Tryptophanyl-TRNA Synthetase	65	0.95
WAS	Wiskott-Aldrich Syndrome	69	10.42
WASF1	WAS Protein Family Member 1	61	2.05
WASF2	WAS Protein Family Member 2	58	2.05
WASF3	WAS Protein Family Member 3	57	2.8
WASF4P	WAS Protein Family Member 4, Pseudogene	17	2.05
WASF5P	WAS Protein Family Member 5, Pseudogene	16	2.05
WASH3P	WAS Protein Family Homolog 3 Pseudogene	28	2.05
WASH6P	WAS Protein Family Homolog 6 Pseudogene	19	2.05
WASL	Wiskott-Aldrich Syndrome Like	59	2.05
WDFY2	WD Repeat And FYVE Domain Containing 2	47	3.41
WDR19	WD Repeat Domain 19	51	1.47

WG	Wegener Granulomatosis	6	7.48
WHRN	Whirlin	41	2.9
WIPF1	WAS/WASL Interacting Protein Family Member 1	59	9.6
WISP3	WNT1 Inducible Signaling Pathway Protein 3	58	14.79
WNT1	Wnt Family Member 1	66	3.87
WNT10A	Wnt Family Member 10A	66	2.31
WNT2	Wnt Family Member 2	63	0.73
WNT5A	Wnt Family Member 5A	69	1.64
WT1	Wilms Tumor 1	70	6.42
WWOX	WW Domain Containing Oxidoreductase	62	4.1
WWP1	WW Domain Containing E3 Ubiquitin Protein Ligase 1	60	0.73
XBP1	X-Box Binding Protein 1	64	1.33
XDH	Xanthine Dehydrogenase	67	6.94
XIAP	X-Linked Inhibitor Of Apoptosis	69	4.32
XK	X-Linked Kx Blood Group	57	3.28
XPA	XPA, DNA Damage Recognition And Repair Factor	62	5.01
XPC	XPC Complex Subunit, DNA Damage Recognition And Repair Factor	63	5.54
XPNPEP1	X-Prolyl Aminopeptidase 1	59	2.05
XPNPEP3	X-Prolyl Aminopeptidase 3	56	1.47
XPO1	Exportin 1	64	0.57
XRCC1	X-Ray Repair Cross Complementing 1	61	3.54
XRCC6	X-Ray Repair Cross Complementing 6	66	3.82
YARS2	Tyrosyl-TRNA Synthetase 2	60	2.6
YBX1	Y-Box Binding Protein 1	56	3.83
YBX3	Y-Box Binding Protein 3	56	3.69
YWHAE	Tyrosine 3-Monooxygenase/Tryptophan 5-Monooxygenase Activation Protein Epsilon	70	0.95
YY1AP1	YY1 Associated Protein 1	53	2.8
ZAP70	Zeta Chain Of T-Cell Receptor Associated Protein Kinase 70	73	8.35
ZMPSTE24	Zinc Metallopeptidase STE24	59	4.49
ZNF148	Zinc Finger Protein 148	52	0.73
ZNF365	Zinc Finger Protein 365	52	1.03
ZNF395	Zinc Finger Protein 395	47	0.85
ZNF469	Zinc Finger Protein 469	45	2.41
ZP3	Zona Pellucida Glycoprotein 3	54	1.12
ZYX	Zyxin	64	0.66
