# Supplementary material for: Transcriptomics Analysis of Porcine Caudal Dorsal Root Ganglia in Tail Amputated Pigs Shows Long-Term Effects on Many Pain-Associated Genes
Source: Front Vet Sci. 2019 Sep 18;6:314. doi: 10.3389/fvets.2019.00314 (PMC6760028; doi:10.3389/fvets.2019.00314)
Supplement: Supplementary Data File 3 — GeneCards neuropathic pain genes list. [file Data_Sheet_3.PDF]

Porcine DRG pain gene analysis
Supplementary Data File 3
GeneCard neuropathic pain genes list

Gene Symbol	Description	Gifts	Relevance score
ABHD12	Abhydrolase Domain Containing 12	55	2.63
ACHE	Acetylcholinesterase (Cartwright Blood Group)	68	4.3
ACO1	Aconitase 1	64	2.6
ACP5	Acid Phosphatase 5, Tartrate Resistant	66	9.03
ACTA1	Actin, Alpha 1, Skeletal Muscle	65	13.47
ACTA2	Actin, Alpha 2, Smooth Muscle, Aorta	67	13.64
ACTB	Actin Beta	70	2.8
ACTG1	Actin Gamma 1	70	1.97
ACTG2	Actin, Gamma 2, Smooth Muscle, Enteric	65	11.34
ACTL7B	Actin Like 7B	49	4.08
ADA	Adenosine Deaminase	71	8.21
ADCY1	Adenylate Cyclase 1	66	2.06
ADCY10	Adenylate Cyclase 10, Soluble	60	2.09
ADCY2	Adenylate Cyclase 2	62	2.06
ADCY3	Adenylate Cyclase 3	62	2.06
ADCY4	Adenylate Cyclase 4	58	2.06
ADCY5	Adenylate Cyclase 5	68	2.31
ADCY6	Adenylate Cyclase 6	63	2.06
ADCY7	Adenylate Cyclase 7	63	2.06
ADCY8	Adenylate Cyclase 8	63	2.06
ADCY9	Adenylate Cyclase 9	62	2.43
ADK	Adenosine Kinase	71	1.51
ADORA1	Adenosine A1 Receptor	68	2.97
ADORA2A	Adenosine A2a Receptor	70	2.99
ADRB1	Adrenoceptor Beta 1	70	3.28
ADRB2	Adrenoceptor Beta 2	73	5.32
AHCY	Adenosylhomocysteinase	70	1.34
AK1	Adenylate Kinase 1	66	1.34
AK2	Adenylate Kinase 2	66	1.34
AK5	Adenylate Kinase 5	58	1.34
AK7	Adenylate Kinase 7	53	1.34
AKR1B1	Aldo-Keto Reductase Family 1 Member B	67	3.4
ALAD	Aminolevulinate Dehydratase	64	15.77
ALAS1	5'-Aminolevulinate Synthase 1	62	6.33
ALAS2	5'-Aminolevulinate Synthase 2	60	5.34
ALS2	ALS2, Alsin Rho Guanine Nucleotide Exchange Factor	61	2.5
APOA1	Apolipoprotein A1	70	17.14
APOA2	Apolipoprotein A2	63	4.81
APP	Amyloid Beta Precursor Protein	74	16.77
AQP4	Aquaporin 4	64	3.69
ARAF	A-Raf Proto-Oncogene, Serine/Threonine Kinase	64	1.34
ARF1	ADP Ribosylation Factor 1	64	1.34
ARF3	ADP Ribosylation Factor 3	58	1.34
ARF4	ADP Ribosylation Factor 4	59	1.34
ARF5	ADP Ribosylation Factor 5	54	1.34
ARF6	ADP Ribosylation Factor 6	66	1.34
ARSA	Arylsulfatase A	65	14.53
AS3MT	Arsenite Methyltransferase	57	1.34
ATF3	Activating Transcription Factor 3	65	2.47
ATF4	Activating Transcription Factor 4	67	2.81
ATP1A1	ATPase Na+/K+ Transporting Subunit Alpha 1	68	1.34
ATP1A2	ATPase Na+/K+ Transporting Subunit Alpha 2	66	4.76
ATP1A3	ATPase Na+/K+ Transporting Subunit Alpha 3	66	2.74
ATP1A4	ATPase Na+/K+ Transporting Subunit Alpha 4	55	1.34
ATP1B1	ATPase Na+/K+ Transporting Subunit Beta 1	65	1.86
ATP1B2	ATPase Na+/K+ Transporting Subunit Beta 2	58	1.34
ATP1B3	ATPase Na+/K+ Transporting Subunit Beta 3	59	1.34
ATP1B4	ATPase Na+/K+ Transporting Family Member Beta 4	51	1.34
ATP7A	ATPase Copper Transporting Alpha	64	3.23
B2M	Beta-2-Microglobulin	71	17.06

Porcine DRG pain gene analysis
Supplementary Data File 3
GeneCard neuropathic pain genes list

BDNF	Brain Derived Neurotrophic Factor	70	13.3
BLVRB	Biliverdin Reductase B	57	1.79
BRAF	B-Raf Proto-Oncogene, Serine/Threonine Kinase	78	4.95
CACNA1A	Calcium Voltage-Gated Channel Subunit Alpha1 A	69	6.78
CACNA1B	Calcium Voltage-Gated Channel Subunit Alpha1 B	70	6.32
CACNA1H	Calcium Voltage-Gated Channel Subunit Alpha1 H	68	2.69
CACNA2D1	Calcium Voltage-Gated Channel Auxiliary Subunit Alpha2delta 1	64	3.8
CACNA2D2	Calcium Voltage-Gated Channel Auxiliary Subunit Alpha2delta 2	58	4.31
CACNA2D3	Calcium Voltage-Gated Channel Auxiliary Subunit Alpha2delta 3	57	3.08
CACNA2D4	Calcium Voltage-Gated Channel Auxiliary Subunit Alpha2delta 4	59	3.06
CALCB	Calcitonin Related Polypeptide Beta	57	1.28
CAMK1	Calcium/Calmodulin Dependent Protein Kinase I	62	2.81
CAMK1G	Calcium/Calmodulin Dependent Protein Kinase IG	58	2.81
CAMK2A	Calcium/Calmodulin Dependent Protein Kinase II Alpha	69	3.08
CAMK2B	Calcium/Calmodulin Dependent Protein Kinase II Beta	69	3.08
CAMK2D	Calcium/Calmodulin Dependent Protein Kinase II Delta	68	3.45
CAMK2G	Calcium/Calmodulin Dependent Protein Kinase II Gamma	69	3.28
CBS	Cystathionine-Beta-Synthase	68	6.05
CCK	Cholecystokinin	59	8.6
CCL18	C-C Motif Chemokine Ligand 18	51	5.63
CCL2	C-C Motif Chemokine Ligand 2	70	7.23
CD160	CD160 Molecule	56	1.83
CD34	CD34 Molecule	65	6.74
CFTR	Cystic Fibrosis Transmembrane Conductance Regulator	70	5.58
CHIA	Chitinase, Acidic	55	5.79
CHIT1	Chitinase 1	62	6.88
CHRM2	Cholinergic Receptor Muscarinic 2	68	4.04
CHRM3	Cholinergic Receptor Muscarinic 3	69	4.41
CHRNA9	Cholinergic Receptor Nicotinic Alpha 9 Subunit	59	1.51
CLCA1	Chloride Channel Accessory 1	59	1.34
CLCA2	Chloride Channel Accessory 2	58	1.86
CLCA3P	Chloride Channel Accessory 3, Pseudogene	34	1.34
CLCA4	Chloride Channel Accessory 4	54	1.97
CLCN1	Chloride Voltage-Gated Channel 1	60	3.59
CLCN2	Chloride Voltage-Gated Channel 2	62	2.43
CLCN3	Chloride Voltage-Gated Channel 3	59	1.34
CLCN4	Chloride Voltage-Gated Channel 4	58	1.86
CLCN5	Chloride Voltage-Gated Channel 5	58	5.19
CLCN6	Chloride Voltage-Gated Channel 6	58	1.34
CLCN7	Chloride Voltage-Gated Channel 7	61	4.11
CLIC1	Chloride Intracellular Channel 1	58	1.71
CLIC2	Chloride Intracellular Channel 2	54	1.34
CLIC3	Chloride Intracellular Channel 3	54	1.34
CLIC4	Chloride Intracellular Channel 4	56	1.34
CLIC5	Chloride Intracellular Channel 5	56	1.34
CLIC6	Chloride Intracellular Channel 6	51	1.34
CLNS1A	Chloride Nucleotide-Sensitive Channel 1A	56	1.34
CMA1	Chymase 1	62	4.81
CMKLR1	Chemerin Chemokine-Like Receptor 1	59	1.28
CNGA1	Cyclic Nucleotide Gated Channel Alpha 1	62	1.59
CNGA3	Cyclic Nucleotide Gated Channel Alpha 3	62	1.59
CNGA4	Cyclic Nucleotide Gated Channel Alpha 4	50	1.34
CNGB1	Cyclic Nucleotide Gated Channel Beta 1	59	1.59
CNGB3	Cyclic Nucleotide Gated Channel Beta 3	56	1.59
CNNM1	Cyclin And CBS Domain Divalent Metal Cation Transport Mediator 1	49	4.08
CNNM2	Cyclin And CBS Domain Divalent Metal Cation Transport Mediator 2	54	4.5
CNNM4	Cyclin And CBS Domain Divalent Metal Cation Transport Mediator 4	53	4.33
CNR1	Cannabinoid Receptor 1	66	5.84
CNR2	Cannabinoid Receptor 2	64	4.01
CNTF	Ciliary Neurotrophic Factor	60	2.6
COMT	Catechol-O-Methyltransferase	71	13.6

CPO	Carboxypeptidase O	52	4.25
CPOX	Coproporphyrinogen Oxidase	61	20.98
CREB1	CAMP Responsive Element Binding Protein 1	69	3.9
CRP	C-Reactive Protein	68	8.52
CTSA	Cathepsin A	63	2.24
CX3CL1	C-X3-C Motif Chemokine Ligand 1	60	1.51
CX3CR1	C-X3-C Motif Chemokine Receptor 1	63	4.19
CXCL13	C-X-C Motif Chemokine Ligand 13	59	2.43
CXCR5	C-X-C Motif Chemokine Receptor 5	62	2.17
CYP2D6	Cytochrome P450 Family 2 Subfamily D Member 6	73	4.99
CYP3A4	Cytochrome P450 Family 3 Subfamily A Member 4	74	3.17
DBH	Dopamine Beta-Hydroxylase	73	15.17
DCAF8	DDB1 And CUL4 Associated Factor 8	50	2.2
DES	Desmin	68	7.17
DNM2	Dynamin 2	71	11.5
DOK2	Docking Protein 2	56	1.28
DPYSL2	Dihydropyrimidinase Like 2	62	3.12
DUSP1	Dual Specificity Phosphatase 1	66	1.34
DYNC1H1	Dynein Cytoplasmic 1 Heavy Chain 1	61	12.75
EDNRB	Endothelin Receptor Type B	70	15.42
EGF	Epidermal Growth Factor	73	16.6
ELK1	ELK1, ETS Transcription Factor	62	2.81
ELP1	Elongator Complex Protein 1	46	15.47
ENPP2	Ectonucleotide Pyrophosphatase/Phosphodiesterase 2	66	2.24
ERBB3	Erb-B2 Receptor Tyrosine Kinase 3	76	13.22
ERO1A	Endoplasmic Reticulum Oxidoreductase 1 Alpha	46	1.34
F2R	Coagulation Factor II Thrombin Receptor	68	3.6
F2RL3	F2R Like Thrombin/Trypsin Receptor 3	63	2.5
FAAH	Fatty Acid Amide Hydrolase	67	3.39
FECH	Ferrochelatase	64	13.76
FGA	Fibrinogen Alpha Chain	66	11.27
FLNA	Filamin A	66	11.77
FOS	Fos Proto-Oncogene, AP-1 Transcription Factor Subunit	71	8.19
GABBR1	Gamma-Aminobutyric Acid Type B Receptor Subunit 1	67	3.12
GAD1	Glutamate Decarboxylase 1	70	3.42
GAD2	Glutamate Decarboxylase 2	66	1.79
GAL	Galanin And GMAP Prepropeptide	62	3.21
GATA1	GATA Binding Protein 1	65	11.84
GBA	Glucosylceramidase Beta	68	14.78
GBA3	Glucosylceramidase Beta 3 (Gene/Pseudogene)	53	5.63
GBE1	1,4-Alpha-Glucan Branching Enzyme 1	59	4.01
GCH1	GTP Cyclohydrolase 1	63	6.53
GDAP1	Ganglioside Induced Differentiation Associated Protein 1	57	15.41
GDNF	Glial Cell Derived Neurotrophic Factor	68	8.13
GFAP	Glial Fibrillary Acidic Protein	69	4.08
GFRA2	GDNF Family Receptor Alpha 2	62	2.32
GHRL	Ghrelin And Obestatin Prepropeptide	65	4.11
GJB1	Gap Junction Protein Beta 1	66	16.41
GLE1	GLE1, RNA Export Mediator	55	3.88
GLRA3	Glycine Receptor Alpha 3	57	2.75
GNAS	GNAS Complex Locus	68	6.68
GPR55	G Protein-Coupled Receptor 55	58	4.13
GPRC5B	G Protein-Coupled Receptor Class C Group 5 Member B	54	1.28
GRIA1	Glutamate Ionotropic Receptor AMPA Type Subunit 1	68	3.91
GRIA2	Glutamate Ionotropic Receptor AMPA Type Subunit 2	69	3.83
GRIA3	Glutamate Ionotropic Receptor AMPA Type Subunit 3	69	2.81
GRIA4	Glutamate Ionotropic Receptor AMPA Type Subunit 4	66	2.81
GRIK1	Glutamate Ionotropic Receptor Kainate Type Subunit 1	63	1.51
GRIN1	Glutamate Ionotropic Receptor NMDA Type Subunit 1	70	5.23
GRIN2A	Glutamate Ionotropic Receptor NMDA Type Subunit 2A	71	8.07
GRIN2B	Glutamate Ionotropic Receptor NMDA Type Subunit 2B	68	6.5

GRIN2C	Glutamate Ionotropic Receptor NMDA Type Subunit 2C	64	4.01
GRIN2D	Glutamate Ionotropic Receptor NMDA Type Subunit 2D	65	5.53
GRIN3A	Glutamate Ionotropic Receptor NMDA Type Subunit 3A	58	1.81
GRINA	Glutamate Ionotropic Receptor NMDA Type Subunit Associated Protein 1	49	2.81
GRM1	Glutamate Metabotropic Receptor 1	70	4.91
GRM2	Glutamate Metabotropic Receptor 2	65	3.08
GRM3	Glutamate Metabotropic Receptor 3	66	2.81
GRM4	Glutamate Metabotropic Receptor 4	65	2.81
GRM5	Glutamate Metabotropic Receptor 5	66	3.44
GRM6	Glutamate Metabotropic Receptor 6	66	2.81
GRM7	Glutamate Metabotropic Receptor 7	64	3.44
GRM8	Glutamate Metabotropic Receptor 8	66	2.81
GSN	Gelsolin	70	6.43
HACD1	3-Hydroxyacyl-CoA Dehydratase 1	46	4.43
HBA1	Hemoglobin Subunit Alpha 1	60	12.66
HCN1	Hyperpolarization Activated Cyclic Nucleotide Gated Potassium Channel 1	61	1.28
HEXA	Hexosaminidase Subunit Alpha	64	13.59
HMBS	Hydroxymethylbilane Synthase	66	18.94
HMGB1	High Mobility Group Box 1	64	3.24
HOMER1	Homer Scaffolding Protein 1	57	1.28
HPSE	Heparanase	65	4.55
HPSE2	Heparanase 2 (Inactive)	54	10.67
HRH1	Histamine Receptor H1	67	2.54
HSP90AA1	Heat Shock Protein 90 Alpha Family Class A Member 1	70	2.8
HSPA1A	Heat Shock Protein Family A (Hsp70) Member 1A	62	2.89
HSPA1B	Heat Shock Protein Family A (Hsp70) Member 1B	55	1.34
HSPB1	Heat Shock Protein Family B (Small) Member 1	71	5.52
HSPB3	Heat Shock Protein Family B (Small) Member 3	54	1.99
HSPB8	Heat Shock Protein Family B (Small) Member 8	62	4.47
HTR2C	5-Hydroxytryptamine Receptor 2C	69	2.77
IAPP	Islet Amyloid Polypeptide	56	4.94
IFNA1	Interferon Alpha 1	58	4.23
IFNG	Interferon Gamma	69	11.66
IGHMBP2	Immunoglobulin Mu Binding Protein 2	57	4.5
IL10	Interleukin 10	66	16.53
IL1B	Interleukin 1 Beta	70	11.33
INS	Insulin	67	9.65
KCMF1	Potassium Channel Modulatory Factor 1	50	2.81
KCNA2	Potassium Voltage-Gated Channel Subfamily A Member 2	63	5.6
KCNK1	Potassium Two Pore Domain Channel Subfamily K Member 1	61	2.81
KCNK10	Potassium Two Pore Domain Channel Subfamily K Member 10	58	2.81
KCNK12	Potassium Two Pore Domain Channel Subfamily K Member 12	55	2.81
KCNK13	Potassium Two Pore Domain Channel Subfamily K Member 13	54	2.81
KCNK15	Potassium Two Pore Domain Channel Subfamily K Member 15	52	2.81
KCNK16	Potassium Two Pore Domain Channel Subfamily K Member 16	50	2.81
KCNK17	Potassium Two Pore Domain Channel Subfamily K Member 17	50	3.53
KCNK2	Potassium Two Pore Domain Channel Subfamily K Member 2	63	3.08
KCNK3	Potassium Two Pore Domain Channel Subfamily K Member 3	69	4.43
KCNK4	Potassium Two Pore Domain Channel Subfamily K Member 4	58	4.82
KCNK5	Potassium Two Pore Domain Channel Subfamily K Member 5	59	3.17
KCNK6	Potassium Two Pore Domain Channel Subfamily K Member 6	57	3.83
KCNK7	Potassium Two Pore Domain Channel Subfamily K Member 7	51	2.81
KCNK9	Potassium Two Pore Domain Channel Subfamily K Member 9	64	3.2
KCNRG	Potassium Channel Regulator	45	2.81
KCNT1	Potassium Sodium-Activated Channel Subfamily T Member 1	58	3.44
KCNT2	Potassium Sodium-Activated Channel Subfamily T Member 2	50	2.81
KCNU1	Potassium Calcium-Activated Channel Subfamily U Member 1	48	2.81
KCNV1	Potassium Voltage-Gated Channel Modifier Subfamily V Member 1	54	2.81
KCNV2	Potassium Voltage-Gated Channel Modifier Subfamily V Member 2	55	3.06
KCTD1	Potassium Channel Tetramerization Domain Containing 1	54	3.17
KCTD10	Potassium Channel Tetramerization Domain Containing 10	50	2.81

KCTD11	Potassium Channel Tetramerization Domain Containing 11	50	2.81
KCTD12	Potassium Channel Tetramerization Domain Containing 12	49	2.81
KCTD13	Potassium Channel Tetramerization Domain Containing 13	46	2.81
KCTD14	Potassium Channel Tetramerization Domain Containing 14	45	2.81
KCTD15	Potassium Channel Tetramerization Domain Containing 15	51	2.81
KCTD2	Potassium Channel Tetramerization Domain Containing 2	47	2.81
KCTD3	Potassium Channel Tetramerization Domain Containing 3	47	2.81
KCTD4	Potassium Channel Tetramerization Domain Containing 4	45	2.81
KCTD5	Potassium Channel Tetramerization Domain Containing 5	49	2.81
KCTD6	Potassium Channel Tetramerization Domain Containing 6	45	2.81
KCTD7	Potassium Channel Tetramerization Domain Containing 7	53	3.44
KCTD8	Potassium Channel Tetramerization Domain Containing 8	46	2.81
KCTD9	Potassium Channel Tetramerization Domain Containing 9	49	2.81
KIDINS220	Kinase D Interacting Substrate 220	53	4.92
KIF1B	Kinesin Family Member 1B	60	13.83
KRAS	KRAS Proto-Oncogene, GTPase	70	8.15
KRT20	Keratin 20	58	3.49
L1CAM	L1 Cell Adhesion Molecule	62	5.29
LDB3	LIM Domain Binding 3	57	1.94
LIFR	LIF Receptor Alpha	67	11.44
LIG3	DNA Ligase 3	64	4.25
LPA	Lipoprotein(A)	58	6.54
LPAR1	Lysophosphatidic Acid Receptor 1	66	6.14
LRIG2	Leucine Rich Repeats And Immunoglobulin Like Domains 2	55	10.67
LYZ	Lysozyme	66	10.49
MAG	Myelin Associated Glycoprotein	65	3.17
MAOA	Monoamine Oxidase A	66	3.9
MAP2K1	Mitogen-Activated Protein Kinase Kinase 1	79	2.65
MAP2K2	Mitogen-Activated Protein Kinase Kinase 2	76	2.23
MAP2K3	Mitogen-Activated Protein Kinase Kinase 3	71	1.34
MAP2K4	Mitogen-Activated Protein Kinase Kinase 4	66	1.77
MAP2K5	Mitogen-Activated Protein Kinase Kinase 5	66	2.15
MAP2K6	Mitogen-Activated Protein Kinase Kinase 6	67	1.34
MAPK1	Mitogen-Activated Protein Kinase 1	75	7.53
MAPK10	Mitogen-Activated Protein Kinase 10	72	2.8
MAPK11	Mitogen-Activated Protein Kinase 11	70	1.34
MAPK12	Mitogen-Activated Protein Kinase 12	67	1.62
MAPK13	Mitogen-Activated Protein Kinase 13	67	1.34
MAPK14	Mitogen-Activated Protein Kinase 14	75	2.62
MAPK3	Mitogen-Activated Protein Kinase 3	73	6.43
MAPK4	Mitogen-Activated Protein Kinase 4	58	1.34
MAPK6	Mitogen-Activated Protein Kinase 6	62	2.81
MAPK7	Mitogen-Activated Protein Kinase 7	67	3.21
MAPK8	Mitogen-Activated Protein Kinase 8	74	5.14
MAPK9	Mitogen-Activated Protein Kinase 9	70	1.86
MBTPS2	Membrane Bound Transcription Factor Peptidase, Site 2	58	4.66
MEFV	MEFV, Pyrin Innate Immunity Regulator	59	19.46
MEOX2	Mesenchyme Homeobox 2	54	4.41
MFN2	Mitofusin 2	67	15.35
MIF	Macrophage Migration Inhibitory Factor (Glycosylation-Inhibiting Factor)	68	8.81
MIR132	MicroRNA 132	23	1.28
MPZ	Myelin Protein Zero	61	23.01
MTMR2	Myotubularin Related Protein 2	61	12.92
MT-TL1	Mitochondrially Encoded TRNA Leucine 1 (UUA/G)	21	10.67
MT-TR	Mitochondrially Encoded TRNA Arginine	19	3.35
MTX1	Metaxin 1	55	5.49
MYH11	Myosin Heavy Chain 11	62	11.01
MYH7	Myosin Heavy Chain 7	66	14.52
MYO5A	Myosin VA	59	4.08
MYOM2	Myomesin 2	55	5.79
NDRG1	N-Myc Downstream Regulated 1	62	10.64

NEB	Nebulin	57	1.84
NEDD4	Neural Precursor Cell, Developmentally Down-Regulated 4, E3 Ubiquitin Protein Ligase	65	2.37
NGF	Nerve Growth Factor	70	29.91
NGFR	Nerve Growth Factor Receptor	66	3.55
NOS1	Nitric Oxide Synthase 1	67	4.07
NOS2	Nitric Oxide Synthase 2	68	6.32
NR1H2	Nuclear Receptor Subfamily 1 Group H Member 2	68	2.01
NRTN	Neurturin	60	4.72
NTF3	Neurotrophin 3	65	2.48
NTRK1	Neurotrophic Receptor Tyrosine Kinase 1	75	37.88
NTRK2	Neurotrophic Receptor Tyrosine Kinase 2	75	11.31
OPRK1	Opioid Receptor Kappa 1	65	6.78
OPRM1	Opioid Receptor Mu 1	70	10.77
P2RX1	Purinergic Receptor P2X 1	63	4.93
P2RX2	Purinergic Receptor P2X 2	61	7.4
P2RX3	Purinergic Receptor P2X 3	58	6.9
P2RX4	Purinergic Receptor P2X 4	62	5.22
P2RX7	Purinergic Receptor P2X 7	66	5.6
P2RY1	Purinergic Receptor P2Y1	62	2.19
P2RY12	Purinergic Receptor P2Y12	67	3.31
P2RY2	Purinergic Receptor P2Y2	66	1.28
P2RY4	Pyrimidinergic Receptor P2Y4	59	1.28
P2RY6	Pyrimidinergic Receptor P2Y6	64	1.28
PCBD1	Pterin-4 Alpha-Carbinolamine Dehydratase 1	61	4.81
PDGFB	Platelet Derived Growth Factor Subunit B	70	3
PDIA4	Protein Disulfide Isomerase Family A Member 4	58	1.34
PDPK1	3-Phosphoinositide Dependent Protein Kinase 1	69	1.34
PDYN	Prodynorphin	60	8.67
PICK1	Protein Interacting With PRKCA 1	57	1.28
PIK3R1	Phosphoinositide-3-Kinase Regulatory Subunit 1	70	4.21
PIK3R2	Phosphoinositide-3-Kinase Regulatory Subunit 2	66	3.7
PIK3R3	Phosphoinositide-3-Kinase Regulatory Subunit 3	61	2.81
PIK3R4	Phosphoinositide-3-Kinase Regulatory Subunit 4	65	2.81
PIK3R5	Phosphoinositide-3-Kinase Regulatory Subunit 5	62	3.06
PIP4K2A	Phosphatidylinositol-5-Phosphate 4-Kinase Type 2 Alpha	64	2.07
PIP4K2B	Phosphatidylinositol-5-Phosphate 4-Kinase Type 2 Beta	55	1.34
PIP4K2C	Phosphatidylinositol-5-Phosphate 4-Kinase Type 2 Gamma	54	1.34
PIP5K1A	Phosphatidylinositol-4-Phosphate 5-Kinase Type 1 Alpha	62	1.34
PIP5K1B	Phosphatidylinositol-4-Phosphate 5-Kinase Type 1 Beta	59	1.34
PIP5K1C	Phosphatidylinositol-4-Phosphate 5-Kinase Type 1 Gamma	67	5.78
PITX2	Paired Like Homeodomain 2	64	3.06
PKLR	Pyruvate Kinase, Liver And RBC	66	5.77
PLA2G6	Phospholipase A2 Group VI	67	3.28
PLCB1	Phospholipase C Beta 1	67	3.44
PLCB2	Phospholipase C Beta 2	66	2.81
PLCB3	Phospholipase C Beta 3	67	3.17
PLCB4	Phospholipase C Beta 4	65	3.17
PLCD1	Phospholipase C Delta 1	66	4.32
PLCD3	Phospholipase C Delta 3	59	2.81
PLCD4	Phospholipase C Delta 4	58	2.81
PLCE1	Phospholipase C Epsilon 1	58	3.62
PLCG1	Phospholipase C Gamma 1	67	3.55
PLCG2	Phospholipase C Gamma 2	68	4.9
PLCZ1	Phospholipase C Zeta 1	57	3.44
PLEKHG5	Pleckstrin Homology And RhoGEF Domain Containing G5	56	2.83
PMP22	Peripheral Myelin Protein 22	60	17.6
PNPLA6	Patatin Like Phospholipase Domain Containing 6	61	2.94
PPARG	Peroxisome Proliferator Activated Receptor Gamma	76	6.9
PPOX	Protoporphyrinogen Oxidase	59	15.93
PRKACA	Protein Kinase CAMP-Activated Catalytic Subunit Alpha	71	4.36
PRKACB	Protein Kinase CAMP-Activated Catalytic Subunit Beta	67	3.1

PRKACG	Protein Kinase CAMP-Activated Catalytic Subunit Gamma	66	3.1
PRKAR1A	Protein Kinase CAMP-Dependent Type I Regulatory Subunit Alpha	71	6.28
PRKAR1B	Protein Kinase CAMP-Dependent Type I Regulatory Subunit Beta	63	3.73
PRKAR2A	Protein Kinase CAMP-Dependent Type II Regulatory Subunit Alpha	62	3.1
PRKAR2B	Protein Kinase CAMP-Dependent Type II Regulatory Subunit Beta	62	3.1
PRKCA	Protein Kinase C Alpha	72	3.53
PRKCB	Protein Kinase C Beta	70	2.81
PRKCD	Protein Kinase C Delta	78	4.24
PRKCE	Protein Kinase C Epsilon	72	2.81
PRKCG	Protein Kinase C Gamma	73	13.79
PRKCH	Protein Kinase C Eta	71	4.26
PRKCI	Protein Kinase C Iota	71	3.32
PRKCQ	Protein Kinase C Theta	73	3.17
PRKCZ	Protein Kinase C Zeta	72	2.81
PRKD1	Protein Kinase D1	68	4.32
PRKD3	Protein Kinase D3	66	2.81
PRX	Periaxin	54	4.63
PSAP	Prosaposin	66	7.53
PSEN1	Presenilin 1	74	13.96
PTGS2	Prostaglandin-Endoperoxide Synthase 2	70	16.87
PTH2R	Parathyroid Hormone 2 Receptor	62	1.28
PTPN13	Protein Tyrosine Phosphatase, Non-Receptor Type 13	63	3.88
RAF1	Raf-1 Proto-Oncogene, Serine/Threonine Kinase	79	3.75
REST	RE1 Silencing Transcription Factor	60	4.06
RET	Ret Proto-Oncogene	76	17.49
RGS4	Regulator Of G Protein Signaling 4	62	1.81
RNASEH1	Ribonuclease H1	59	4.43
RYR1	Ryanodine Receptor 1	67	13.28
S100A1	S100 Calcium Binding Protein A1	61	4.55
S100A8	S100 Calcium Binding Protein A8	60	3.29
S100A9	S100 Calcium Binding Protein A9	62	2.47
SAA1	Serum Amyloid A1	59	6.26
SAA2	Serum Amyloid A2	50	3.35
SAA4	Serum Amyloid A4, Constitutive	55	3.35
SCARB2	Scavenger Receptor Class B Member 2	62	14.23
SCN10A	Sodium Voltage-Gated Channel Alpha Subunit 10	67	37.6
SCN11A	Sodium Voltage-Gated Channel Alpha Subunit 11	60	38.19
SCN1A	Sodium Voltage-Gated Channel Alpha Subunit 1	62	9.45
SCN1B	Sodium Voltage-Gated Channel Beta Subunit 1	62	5.53
SCN2A	Sodium Voltage-Gated Channel Alpha Subunit 2	65	4.47
SCN2B	Sodium Voltage-Gated Channel Beta Subunit 2	62	5.01
SCN3A	Sodium Voltage-Gated Channel Alpha Subunit 3	65	4.59
SCN3B	Sodium Voltage-Gated Channel Beta Subunit 3	61	5.49
SCN4A	Sodium Voltage-Gated Channel Alpha Subunit 4	62	7.76
SCN4B	Sodium Voltage-Gated Channel Beta Subunit 4	60	21.36
SCN5A	Sodium Voltage-Gated Channel Alpha Subunit 5	70	6.44
SCN7A	Sodium Voltage-Gated Channel Alpha Subunit 7	55	5.17
SCN8A	Sodium Voltage-Gated Channel Alpha Subunit 8	60	5.06
SCN9A	Sodium Voltage-Gated Channel Alpha Subunit 9	64	43.47
SCNN1A	Sodium Channel Epithelial 1 Alpha Subunit	65	3.11
SCNN1B	Sodium Channel Epithelial 1 Beta Subunit	65	3.11
SCNN1D	Sodium Channel Epithelial 1 Delta Subunit	60	2.81
SCNN1G	Sodium Channel Epithelial 1 Gamma Subunit	64	2.81
SEC61A1	Sec61 Translocon Alpha 1 Subunit	58	1.34
SEC61A2	Sec61 Translocon Alpha 2 Subunit	51	1.34
SEC61B	Sec61 Translocon Beta Subunit	54	1.34
SEC61G	Sec61 Translocon Gamma Subunit	50	1.34
SELENON	Selenoprotein N	41	10.31
SLC12A1	Solute Carrier Family 12 Member 1	66	2.43
SLC12A2	Solute Carrier Family 12 Member 2	64	1.82
SLC12A3	Solute Carrier Family 12 Member 3	66	5.01

Porcine DRG pain gene analysis
Supplementary Data File 3
GeneCard neuropathic pain genes list

SLC12A5	Solute Carrier Family 12 Member 5	66	2.72
SLC12A6	Solute Carrier Family 12 Member 6	66	4.56
SLC18A2	Solute Carrier Family 18 Member A2	64	2.84
SLC1A2	Solute Carrier Family 1 Member 2	68	2.54
SLC6A2	Solute Carrier Family 6 Member 2	67	4.51
SLC6A4	Solute Carrier Family 6 Member 4	69	6.07
SLC6A5	Solute Carrier Family 6 Member 5	63	1.93
SLC6A9	Solute Carrier Family 6 Member 9	66	1.51
SLC9A1	Solute Carrier Family 9 Member A1	70	2.87
SLCO2A1	Solute Carrier Organic Anion Transporter Family Member 2A1	60	12.28
SMN1	Survival Of Motor Neuron 1, Telomeric	58	3.57
SOD1	Superoxide Dismutase 1	74	6.33
SOS1	SOS Ras/Rac Guanine Nucleotide Exchange Factor 1	66	1.86
SOS2	SOS Ras/Rho Guanine Nucleotide Exchange Factor 2	62	1.34
SOX10	SRY-Box 10	62	13.81
SPOCK1	SPARC/Osteonectin, Cwcv And Kazal Like Domains Proteoglycan 1	56	4.25
SRC	SRC Proto-Oncogene, Non-Receptor Tyrosine Kinase	75	6.46
SRD5A1	Steroid 5 Alpha-Reductase 1	61	2.17
SST	Somatostatin	62	5.78
SYP	Synaptophysin	62	9.01
TAC1	Tachykinin Precursor 1	60	11.68
TACR1	Tachykinin Receptor 1	67	8.66
TECPR2	Tectonin Beta-Propeller Repeat Containing 2	47	4.66
TFG	TRK-Fused Gene	61	5.8
THBD	Thrombomodulin	62	4.65
THBS3	Thrombospondin 3	58	5.49
THBS4	Thrombospondin 4	62	9.7
TIMP3	TIMP Metallopeptidase Inhibitor 3	65	1.39
TLR4	Toll Like Receptor 4	73	10.82
TNF	Tumor Necrosis Factor	77	18.74
TNFRSF1A	TNF Receptor Superfamily Member 1A	70	22.36
TP63	Tumor Protein P63	66	5.87
TPM3	Tropomyosin 3	66	11.25
TRPA1	Transient Receptor Potential Cation Channel Subfamily A Member 1	66	12.02
TRPM8	Transient Receptor Potential Cation Channel Subfamily M Member 8	61	3.17
TRPV1	Transient Receptor Potential Cation Channel Subfamily V Member 1	68	20.96
TTR	Transthyretin	70	19.39
TUBB3	Tubulin Beta 3 Class III	73	2.64
TYMP	Thymidine Phosphorylase	65	14.08
UCHL1	Ubiquitin C-Terminal Hydrolase L1	70	3.59
UGCG	UDP-Glucose Ceramide Glucosyltransferase	62	5.49
UPK3A	Uroplakin 3A	55	6.04
UROD	Uroporphyrinogen Decarboxylase	62	14.24
UROS	Uroporphyrinogen III Synthase	59	6.71
USP5	Ubiquitin Specific Peptidase 5	58	1.28
VAPB	VAMP Associated Protein B And C	61	4.23
VCP	Valosin Containing Protein	70	16.83
VEGFA	Vascular Endothelial Growth Factor A	75	5.7
VRK1	Vaccinia Related Kinase 1	66	1.12
VWF	Von Willebrand Factor	68	3.9
WDFY2	WD Repeat And FYVE Domain Containing 2	47	4.68
