# Supplementary material for: Transcriptomics Analysis of Porcine Caudal Dorsal Root Ganglia in Tail Amputated Pigs Shows Long-Term Effects on Many Pain-Associated Genes
Source: Front Vet Sci. 2019 Sep 18;6:314. doi: 10.3389/fvets.2019.00314 (PMC6760028; doi:10.3389/fvets.2019.00314)
Supplement: Supplementary Data File 4 — Cluster A pain genes_3-way ANOVA table. [file Data_Sheet_4.PDF]

Porcine DRG pain genes analysis
Supplementary Data 4

Cluster A (Neuronal function)

3-way ANOVA table of significant, differentially expressed (down-regulated)
Neuropathic pain associated DRG genes after tail amputation

		Test of fixed effects			
	**Statistic**	**Tail treatment**	**Treatment age**	**Time after treatment**	**3-way interaction**
**Gene**	*Num DF*	*1*	*1*	*2*	*2*
	Den DF	*83*	*83*	*83*	*83*
KCNA4	P value	<0.001	0.173	<0.001	0.161
	F ratio	60.04	1.89	11.41	2.00
KCNG4	P value	<0.001	0.001	0.440	0.194
	F ratio	40.84	11.53	0.6460	1.72
KCNQ3	P value	<0.001	0.002	0.136	0.458
	F ratio	49.21	9.87	2.04	1.57
KCNH6	P value	<0.001	<0.001	0.021	0.687
	F ratio	68.33	24.83	4.04	0.16
KCNV1	P value	<0.001	0.017	<0.001	0.980
	F ratio	33.26	5.94	9.41	0.00
KCNT2	P value	<0.001	0.002	0.148	0.380
	F ratio	33.35	10.23	1.96	0.78
KCNK1	P value	<0.001	0.010	0.002	0.966
	F ratio	65.54	0.9216	6.51	0.00
KCNK10	P value	<0.001	0.954	0.224	0.047
	F ratio	39.08	0.00	1.52	4.05
SCN1A	P value	<0.001	0.005	0.006	0.473
	F ratio	45.17	8.40	5.55	0.52
SCN4B	P value	<0.001	0.130	<0.001	0.192
	F ratio	50.04	2.34	8.61	1.73
CACNA1A	P value	<0.001	0.016	0.089	0.410
	F ratio	55.82	6.00	2.49	0.69
CACNA2D2	P value	<0.001	<0.001	<0.001	0.388
	F ratio	74.22	33.26	10.32	0.75
ATP1A1	P value	<0.001	0.019	0.019	0.468
	F ratio	81.83	5.69	4.14	0.53
ASIC1	P value	<0.001	0.007	0.233	0.133
	F ratio	26.36	7.77	1.48	2.30
SLCO5A1	P value	<0.001	0.099	0.099	0.210
	F ratio	29.13	2.79	2.37	1.59
SLC8A2	P value	<0.001	0.267	0.039	0.560
	F ratio	18.57	1.25	3.38	0.34
SLC9A9	P value	<0.001	0.415	<0.001	0.679
	F ratio	57.95	0.67	11.67	0.17
SLC17A8	P value	<0.001	<0.001	0.090	0.260
	F ratio	16.91	63.01	2.48	1.29
SLC24A2	P value	<0.001	0.955	<0.001	0.202
	F ratio	54.91	0.00	10.46	1.66
SLC25A22	P value	<0.001	0.005	0.115	0.966
	F ratio	25.21	8.29	2.22	0.00

GABRB2	P value	<0.001	0.081	0.230	0.162
	F ratio	94.05	3.12	1.49	1.99
GABRB3	P value	<0.001	0.651	0.071	0.818
	F ratio	53.00	0.21	2.74	0.05
GABBR1	P value	<0.001	0.023	0.232	0.328
	F ratio	40.58	5.40	1.49	0.97
GABBR2	P value	<0.001	0.003	0.170	0.795
	F ratio	53.79	9.44	1.81	0.07
GRM4	P value	<0.001	0.006	0.132	0.294
	F ratio	43.89	7.95	2.08	1.12
GRM8	P value	<0.001	0.510	0.105	0.8510
	F ratio	15.30	0.44	2.31	0.04
CHRNA3	P value	<0.001	0.012	0.005	0.810
	F ratio	47.84	6.62	5.63	0.06
CHRNB4	P value	<0.001	0.004	0.186	0.522
	F ratio	45.64	8.59	1.72	0.41
OPRM1	P value	<0.001	0.004	0.095	0.932
	F ratio	35.66	9.00	2.43	0.01
ADORA1	P value	<0.001	0.272	0.094	0.424
	F ratio	62.78	1.22	2.44	0.65
P2RY14	P value	<0.001	<0.001	0.802	0.102
	F ratio	76.89	18.18	0.22	2.73
