# Supplementary material for: Transcriptomics Analysis of Porcine Caudal Dorsal Root Ganglia in Tail Amputated Pigs Shows Long-Term Effects on Many Pain-Associated Genes
Source: Front Vet Sci. 2019 Sep 18;6:314. doi: 10.3389/fvets.2019.00314 (PMC6760028; doi:10.3389/fvets.2019.00314)
Supplement: Supplementary Data File 5 — Cluster B pain genes_3-way ANOVA table. [file Data_Sheet_5.PDF]

Porcine DRG pain genes analysis
Supplementary Data 5

Cluster B (Wound healing)

3-way ANOVA table of significant, differentially expressed (up-regulated) Neuropathic pain associated DRG genes after tail amputation

		Test of fixed effects			
	**Statistic**	**Tail treatment**	**Treatment age**	**Time after treatment**	**3-way interaction**
**Gene**	*Num DF*	*1*	*1*	*2*	*2*
	Den DF	*83*	*83*	*83*	*83*
ATF3	P value	<0.001	<0.001	0.147	0.041
	F ratio	879.07	34.53	1.96	4.30
JUN	P value	<0.001	0.510	<0.001	0.201
	F ratio	134.63	0.44	11.28	1.66
PLA2G3	P value	<0.001	0.117	0.156	0.717
	F ratio	47.11	2.52	1.90	0.13
PTGS2	P value	<0.001	<0.001	0.004	0.166
	F ratio	500.81	22.66	6.01	1.96
SERPINB2	P value	<0.001	0.016	<0.001	0.690
	F ratio	673.44	6.10	19.60	0.16
TIMP1	P value	<0.001	0.016	<0.001	0.067
	F ratio	115.99	6.08	31.94	3.46
PENK	P value	<0.001	0.997	0.003	0.023
	F ratio	529.25	0.00	6.46	5.34
GAL	P value	<0.001	0.861	0.725	0.168
	F ratio	492.03	0.03	0.32	1.94
SST	P value	<0.001	0.060	0.695	0.942
	F ratio	230.96	3.64	0.37	0.01
NPY	P value	<0.001	0.450	<0.001	0.504
	F ratio	292.97	0.58	17.73	0.45
IL24	P value	<0.001	0.653	<0.001	0.791
	F ratio	209.42	0.20	65.84	0.07
ADCYAP1	P value	<0.001	0.003	0.004	0.055
	F ratio	445.78	9.19	6.04	3.78
HTR2A	P value	<0.001	0.624	0.717	0.036
	F ratio	132.17	0.24	0.33	4.55
AGTR1	P value	<0.001	<0.001	<0.001	0.802
	F ratio	98.94	20.82	10.42	0.06
NMUR2	P value	<0.001	0.205	0.606	0.713
	F ratio	162.15	1.64	0.50	0.14
NPSR1	P value	<0.001	0.015	0.012	0.006
	F ratio	312.87	6.13	4.71	8.02
NTRK3	P value	<0.001	0.039	0.332	0.546
	F ratio	80.00	4.40	1.12	0.37
IL4R	P value	<0.001	<0.001	0.001	0.839
	F ratio	145.06	21.44	10.12	0.04
KCNK3	P value	<0.001	0.545	0.198	0.631
	F ratio	71.42	0.37	1.65	0.23
SLC1A1	P value	<0.001	0.004	<0.001	0.038
	F ratio	91.27	13.66	16.49	4.47
